# Functional genomics of human bronchial epithelial cells directly interacting with conidia of *Aspergillus fumigatus*

**DOI:** 10.1186/1471-2164-11-358

**Published:** 2010-06-04

**Authors:** Pol Gomez, Tillie L Hackett, Margo M Moore, Darryl A Knight, Scott J Tebbutt

**Affiliations:** 1 UBC James Hogg Research Centre, Providence Heart + Lung Institute, St. Paul's Hospital, Vancouver, BC, Canada; 2 Department of Anesthesiology, Pharmacology and Therapeutics, University of British Columbia, Vancouver, BC, Canada; 3 Department of Biological Sciences, Simon Fraser University, Burnaby, BC, Canada; 4 Department of Medicine, Division of Respiratory Medicine, University of British Columbia, Vancouver, BC, Canada

## Abstract

**Background:**

*Aspergillus fumigatus (A. fumigatus) *is a ubiquitous fungus which reproduces asexually by releasing abundant airborne conidia (spores), which are easily respirable. In allergic and immunocompromised individuals *A. fumigatus *can cause a wide spectrum of diseases, including allergic bronchopulmonary aspergillosis, aspergilloma and invasive aspergillosis. Previous studies have demonstrated that *A. fumigatus *conidia are internalized by macrophages and lung epithelial cells; however the exact transcriptional responses of airway epithelial cells to conidia are currently unknown. Thus, the aim of this study was to determine the transcriptomic response of the human bronchial epithelial cell line (16HBE14o-) following interaction with *A. fumigatus *conidia. We used fluorescence-activated cell sorting (FACS) to separate 16HBE14o- cells having bound and/or internalized *A. fumigatus *conidia expressing green fluorescent protein from cells without spores. Total RNA was then isolated and the transcriptome of 16HBE14o- cells was evaluated using Agilent Whole Human Genome microarrays.

**Results:**

Immunofluorescent staining and nystatin protection assays demonstrated that 16HBE14o- cells internalized 30-50% of bound conidia within six hrs of co-incubation. After FAC-sorting of the same cell culture to separate cells associated with conidia from those without conidia, genome-wide analysis revealed a set of 889 genes showing differential expression in cells with conidia. Specifically, these 16HBE14o- cells had increased levels of transcripts from genes associated with repair and inflammatory processes (*e.g.*, matrix metalloproteinases, chemokines, and glutathione S-transferase). In addition, the differentially expressed genes were significantly enriched for Gene Ontology terms including: chromatin assembly, G-protein-coupled receptor binding, chemokine activity, and glutathione metabolic process (up-regulated); cell cycle phase, mitosis, and intracellular organelle (down-regulated).

**Conclusions:**

We demonstrate a methodology using FACs for analyzing the transcriptome of infected and uninfected cells from the same cell population that will provide a framework for future characterization of the specific interactions between pathogens such as *A. fumigatus *with human cells derived from individuals with or without underlying disease susceptibility.

## Background

*Aspergillus fumigatus *is a ubiquitous filamentous fungus found in soil and compost heaps, as well as in most indoor environments [1]. It achieves widespread dispersal by asexual reproduction through the release of haploid conidia (also known as conidiospores), but can also reproduce sexually [2]. Although *A. fumigatus *is not the most prevalent fungal species worldwide, it is one of the most ubiquitous fungi due to the large number of airborne conidia it releases. Estimates of 1 to 100 colony forming units of *A. fumigatus *per cubic metre have been reported for indoor and outside air, and this widespread distribution ensures that all humans are likely to inhale at least hundreds of conidia each day [3,4]. The small size of the *A. fumigatus *conidia (2 μm) allows them to reach the innermost areas of the lung, including the alveoli [5]. *A. fumigatus *conidia have been shown to be efficiently internalized by cultured murine alveolar macrophages [6] and the human alveolar type II pneumocyte cell line, A549 [7]. In immune compromised individuals, *A. fumigatus *can cause a spectrum of diseases which range from local hypersensitivity reactions to often fatal systemic mycoses [5,8]. Although infections by *A. fumigatus *have been described in other sites of the body, the respiratory tract is the main route of entry and site of infection. The three predominant forms of disease caused by *A. fumigatus *are: allergic bronchopulmonary aspergillosis (ABPA), which is prevalent in up to 5% of asthmatic and 10% of cystic fibrosis patients; aspergilloma, a condition in which fungal mycelia grow as a mass in pre-existing lung cavities; and invasive pulmonary aspergillosis (IPA), a life-threatening systemic mycosis in immunocompromised individuals [3,9]. Despite the importance of the host's response in these conditions, the mechanisms involved in each of these diseases are still not completely understood.

The bronchial epithelium is the first point of contact and barrier to inhaled environmental particulates. A recent electron microscopy study by Amitani and Kawanami [10], using an organ culture model, showed three possible pathways by which *A. fumigatus *conidia invade the epithelial barrier: (1) penetration of hyphae through the intercellular spaces in the epithelium; (2) direct penetration of hyphae through epithelial cells; and (3) internalization of conidia within epithelial cells. Once internalized, conidia are ingested by acidified phagolysosomes and are degraded, although a small number of internalized conidia may survive and germinate within the phagolysosomes [7]. Hence, uptake of *A. fumigatus *conidia into lung epithelial cells may represent a mechanism of immune evasion.

Clearly, the cellular response of the bronchial epithelium following internalization of *A. fumigatus *conidia is important to understand, particularly since the interaction may be important in mediating the allergenic properties of the mould as well as the host immune response. This would be particularly pertinent to diseases such as asthma, where aberrant epithelial responses to injury or stress may be important drivers of disease pathogenesis [11].

The aim of the present study was to assess the cell-specific transcriptional response of cultured human bronchial epithelial cells following direct interaction with *A. fumigatus *conidia. To assess the response of those cells in direct interaction with conidia, we used fluorescence-activated cell sorting (FACS) to specifically isolate cells associated with a recombinant strain of *A. fumigatus *that constitutively expresses green fluorescent protein (GFP) from an epithelial culture. We then applied whole-genome expression microarray technology to assess transcriptional differences between these cell populations.

## Results

### *A. fumigatus *conidia are internalized by human bronchial epithelial cell monolayers

**Figure 1:**
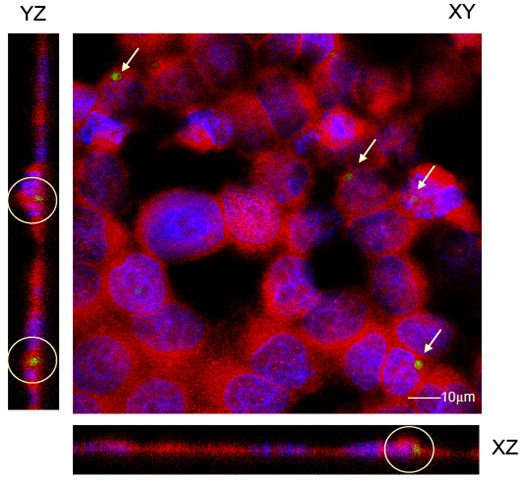
**Localization of *A. fumigatus *conidia within the epithelial cell monolayer**. GFP-expressing *A. fumigatus *conidia and 16HBE14o- cells were co-incubated for 6 hrs, fixed and then stained with DAPI to localize nuclei (blue) and an antibody to adherens junction protein, E-cadherin, to localize cell membranes (red), which were visualized using confocal microscopy. GFP-expressing *A. fumigatus *conidia (green) found within 16HBE14o- cells, are highlighted by the white arrows in the XY plane and white circles in the XZ and YZ planes.

To determine if human bronchial epithelial cells could internalize *A. fumigatus *conidia, 16HBE14o- cell monolayers were co-cultured with GFP-expressing *A. fumigatus *conidia and internalization was assessed by scanning confocal microscopy. As shown in the representative 3-dimensional reconstruction, the YZ and XZ planes of the images demonstrate that a population of 16HBE14o- cells contained intracellular *A. fumigatus *conidia following 6 hrs of co-incubation (Figure 1). The internalization process did not affect the cell surface expression of the epithelial specific adherens junction protein, E-cadherin, suggesting that the epithelium was not stimulated.

**Figure 2:**
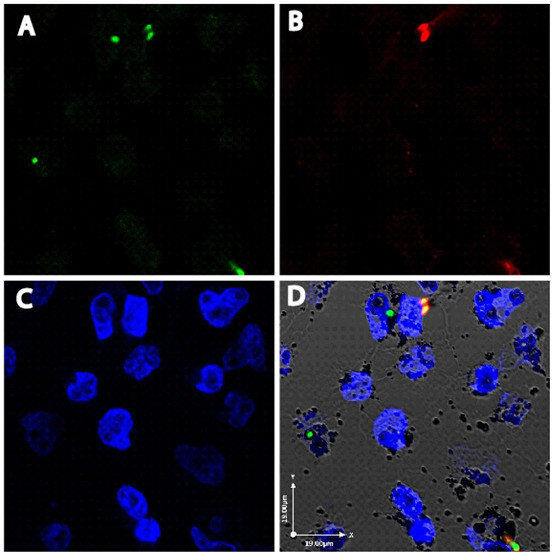
**Differential staining of extracellular and internalized conidia by anti-*A. fumigatus *antibody**. GFP-expressing *A. fumigatus *conidia and 16HBE14o- cells were co-incubated for 6 hrs, fixed and then stained with DAPI, to label cell nuclei, and a polyclonal anti-*A. fumigatus *antibody, to label extracellular conidia, before visualization by confocal microscopy. One representative field is shown in the following channels: A) wavelength 495 nm for GFP (green); B) wavelength 594 nm for anti-*A. fumigatus *antibody (red); C) wavelength 405 nm for DAPI (blue); D) merged GFP, anti-*A. fumigatus *antibody and DAPI image. Conidia not labeled by the anti-*A. fumigatus *antibody, and therefore only visible in the green but not red channel, were considered to be internalized by 16HBE14o- cells, allowing quantification of internalization.

**Figure 3:**
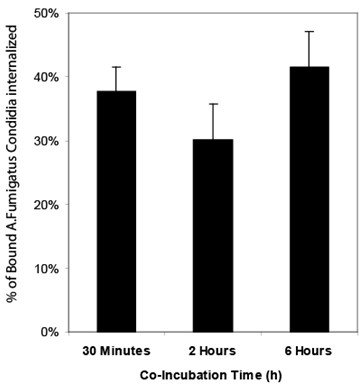
**Extent of internalization of *A. fumigatus *conidia by epithelial cells determined by nystatin protection assay**. *A. fumigatus *conidia and 16HBE14o- cells were co-incubated for the indicated times, then treated with nystatin-supplemented or nystatin-free media for 3 hrs. Cells were lysed, and recovered conidia were plated to count colony forming units. At each time point, the rate of internalization was determined as the number of colony forming units recovered from nystatin-treated wells divided by the number from control-treated wells. The mean % of internalization ± SD from three biological replicates is shown for each time point.

To further confirm the internalization of *A. fumigatus *conidia by 16HBE14o- cells, we measured immunolabelling of extracellular and internalized *A. fumigatus *conidia [6]. As demonstrated by the representative images in Figure 2, following 6 hrs of co-culture ~50% of *A. fumigatus *conidia were found to be internalized and therefore were not labelled by the anti-*A. fumigatus *antibody. The images also revealed that there was wide variation in the number of conidia internalized, with some cells interacting with several conidia while others did not appear to interact with any. To further characterize the interaction, we used the nystatin protection assay as an independent method to measure conidia internalization by cultured cells over time [6]. The number of internalized *A. fumigatus *conidia over a time course of 30 mins, 2 hrs and 6 hrs was 38%, 30% and 41%, respectively (Figure 3). These differences were not statistically significant. Overall, our results indicate that 16HBE14o- cells internalize more than a third of the *A. fumigatus *conidia that bind to the cells.

### Isolation of human bronchial epithelial cells following interaction with *A. fumigatus *conidia using FACS

**Figure 4:**
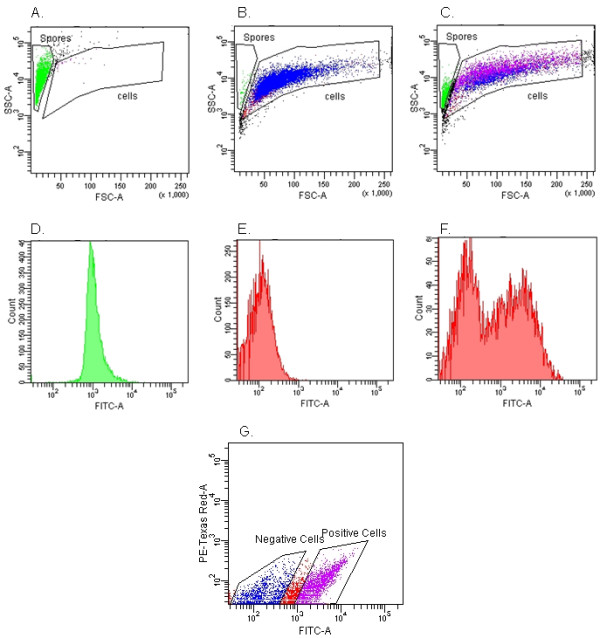
**FACS analysis of *A. fumigatus *conidia and epithelial cells incubated alone or together**. Cultures of GFP-expressing *A. fumigatus *conidia alone (A), 16HBE14o- cells alone (B), or cells and conidia incubated together for 6 hrs (C) were analyzed by flow cytometry, using forward scatter (FSC) and side scatter (SSC). The gates defining conidia (labeled 'spores', coloured green) and 16HBE14o- cells (labeled 'cells', coloured blue and purple) were set manually based on the observed distributions by FSC and SSC. Histograms showing the FITC channel signal intensity from the selected gates for (D) conidia alone, (E) 16HBE14o- cells alone, and (F) co-cultures of 16HBE14o- cells and conidia. The bimodal distribution of FITC intensity allowed the sorting of 16HBE14o- cells positive for GFP from negative cells (Figure 4G).

We took advantage of the observation that not all epithelial cells contained conidia and used FACS to separate the 16HBE14o- cells into two populations: epithelial cells directly interacting with GFP-*A. fumigatus *conidia, and those with no direct contact. As shown in Figure 4A-C, we were able to easily distinguish cells from conidia by gating on forward (FSC) and side (SSC) scatter. Identified conidia and cells were further analyzed by determining GFP fluorescence intensity as demonstrated for *A. fumigatus *conidia alone (Figure 4D), 16HBE14o- cells alone (Figure 4E) and co-incubated *A. fumigatus *conidia and 16HBE14o- cells (Figure 4F). Other gates were set based on the fluorescence intensities of GFP and PE Texas Red, and this permitted selection of cells associated with conidia (Figure 4G).

**Figure 5:**
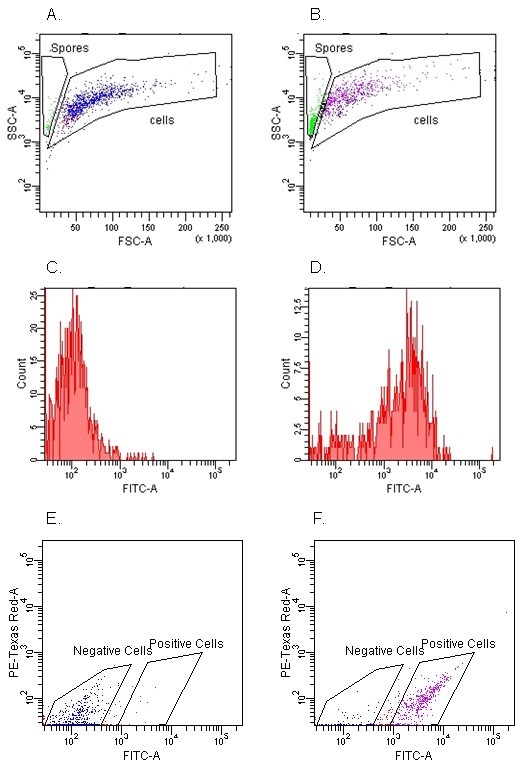
**Re-analysis of positive and negative sorted samples to determine the accuracy of sorting**. FACS-sorted negative (A) and positive (B) 16HBE14o- cell samples were re-analyzed to determine the accuracy of sorting based on fluorescence intensity signals. Histograms showing the FITC channel signal intensity from the selected gates for negative (C) and positive (D) 16HBE14o- cells for GFP-expressing conidia. Dot plots showing fluorescence intensity in FITC and PE-Texas Red channels associated with negative (E) and positive (F) 16HBE14o- cells for GFP-expressing conidia.

**Figure 6:**
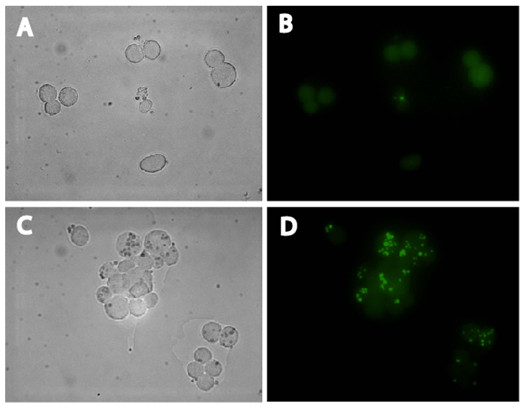
**Microscopic visualization of negative and positive sorted samples to determine the accuracy of sorting**. FAC-sorted negative (A, B) and positive (C, D) cell samples were visualized by differential interference contrast (DIC) and fluorescence microscopy to verify the accuracy of sorting based on fluorescence intensity signals. Shown for each sample are DIC images (A, C) and green fluorescence images (B, D). Individual 16HBE14o- cells are clearly visible in the DIC images, while GFP-expressing *A. fumigatus *conidia present as bright green spots in the green fluorescence channel. The majority of cells in the negative samples were free of conidia, while most cells in positive samples were associated with at least one conidia spore.

As demonstrated in Figure 5, positive and negative sorted cell populations were re-analyzed to determine the average sorting accuracy. The average sorting accuracy was calculated to be 75.7%, although there was a statistically significant difference in sorting accuracy depending on whether positive or negative cell populations were examined (69.7% vs. 81.6%, respectively, p = 0.0016). Confocal microscopy of the two cell populations (Figure 6) confirmed the FACS data.

### Functional genomics analysis reveals differential gene expression between bronchial epithelial cells directly interacting with conidia compared to cells alone

Expression data were obtained from ~41,000 gene probes on each of eight arrays (four biological replicate experiments, each comprising two paired sorted populations) and submitted to the Gene Expression Omnibus (Series GSE16627). Hierarchical clustering demonstrated that 16HBE14o- cells positive for GFP-expressing *A. fumigatus *conidia (positive cells) and negative cells showed a high level of concordance, as indicated by their similar gene expression clustering profiles (data not shown). Furthermore, these arrays formed pairs based on their co-incubation sample, indicating that positive and negative populations sorted from each co-incubated sample were generally more similar to each other than to other sorted populations, reflecting their shared heritage.

Using GeneSpring GX 7.3.1 software, 20,541 probes exhibited significant expression in either positive or negative samples. Of these, 889 showed differential expression between positive and negative paired populations (*i.e.*, samples from the same co-incubation), based on a paired t-test p-value of 0.05 and a fold change of 1.1 or greater. None of these genes retained significant p-values following multiple-testing correction implemented within the GeneSpring software (Benjamini-Hochberg method). Within this list of genes of interest, 376 were up-regulated in positive sorted samples, while 513 showed down-regulated expression in conidia-positive cells compared to conidia-negative cells. The complete list of genes is provided in Additional file 1. To determine the major biological themes found in this dataset, we looked for Gene Ontology (GO) classifications that were over-represented in these two gene lists using GOEAST [12]. In both cases, specific GO categories were significantly over-represented, with p-values below 10^-12 ^(Table 1). The up-regulated GO list was enriched for terms associated with nucleosome organization, chromatin assembly, as well as chemokines and glutathione, important for inflammation and repair processes. The down-regulated GO list was enriched for terms associated with mitosis, cell cycle progression, and intracellular organelle biology. The annotated genes showing the greatest up- or down-regulation are shown in Table 2, and include matrix metalloproteinases, chemokines, and glutathione S-transferase.

**Table 1:** Over-represented Gene Ontology terms in the lists of differentially expressed genes

List of Terms Up-Regulated in Positive Cells (376 genes)			
			
GOID	Ontology	Term	P-value
GO:0000786	CC	nucleosome	9.52E-12
GO:0006334	BP	nucleosome assembly	1.19E-11
GO:0008009	MF	chemokine activity	3.10E-05
GO:0001664	MF	G-protein-coupled receptor binding	3.30E-05
GO:0006749	BP	glutathione metabolic process	3.70E-05
GO:0009611	BP	response to wounding	1.08E-03
GO:0005125	MF	cytokine activity	5.37E-03
GO:0009605	BP	response to external stimulus	5.76E-03
GO:0006954	BP	inflammatory response	1.07E-02
GO:0006955	BP	immune response	2.16E-02
			
**List of Terms Down-Regulated in Positive Cells (513 genes)**			
			
**GOID**	**Ontology**	**Term**	**P-value**
GO:0000279	BP	M phase	5.07E-13
GO:0043229	CC	intracellular organelle	8.96E-13
GO:0022402	BP	cell cycle process	5.26E-12
GO:0005634	CC	nucleus	1.43E-11
GO:0000278	BP	mitotic cell cycle	4.51E-11
GO:0044424	CC	intracellular part	8.33E-11
GO:0048285	BP	organelle fission	1.59E-10
GO:0005694	CC	chromosome	2.54E-07
GO:0006996	BP	organelle organization	4.31E-07
GO:0007098	BP	centrosome cycle	4.49E-05

Selected terms, belonging to the biological process (BP), molecular function (MF), and cellular component (CC) ontologies, are indicated, and the p-value for enrichment in the appropriate list is shown.

**Table 2:** Genes showing the highest fold-changes between positive and negative sorted cells

Gene Symbol	Gene Name	Fold Change	P-value
MMP1	matrix metallopeptidase 1 (interstitial collagenase)	1.69	2.94E-03
MMP3	matrix metallopeptidase 3 (stromelysin 1, progelatinase)	1.63	1.07E-02
CCL5	chemokine (C-C motif) ligand 5	1.60	3.38E-02
CCL3	chemokine (C-C motif) ligand 3	1.58	2.53E-02
ROCK1	Rho-associated, coiled-coil containing protein kinase 1	1.51	5.00E-02
GREM1	gremlin 1, cysteine knot superfamily, homolog (Xenopus laevis)	1.50	1.01E-02
ACTN2	actinin, alpha 2	1.46	1.22E-02
FBXO32	F-box protein 32	1.43	1.64E-02
MGST1	microsomal glutathione S-transferase 1	1.43	2.73E-03
EVI2B	ecotropic viral integration site 2B	1.40	8.27E-04
LRRFIP1	leucine rich repeat (in FLII) interacting protein 1	-1.33	2.96E-02
SFTPC	surfactant protein C	-1.34	3.97E-02
C9orf100	chromosome 9 open reading frame 100	-1.35	2.14E-03
LIPE	lipase, hormone-sensitive	-1.35	2.67E-02
TERT	telomerase reverse transcriptase	-1.36	1.54E-02
MTERFD3	MTERF domain containing 3	-1.37	3.53E-03
SFTPC	surfactant protein C	-1.37	2.55E-02
KIAA0802	KIAA0802	-1.37	1.01E-02
SLC1A3	solute carrier family 1 (glial high affinity glutamate transporter), member 3	-1.39	6.14E-04
NDE1	nudE nuclear distribution gene E homolog 1 (A. nidulans)	-1.50	2.98E-02

The fold change is the ratio of the expression level in the two conditions, with positive numbers representing up-regulation in positive samples, while negative numbers indicate down-regulation in the positive samples, compared to negative samples. The paired t-test p-value for differential expression is also shown. Probes lacking annotations were excluded from this list.

We used Gene Set Enrichment Analysis (GSEA) to establish further evidence in support of our findings and thus compensate for the lack of statistical significance after multiple hypothesis testing [13]. Leading edge analysis of 2,398 gene sets resulted in 177 sets significantly associated with our up-regulated gene data and 54 sets significantly associated with our down-regulated gene data (FDR ≤ 0.25 and p-value ≤ 0.05). These gene sets are provided in Additional file 2 and are consistent with the GO classifications (Table 1) obtained from our original list of 889 differentially expressed genes.

## Discussion

This study demonstrates that cultured 16HBE14o- human bronchial epithelial cells are capable of rapidly internalizing *Aspergillus fumigatus *conidia. The number of conidia taken up by individual cells was variable and we exploited this variability to successfully separate cells that were directly interacting with and/or had internalized spores from those that had not. A microarray-based gene expression analysis on these two populations identified 376 genes that were up-regulated and 513 genes that were down-regulated in epithelial cells that were directly interacting with and/or had internalized conidia, relative to cells from the same co-incubated sample. Our data suggest that the direct interaction and/or internalization of common fungal spores such as *Aspergillus fumigatus *conidia has measurable, if modest, effects on the transcriptome of human bronchial epithelial cells.

The importance of professional phagocytes in the clearance of inhaled *A. fumigatus *conidia has motivated multiple studies on the internalization of conidia by these cells [14-19]. Fewer studies have investigated *A. fumigatus *internalization by non-professional phagocytes such as epithelial cells [6,7,20-23]. We and others have demonstrated the uptake of *A. fumigatus *conidia in the type-II pneumocyte cell line, A549 [21], primary tracheal epithelial cells, and umbilical vein endothelial cells (HUVEC) [23]. These studies showed that A549 cells and HUVECs internalized 30% and 50%, respectively, of bound *A. fumigatus *conidia following 6 hrs of co-culture [6]. In the present study, we show that 16HBE14o- cells internalized between 30-50% of *A. fumigatus *conidia within 6 hrs of exposure, consistent with previous studies focused on other non-professional phagocyte cell lines [6,7,20,21,23]. This also compares favourably with Botterel *et al*. (2008), who showed that co-incubation of *A. fumigatus *with primary human nasal epithelial cells for 4 hrs yielded an internalization rate of about 20% [20]. These studies also demonstrated delayed germination but long term survival of internalized conidia, suggesting that normally non-phagocytic epithelial cells may act as reservoirs for invading *A. fumigatus *conidia [6,7,20].

For our quantitative studies on *A. fumigatus *conidia internalization by 16HBE14o- cells we used a relatively high multiplicity of infection (MOI) of 10 conidia per human cell. The high MOI was chosen to maximize the number of infected cells and while this represents a much greater dose than would be found in the lung, it is in line with previous *in vitro *studies [6,7,20].

Using FACS and GFP-expressing *A. fumigatus *conidia we were able to sort epithelial cells derived from a single culture that contained bound and/or internalized conidia from those without conidia, allowing us to examine their gene expression profiles using a paired analysis. To our knowledge, this sorting of otherwise identical cells based on their association with or without a pathogen is a novel application, one that could be applied to a range of pathogens and different cell types. Although FACS analysis could not discriminate the number of *A. fumigatus *conidia associated with each 16HBE14o- cell, fluorescence microscopy indicated that many of the positively sorted 16HBE14o- cells were associated with high numbers of spores.

Numerous studies have applied whole genome transcriptional profiling to interactions between mammalian hosts and microbes [24-27]; however, to our knowledge, there is no study on changes to the transcriptome of human bronchial epithelial cells as a consequence of interacting with fungal conidia. By analyzing the transcriptomes of 16HBE14o- cells co-incubated with *A. fumigatus *conidia, 889 genes were found to be differentially expressed between the positive and negative cell populations although the magnitude of the fold changes in gene expression were generally small. The reasons for this may be related in part to the methodology employed: sorting based on fluorescence intensity is not 100% accurate, and the conidia-positive population contained a proportion of negative cells and *vice-versa*. This would reduce the magnitude of the observed differences in gene expression between two populations. Nevertheless, the modest transcriptional response following direct interaction with *A. fumigatus *conidia may also indicate that the host cell does not respond dramatically to conidial uptake thereby providing a protected niche for conidial development. This is supported by other data. For example, Wasylnka *et al*. showed that interaction and even internalization of *A. fumigatus *conidia by A549 cells had little effect on cellular physiology [7]. Moreover, binding of *A. fumigatus *conidia to A549 cells has been shown to induce only minor up-regulation of immune response effectors [28]. A recent report indicates that germ tube growth, rather than interaction with conidia, leads to an activation of immune responses in A549 cells [29]. In addition, Aimanianda *et al. *showed that the lack of immune response in macrophages and dendritic cells to fungal conidia was due to the presence of the surface hydrophobin protein layer (rodlet layer) on conidia [30]. Loss of the rodlet layer during germination unmasked fungal antigens and resulted in an innate immune response in professional phagocytes. Our data indicate that, like professional phagocytes, epithelial cells may not mount a strong immunological response to direct interaction with conidia (within 6 hrs of exposure).

Nevertheless, Gene Ontology (GO) analysis of the list of 889 genes demonstrated a coherent and significant response in cells exposed to conidia, and this response was largely re-capitulated using Gene Set Enrichment Analysis (GSEA) on our entire microarray data set. The GO terms enriched for both the up- and down-regulated genes point to fundamental changes associated with the response to fungal spores. For example, chemokine activity and receptor binding ontologies were prevalent in up-regulated genes, consistent with their established role in responding to fungal invasion [31-33]. The list of down-regulated genes was enriched for many GO terms relating to mitosis and cell cycle progression, indicating that cells reduce their rate of proliferation in response to direct interaction with conidia which may be part of a general stress response. Cell cycle arrest in response to fungal alkaloids has been previously reported [34,35].

Many of the individual genes displaying the highest fold changes belonged to the ontologies identified by GO term enrichment analysis, lending further support to the relevance of the identified themes. Amongst those showing the highest degree of up-regulation in response to direct conidial interaction are two chemokines (CCL3, CCL5) and two matrix metalloproteinases (MMP1, MMP3), as well as a glutathione transferase (MGST1). CCL3 is an inflammatory chemokine responsible for recruitment of leukocytes to sites of infection, and promotes a Th1 phenotype in lymphocytes [36,37]. CCL5 is strongly chemotactic for eosinophils [38-40]. The expression of CCL3 has previously been shown to be increased in mouse lungs and rat alveolar macrophages following exposure to *A. fumigatus *conidia [41,42].

Positive associations between MMPs and *A. fumigatus *have been reported previously in a study involving conidial infection of corneas [43], and MMP1 expression has been shown to be higher in sputum cells of asthmatics relative to controls [44]. Microsomal glutathione S-transferase 1 (MGST1), found to be up-regulated in cells interacting with *A. fumigatus *conidia, is the prototypical member of the glutathione S-transferase gene family [45] and has a role in protecting cell from oxidative damage [46].

The experiments in this study used submerged monolayer cultures of a human bronchial epithelial cell line, 16HBE14o-. Some characteristics and responses of these cells are likely to be different from both primary cell cultures and airway cells *in situ *in the lung. Nevertheless, our approach has the advantage in that it enables us to identify airway cell responses independently of other cell types (*e.g.*, immune, phagocytic and mesenchymal cells).

## Conclusions

We demonstrate the use of FACs to isolate human airway epithelial cells that bind and/or internalize *A. fumigatus *conidia in co-culture systems for subsequent assessment of the specific transcriptional responses of cells directly interacting with conidia. The ability to identify differentially expressed genes within a single culture using FACs has potential uses to further investigate the response of other cell types to specific interactions with pathogens. Our ability to better understand the interaction of inhaled *A. fumigatus *conidia with airway epithelial cells will help provide insights into the mechanisms of several diseases associated with this ubiquitous mould.

## Methods

### *A. fumigatus *expressing GFP and culture conditions

All experiments were performed using a green fluorescent protein (GFP) expressing strain of *A. fumigatus *previously developed by Wasylnka and Moore [6]. Briefly, the *A. fumigatus *strain ATCC 13703 (American Type Culture Collection, Manassas, VA) was transformed by electroporation with a plasmid containing the sequence-optimized sGFP gene driven by the *Aspergillus nidulans *promoter P *gdp*. This construct yielded stable, high expression of GFP in fungal conidia and hyphae [6]. For long-term storage, conidia were kept at 4°C on MYPD agar medium (0.3% malt extract, 0.3% yeast extract, 0.5% peptone, 0.5% dextrose, 1.5% agar) supplemented with hygromycin (100 μg/ml) to suppress the growth of wild-type fungus. To obtain fresh conidia for each experiment, conidia were transferred from the stock and streaked onto MYPD plates, and grown for three days at 37°C. Mature conidia were harvested by gently scrubbing plates with sterile cotton swabs with phosphate-buffered saline (PBS) with 0.05% Tween-20 (PBS-T). The resulting conidia suspension was vortexed and passed through a glass-wool plugged funnel to eliminate hyphae. The suspension was again vortexed, pelletted, and resuspended in 1 ml PBS. Using this procedure, one MYPD plate typically yielded more than 10^9 ^conidia for co-incubation experiments, quantified using a hemocytometer.

### 16HBE14o- cell line and culture conditions

The 16HBE14o- transformed bronchial epithelial cell line was obtained from Dr. D. Gruenert (University of Vermont, Burlington, VT). This cell line retains key characteristics of human bronchial epithelium, including the formation of a monolayer, tight junctions, and directional ion transport [47]. 16HBE14o- cultures were maintained in low-glucose- Dulbecco's Modified Eagles Medium (DMEM) containing 10% FBS, 2 nM L-glutamine, 100 U/ml penicillin and 100 μg/ml streptomycin at 37°C in a humidified 5% CO_2 _atmosphere. Cells were seeded and grown to confluence (approximately 1 × 10^6 ^cells) in 6 well tissue culture plates (Corning Costar, Cambridge, MA) for isolation by FACS or on Falcon 8-chamber tissue culture slides (approximately 1 × 10^5 ^cells (BD Biosciences, Franklin Lakes, NJ) for confocal microscopy.

### 16HBE14o- and *A. fumigatus *conidia co-culture and analysis by confocal microscopy

Confluent 16HBE14o- monolayers seeded in Falcon 8-chamber tissue culture slides were co-incubated with *A. fumigatus *conidia (MOI 10 conidia/cell) for 6 hrs (DMEM containing 10% FBS, 2 nM L-glutamine, 100 U/ml penicillin and 100 μg/ml streptomycin) at 37°C. Cultures were then washed three times in phosphate buffered saline (PBS) to remove unbound *A. fumigatus *conidia, and then fixed in 4% paraformaldehyde (Fisher, Ottawa, ON) in PBS for 20 mins at room temperature. Slides were then blocked with 1% bovine serum albumin (BSA) in PBS for 30 mins to block non-specific staining and then incubated with 4 μg/ml polyclonal rabbit anti-human E-Cadherin antibody (Santa Cruz Biotechnology, Santa Cruz, CA) in PBS for two hrs at room temperature. Following washing with PBS containing 0.1% Tween-20, cultures were then incubated with goat anti-rabbit IgG antibody conjugated with Alexa Fluor 594 (Invitrogen, Carlsbad, CA) diluted 1:500 in PBS. Following the final washes with PBS-0.1% Tween-20, cells were incubated with (1 μg/ml) DAPI (4',6-diamidino-2-phenylindole) in PBS for 15 mins to label cell nuclei. Chamber slides were then dehydrated in ethanol before the addition of Cytoseal 60 mounting medium (Electron Microscopy Sciences, Hatfield, PA) and cover-slipping. Specimens were visualized using a Multiphoton Confocal Microscope System at the UBC James Hogg Research Centre (Vancouver, BC). Images were acquired using a Leica AOBS SP2 laser scanning confocal microscope (Leica, Heidelberg, Germany) with Zeiss LSM 510 software, version 3.2. The laser lines used were 405 nm (for DAPI), 488 nm (for GFP), 594 nm (for Alexa 594) and these excitation beams were produced by UV diode, Ar and HeNe lasers (Leica AOBS SP2 module), respectively. Emission signals were captured sequentially to minimize signal bleed-through and the confocal pinhole was set at one Airy disk. Images (8 bit) were frames-averaged 3-4 times to minimize the noise and the pixel dimensions used in this image acquisition process satisfied the Nyquist sampling criteria. The images were overlaid and the contrast enhancements were performed using Volocity software (Improvisions, Boston, USA).

### Quantification of *A. fumigatus *conidia uptake by 16HBE14o- cells using immunofluorescent staining

Co-cultures of 16HBE14o- cell with *A. fumigatus *conidia in Falcon 8-chamber tissue culture slides were processed, fixed and blocked as described above. Co-cultures were then incubated with a polyclonal rabbit antibody raised against *A. fumigatus *cell wall components, which was previously developed by Wasylnka and Moore [6]. This primary antibody was diluted 1:75 in PBS with 10% goat serum and incubated with the co-incubated cultures overnight at 4°C. Chambers were washed three times with PBS before treatment with secondary goat anti-rabbit IgG Alexa Fluor 594 (Invitrogen, Carlsbad, CA) diluted 1:500 in PBS. Following another round of washes, cells were labeled with DAPI (1 μg/ml) in PBS for 5 mins before a single final wash in PBS. Following staining the chamber wells were then dried using sequential one minute immersions in 70%, 95%, and 100% ethanol, before cover-slipping. Images were acquired using a Leica AOBS SP2 laser scanning confocal microscope with Zeiss LSM 510 software as described above. Quantification of *A. fumigatus *conidia uptake was based on the fact that only extracellular conidia are labeled, but internalized ones are not. Thus the rate of internalization was calculated as the ratio of the number of conidia showing only green fluorescence (*i.e.*, internalized conidia) to the number of conidia labeled with antibody or not (*i.e.*, total number of conidia).

### Quantification of *A. fumigatus *conidia uptake by 16HBE14o- cells using nystatin protection assay

Confluent monolayers of 16HBE14o- cells in Falcon 8-chamber tissue culture slides were co-incubated with *A. fumigatus *for 30 mins, 2 hrs, and 6 hrs at 37°C, and then washed three times in PBS to remove unbound conidia. For each time point, cultures were then incubated for 3 hrs at 37°C in either 1 ml of DMEM supplemented with 100 μg nystatin, or DMEM alone. This treatment with nystatin has previously been shown to kill exposed *A. fumigatus *conidia, but is not toxic for human cells [6]. Wells were washed again three times with PBS and the cells were incubated for 10 mins at 37°C in 3 ml lysis buffer consisting of 0.1% sodium dodecyl sulphate (SDS) and 1% Triton X-100 in deionized water. Colony-forming units were counted by collecting 1 ml of conidia-containing cell lysates, diluting 10×, 100×, and 1000× in PBS, and plating 100 μl of each dilution onto MYPD agar plates. These were incubated at 37°C overnight before colonies were counted. The rate of internalization was based on the dilution yielding the greatest number of colonies that remained countable. The internalization rate was expressed as the number of colonies recovered from nystatin-treated wells divided by the number of colonies recovered from untreated wells at each time point.

### Flow cytometry and fluorescence-activated cell sorting (FACS)

Confluent monolayers of 16HBE14o- cells in 6 well tissue culture plates were co-incubated with or without *A. fumigatus *conidia (10^7 ^conidia per well) for 6 hrs at 37°C, and then washed three times in PBS to remove unbound conidia. Cultures were then incubated with 1 ml trypsin-EDTA (Sigma) for five min at 37°C to produce a cell suspension, the trypsin was then neutralized using DMEM containing 10% FBS and removed following centrifugation for five mins at 1000 RPM (168 RCF). The resultant pellets were re-suspended in 2 ml DMEM. For a positive control, a sample of *A. fumigatus *conidia incubated alone in DMEM was also analyzed. Flow cytometry was performed on a BD FACSAria with BD FACSDiva application software version 5.0.2 (BD Biosciences, Franklin Lakes, NJ) paired with FlowJo Version 6.3.2 analysis software (Tree Star Inc., Ashland, OR). The excitation laser was set at 488 nm to induce GFP fluorescence in samples containing conidia. For all samples, forward and side scatter measurements were recorded, and the emission of GFP detected using a 530/30 emission filter and PE-Texas Red detected using a 610/20 emission filter. Gates to select cells of interest were determined using FSC, SSC and fluorescence emission properties, all of which were set manually based on measurements obtained from analyzing 10,000 events from each sample of control cells, co-incubated cells, or *A. fumigatus *conidia alone. The gates identifying cells as positive or negative were used to sort co-incubated cells into two separate populations, representing cells interacting directly with conidia and cells not interacting directly with conidia. Sorted cells were collected into Falcon Tubes (BD Biosciences) containing DMEM to minimize cellular damage before RNA was isolated. The accuracy of cell sorting was determined for each sample by re-analyzing 1000 cells and calculating the percentage of cells falling in the appropriate gate. To further assess the accuracy of cell sorting, representative samples of sorted cells were visualized by fluorescence microscopy to confirm the presence of conidia with cells from the positive populations but not from the negative populations. These representative samples were centrifuged for six min at 1000 RPM (168 RCF) to collect cells, which were re-suspended in 200 μl of PBS, and cytospins made onto poly-L-lysine-coated slides. Specimens were visualized using a Leica DM IRE2 microscope (Leica), equipped with a Hamamatsu ORCA ER digital camera (Hamamatsu Photonics, Bridgewater, NJ) with Wasabi software (Hamamatsu Photonics). Images obtained in differential interference contrast and green fluorescence channels were processed and merged in Adobe Photoshop (Adobe Inc., San Jose, CA) to visualize both sorted 16HBEo- cells and associated *A. fumigatus *conidia.

### RNA preparation and microarray analysis

Upon the completion of cell sorting, each of these paired positive and negative populations were centrifuged for six min at 1000 RPM (168 RCF), the media was removed and cells were re-suspended in 750 μl buffer RLT (Qiagen). These samples were stored at -80°C prior to RNA extraction. RNA extractions were performed using the RNeasy Mini Kit with QIAshredder (Qiagen), following the manufacturer's recommendations. RNA yield from each population was determined using a NanoDrop ND-1000 spectrophotometer (Thermo Scientific, Wilmington, DE). RNA yields ranged from 30 to 100 ng/μl. RNA integrity of the sorted samples was determined using a 2100 Bioanalyzer (Agilent Technologies, Santa Clara, CA), since the effect of the sorting procedure on RNA integrity was not known. The 2100 Bioanalyzer generates an RNA integrity number (RIN) that indicates the level of degradation in a sample, and has been shown to reliably predict the suitability of RNA samples for gene expression analysis [48]. RIN scores ranged from 7.7 to 9.7, indicating high quality RNA suitable for gene expression analysis by microarray [49].

Microarray services were performed by the Prostate Centre Microarray Facility (Vancouver, Canada), an Agilent Certified Service Provider. Human gene expression was analyzed using Agilent Whole Human Genome Oligo Microarrays in the 4 × 44 K format (product number G4112F, design ID 014850, Agilent Technologies). In total, eight arrays were analyzed, representing four biological replicates of co-incubated samples, each yielding paired positive and negative populations. All microarray data have been deposited in the Gene Expression Omnibus (approved GEO Series GSE16627).

Raw data from the eight whole-genome microarrays, covering 41,000 probe features, was further analyzed using GeneSpring GX 7.3.1 microarray analysis software (Agilent Technologies). Raw signals were first normalized by flooring all values smaller than 1 to 1, then dividing each value by the median intensity value of the array. This removes negative values, and corrects for global differences in signal intensities between arrays. Hierarchical clustering was then performed to generate condition trees, highlighting the relationships in the overall expression levels in the different samples. Pearson Correlation, based on average distance between clusters, was used to generate hierarchical trees based on the normalized intensity signals of the 41,000 probes on each array. To select genes of interest, genes showing differential expression between the positive and negative sorted samples were identified. A raw intensity cutoff was applied, retaining only genes showing a value of greater than 100 in at least three of four replicates of either the positive or negative conditions. Since the four replicates of the positive and negative conditions represented biologically paired samples, we generated four replicate ratios for each probe, which was the signal from the positive cells divided by that from the negative cells sorted from the same co-incubation sample. A Student's t-test was then applied to determine if this fold-change ratio differed significantly from 1, at a p-value of less than 0.05, for each probe. Genes that passed this test and showed a fold change of greater than 1.1 were considered as genes of interest for further analysis. This procedure is equivalent to selecting genes based on a volcano plot, but using a paired rather than unpaired t-test.

The gene list identified was analyzed for Gene Ontology (GO) term enrichment. Gene Ontology provides a uniform, controlled vocabulary to annotate gene products in terms of their associated biological process, molecular function, and cellular component [50]. Given a list of genes annotated with GO terms, the statistically significant overrepresentation of specific terms indicates global features of the gene list. The genes identified as up- or down-regulated in the sorted experiments were analyzed using the Gene Ontology Enrichment Analysis Software Toolkit (GOEAST) [12]. Significantly over-represented Gene Ontology terms were identified using the recommended methods (hypergeometric test, Benjamin and Yekutieli false discovery rate correction, threshold p-value of 0.1).

Gene Set Enrichment Analysis (GSEA [13]) was undertaken on the normalized Agilent data set of 8 arrays. Gene set collections used were the c2.all.v2.5.symbols.gmt [Curated] and the c5.all.v2.5.symbols.gmt [Gene ontology]. The number of permutations (gene-set type) was set to 1000, and the results were subjected to leading edge analysis. Gene sets were considered significant if the GSEA p-value was ≤0.05 and the FDR was ≤0.25.

## Competing interests

The authors declare that they have no competing interests.

## Authors' contributions

PG: Design, collection and/or assembly of data, data analysis and interpretation, manuscript writing. TLH: Conception and design, collection and/or assembly of data, data analysis and interpretation, provision of study materials, manuscript writing. MMM: Conception and design, provision of study materials, interpretation, manuscript writing. DAK: Conception and design, interpretation, manuscript writing. SJT: Principal investigator on this project, overall conception and design, provision of study materials, analysis and interpretation, supervision, manuscript writing. All authors read and approved the final manuscript.

## Supplementary Material

Additional file 1 **Supplementary Table S1: Genes showing differential expression between positive and negative cell populations**. 889 genes were identified as showing differential expression based on a paired t-test (p-value cutoff of 0.05) and a fold change cutoff of 1.1. The fold change for each gene is expressed as the ratio of expression between the two populations, with positive numbers indicating higher expression in the positive population while negative numbers indicate higher expression in the negative population. The p-values indicated are not adjusted for multiple testing. The genes were sorted by fold change. Click here for file

Additional file 2 **Supplementary Table S2: Gene Set Enrichment Analysis (GSEA)**. 177 gene sets were identified as sharing up-regulated genes with our data and 54 gene sets were identified as sharing down-regulated genes (NOM p-value ≤ 0.05 and FDR q-value ≤ 0.25). Click here for file
