# Supplementary material for: Functional genomics of human bronchial epithelial cells directly interacting with conidia of Aspergillus fumigatus
Source: BMC Genomics. 2010 Jun 4;11:358. doi: 10.1186/1471-2164-11-358 (PMC2897809; doi:10.1186/1471-2164-11-358)
Supplement: Additional file 1 — Supplementary Table S1: Genes showing differential expression between positive and negative cell populations. 889 genes were identified as showing differential expression based on a paired t-test (p-value cutoff of 0.05) and a fold change cutoff of 1.1. The fold change for each gene is expressed as the ratio of expression between the two populations, with positive numbers indicating higher expression in the positive population while negative numbers indicate higher expression in the negative population. The p-values indicated are not adjusted for multiple testing. The genes were sorted by fold change. [file 1471-2164-11-358-S1.DOCX]

**Supplementary Table 1: Genes showing differential expression between positive and negative cell populations.** 889 genes were identified as showing differential expression based on a paired t-test (p-value cutoff of 0.05) and a fold change cutoff of 1.1. The fold change for each gene is expressed as the ratio of expression between the two populations, with positive numbers indicating higher expression in the positive population while negative numbers indicate higher expression in the negative population. The p-values indicated are not adjusted for multiple testing. The genes were sorted by fold change.

| Probe ID | Gene Description | Fold Change | P-value |
| --- | --- | --- | --- |
| A_32_P164916 | Homo sapiens mRNA; cDNA DKFZp666D074 (from clone DKFZp666D074) [AL833005] | 1.86 | 7.15E-04 |
| A_23_P9926 | Homo sapiens tetraspanin 10 (TSPAN10), mRNA [NM_031945] | 1.83 | 1.05E-02 |
| A_32_P134634 | ALU5_HUMAN (P39192) Alu subfamily SC sequence contamination warning entry, partial (9%) [THC2271582] | 1.80 | 1.85E-03 |
| A_23_P211468 | AA837799 oe06h09.s1 NCI_CGAP_Ov2 Homo sapiens cDNA clone IMAGE:1385153, mRNA sequence [AA837799] | 1.78 | 2.98E-02 |
| A_32_P164917 | Homo sapiens mRNA; cDNA DKFZp666D074 (from clone DKFZp666D074) [AL833005] | 1.77 | 7.43E-04 |
| A_23_P1691 | Homo sapiens matrix metalloproteinase 1 (interstitial collagenase) (MMP1), mRNA [NM_002421] | 1.69 | 2.94E-03 |
| A_23_P161698 | Homo sapiens matrix metalloproteinase 3 (stromelysin 1, progelatinase) (MMP3), mRNA [NM_002422] | 1.63 | 1.07E-02 |
| A_23_P152838 | Homo sapiens chemokine (C-C motif) ligand 5 (CCL5), mRNA [NM_002985] | 1.60 | 3.38E-02 |
| A_23_P373017 | Homo sapiens chemokine (C-C motif) ligand 3 (CCL3), mRNA [NM_002983] | 1.58 | 2.53E-02 |
| A_24_P852099 |  | 1.54 | 2.81E-02 |
| A_32_P105865 |  | 1.51 | 5.00E-02 |
| A_23_P432947 | Homo sapiens gremlin 1, cysteine knot superfamily, homolog (Xenopus laevis) (GREM1), mRNA [NM_013372] | 1.50 | 1.01E-02 |
| A_23_P360209 | Homo sapiens migration-inducing gene 18 protein mRNA, complete cds. [AY423734] | 1.50 | 2.29E-02 |
| A_23_P115021 | Homo sapiens actinin, alpha 2 (ACTN2), mRNA [NM_001103] | 1.46 | 1.22E-02 |
| A_32_P20997 | AGENCOURT_10278709 NIH_MGC_82 Homo sapiens cDNA clone IMAGE:6592525 5', mRNA sequence [BU561469] | 1.44 | 3.47E-02 |
| A_23_P82814 | Homo sapiens F-box protein 32 (FBXO32), transcript variant 1, mRNA [NM_058229] | 1.43 | 1.64E-02 |
| A_23_P32684 | Homo sapiens PRO1051 mRNA, complete cds. [AF116619] | 1.43 | 3.22E-02 |
| A_23_P36658 | Homo sapiens microsomal glutathione S-transferase 1 (MGST1), transcript variant 1c, mRNA [NM_145791] | 1.43 | 2.73E-03 |
| A_24_P681266 |  | 1.41 | 2.01E-02 |
| A_23_P66694 | Homo sapiens ecotropic viral integration site 2B (EVI2B), mRNA [NM_006495] | 1.40 | 8.27E-04 |
| A_23_P23074 | Homo sapiens interferon-induced protein 44 (IFI44), mRNA [NM_006417] | 1.40 | 1.15E-03 |
| A_23_P40108 | Homo sapiens collagen, type IX, alpha 3 (COL9A3), mRNA [NM_001853] | 1.40 | 1.16E-02 |
| A_24_P918147 | Homo sapiens cDNA FLJ13329 fis, clone OVARC1001795. [AK023391] | 1.40 | 1.97E-02 |
| A_23_P56898 | Homo sapiens kynureninase (L-kynurenine hydrolase) (KYNU), mRNA [NM_003937] | 1.40 | 1.81E-02 |
| A_23_P219045 | Homo sapiens histone 1, H3d (HIST1H3D), mRNA [NM_003530] | 1.39 | 7.77E-03 |
| A_23_P97990 | Homo sapiens protease, serine, 11 (IGF binding) (PRSS11), mRNA [NM_002775] | 1.39 | 2.37E-02 |
| A_23_P389897 | Homo sapiens nerve growth factor receptor (TNFR superfamily, member 16) (NGFR), mRNA [NM_002507] | 1.39 | 4.26E-02 |
| A_32_P161855 | Homo sapiens KIAA1199 (KIAA1199), mRNA [NM_018689] | 1.39 | 3.25E-02 |
| A_24_P257416 | Homo sapiens chemokine (C-X-C motif) ligand 2 (CXCL2), mRNA [NM_002089] | 1.39 | 7.19E-03 |
| A_32_P163215 | BE272930 601171218F1 NIH_MGC_14 Homo sapiens cDNA clone IMAGE:3544661 5', mRNA sequence [BE272930] | 1.39 | 4.79E-02 |
| A_23_P139912 | Homo sapiens insulin-like growth factor binding protein 6 (IGFBP6), mRNA [NM_002178] | 1.39 | 1.42E-03 |
| A_32_P96752 | AW946823 RC2-ET0022-080500-012-b10 ET0022 Homo sapiens cDNA, mRNA sequence [AW946823] | 1.38 | 4.97E-03 |
| A_24_P256380 | Homo sapiens chromosome 1 open reading frame 139 (C1orf139), transcript variant 1, mRNA [NM_024911] | 1.37 | 7.51E-03 |
| A_24_P170667 | Homo sapiens AT rich interactive domain 5B (MRF1-like), mRNA (cDNA clone IMAGE:30345306), partial cds. [BC066345] | 1.36 | 4.16E-02 |
| A_24_P183150 | Homo sapiens chemokine (C-X-C motif) ligand 3 (CXCL3), mRNA [NM_002090] | 1.36 | 2.26E-03 |
| A_23_P212800 | Homo sapiens fibroblast growth factor 5 (FGF5), transcript variant 1, mRNA [NM_004464] | 1.36 | 1.31E-02 |
| A_32_P146815 | Homo sapiens cDNA clone IMAGE:30374677, partial cds. [BC062473] | 1.36 | 3.81E-03 |
| A_23_P149153 | Homo sapiens phosphodiesterase 4D interacting protein (myomegalin) (PDE4DIP), transcript variant 3, mRNA [NM_022359] | 1.36 | 7.31E-03 |
| A_23_P169039 | Homo sapiens snail homolog 2 (Drosophila) (SNAI2), mRNA [NM_003068] | 1.35 | 3.88E-03 |
| A_24_P82358 | Homo sapiens forkhead box C2 (MFH-1, mesenchyme forkhead 1) (FOXC2), mRNA [NM_005251] | 1.34 | 2.94E-02 |
| A_23_P202978 | Homo sapiens caspase 1, apoptosis-related cysteine protease (interleukin 1, beta, convertase) (CASP1), transcript variant alpha, mRNA [NM_033292] | 1.34 | 4.01E-03 |
| A_24_P316454 | Homo sapiens cDNA clone IMAGE:5441030, partial cds. [BC022826] | 1.34 | 2.48E-02 |
| A_23_P428184 | Homo sapiens histone 1, H2ad (HIST1H2AD), mRNA [NM_021065] | 1.34 | 5.65E-03 |
| A_24_P37903 |  | 1.34 | 1.21E-02 |
| A_23_P346309 | Homo sapiens BCL2-associated X protein (BAX), transcript variant gamma, mRNA [NM_138762] | 1.33 | 3.65E-02 |
| A_23_P30813 | Homo sapiens histone 1, H4k (HIST1H4K), mRNA [NM_003541] | 1.33 | 7.83E-03 |
| A_23_P156609 |  | 1.33 | 1.60E-03 |
| A_23_P415984 | Homo sapiens neuronal PAS domain protein 2 (NPAS2), mRNA [NM_002518] | 1.33 | 3.17E-02 |
| A_23_P160318 | Homo sapiens collagen, type XVI, alpha 1 (COL16A1), mRNA [NM_001856] | 1.33 | 2.01E-02 |
| A_23_P61371 | Homo sapiens hypothetical protein LOC340061 (LOC340061), mRNA [NM_198282] | 1.33 | 8.83E-03 |
| A_23_P435029 | Homo sapiens, histone gene complex 1, clone MGC:9629 IMAGE:3913365, mRNA, complete cds. [BC015544] | 1.33 | 1.01E-03 |
| A_24_P403016 | Homo sapiens cDNA: FLJ21477 fis, clone COL04982. [AK025130] | 1.32 | 3.32E-02 |
| A_23_P421306 | Homo sapiens synaptotagmin XII (SYT12), mRNA [NM_177963] | 1.32 | 6.77E-03 |
| A_23_P256470 | Homo sapiens neuropeptide Y (NPY), mRNA [NM_000905] | 1.32 | 6.64E-03 |
| A_23_P35148 | Homo sapiens TAF13 RNA polymerase II, TATA box binding protein (TBP)-associated factor, 18kDa (TAF13), mRNA [NM_005645] | 1.31 | 3.87E-03 |
| A_23_P30799 | Homo sapiens histone 1, H3f (HIST1H3F), mRNA [NM_021018] | 1.31 | 4.10E-02 |
| A_32_P221799 | Homo sapiens histone 1, H2am (HIST1H2AM), mRNA [NM_003514] | 1.30 | 2.88E-02 |
| A_23_P362719 | Homo sapiens cDNA clone MGC:61931 IMAGE:6565452, complete cds. [BC054888] | 1.30 | 3.10E-02 |
| A_32_P108420 |  | 1.30 | 3.61E-03 |
| A_23_P93258 | Homo sapiens histone 1, H3b (HIST1H3B), mRNA [NM_003537] | 1.29 | 1.63E-02 |
| A_32_P820503 | Homo sapiens ferritin, heavy polypeptide 1 (FTH1), mRNA [NM_002032] | 1.29 | 4.29E-02 |
| A_23_P139786 | Homo sapiens 2'-5'-oligoadenylate synthetase-like (OASL), transcript variant 1, mRNA [NM_003733] | 1.29 | 1.52E-02 |
| A_23_P401106 | Homo sapiens phosphodiesterase 2A, cGMP-stimulated (PDE2A), mRNA [NM_002599] | 1.29 | 3.24E-02 |
| A_23_P251002 |  | 1.29 | 3.47E-02 |
| A_23_P362415 | Homo sapiens ubiquitin-conjugating enzyme E2B (RAD6 homolog), mRNA (cDNA clone IMAGE:2967519), partial cds. [BC001694] | 1.29 | 2.00E-02 |
| A_23_P200801 | Homo sapiens phosphodiesterase 4D interacting protein (myomegalin) (PDE4DIP), transcript variant 5, mRNA [NM_001002811] | 1.29 | 1.04E-02 |
| A_24_P412486 | full-length cDNA clone CS0DC001YJ02 of Neuroblastoma Cot 25-normalized of Homo sapiens (human). [CR601315] | 1.29 | 4.27E-02 |
| A_32_P53633 | full-length cDNA clone CS0DI009YA14 of Placenta Cot 25-normalized of Homo sapiens (human). [CR613972] | 1.29 | 1.69E-02 |
| A_23_P82169 | Homo sapiens SRY (sex determining region Y)-box 4 (SOX4), mRNA [NM_003107] | 1.29 | 1.20E-02 |
| A_24_P283189 | Homo sapiens CD14 antigen (CD14), mRNA [NM_000591] | 1.28 | 1.15E-02 |
| A_23_P315364 | Homo sapiens chemokine (C-X-C motif) ligand 2 (CXCL2), mRNA [NM_002089] | 1.28 | 5.24E-03 |
| A_23_P107775 | Homo sapiens MDAC1 (MDAC1), mRNA [NM_139172] | 1.28 | 3.34E-02 |
| A_24_P147461 | Homo sapiens serine (or cysteine) proteinase inhibitor, clade B (ovalbumin), member 8 (SERPINB8), transcript variant 2, mRNA [NM_198833] | 1.27 | 4.12E-02 |
| A_24_P28722 | Homo sapiens radical S-adenosyl methionine domain containing 2 (RSAD2), mRNA [NM_080657] | 1.27 | 6.02E-03 |
| A_23_P71037 | Homo sapiens interleukin 6 (interferon, beta 2) (IL6), mRNA [NM_000600] | 1.27 | 2.05E-02 |
| A_23_P404494 | Homo sapiens interleukin 7 receptor (IL7R), mRNA [NM_002185] | 1.27 | 3.33E-02 |
| A_32_P46544 |  | 1.27 | 4.90E-02 |
| A_23_P351275 | Homo sapiens uridine phosphorylase 1 (UPP1), transcript variant 2, mRNA [NM_181597] | 1.27 | 1.56E-02 |
| A_23_P50919 | Homo sapiens serine (or cysteine) proteinase inhibitor, clade E (nexin, plasminogen activator inhibitor type 1), member 2 (SERPINE2), mRNA [NM_006216] | 1.27 | 5.37E-04 |
| A_23_P160559 | Homo sapiens extracellular matrix protein 1 (ECM1), transcript variant 1, mRNA [NM_004425] | 1.27 | 1.84E-02 |
| A_23_P147805 | Homo sapiens uridine phosphorylase 1, mRNA (cDNA clone MGC:54255 IMAGE:5549432), complete cds. [BC047030] | 1.27 | 4.78E-02 |
| A_24_P324396 | Homo sapiens HSPC088 mRNA, partial cds. [AF161351] | 1.27 | 4.47E-02 |
| A_24_P381199 | Homo sapiens tripartite motif-containing 6 (TRIM6), transcript variant 1, mRNA [NM_001003818] | 1.26 | 4.38E-02 |
| A_24_P251764 | Homo sapiens chemokine (C-X-C motif) ligand 3 (CXCL3), mRNA [NM_002090] | 1.26 | 8.59E-03 |
| A_23_P149301 | Homo sapiens histone 3, H2a (HIST3H2A), mRNA [NM_033445] | 1.26 | 2.54E-03 |
| A_23_P122216 | Homo sapiens lysyl oxidase (LOX), mRNA [NM_002317] | 1.26 | 1.08E-02 |
| A_23_P24004 | Homo sapiens interferon-induced protein with tetratricopeptide repeats 2 (IFIT2), mRNA [NM_001547] | 1.26 | 8.80E-03 |
| A_23_P35309 | Homo sapiens TAF5-like RNA polymerase II, p300/CBP-associated factor (PCAF)-associated factor, 65kDa (TAF5L), transcript variant 1, mRNA [NM_014409] | 1.26 | 3.53E-02 |
| A_24_P157926 | Homo sapiens tumor necrosis factor, alpha-induced protein 3 (TNFAIP3), mRNA [NM_006290] | 1.25 | 2.30E-02 |
| A_24_P277657 | Homo sapiens guanosine monophosphate reductase (GMPR), mRNA [NM_006877] | 1.25 | 3.50E-02 |
| A_23_P162589 | Homo sapiens vitamin D (1,25- dihydroxyvitamin D3) receptor (VDR), transcript variant 2, mRNA [NM_001017535] | 1.25 | 4.17E-02 |
| A_23_P327156 | Homo sapiens cDNA FLJ37574 fis, clone BRCOC2003100. [AK094893] | 1.25 | 1.98E-02 |
| A_32_P165557 |  | 1.25 | 4.00E-02 |
| A_23_P59045 | Homo sapiens histone 1, H2ae (HIST1H2AE), mRNA [NM_021052] | 1.25 | 3.27E-04 |
| A_32_P18440 | Homo sapiens mRNA; cDNA DKFZp686G23148 (from clone DKFZp686G23148). [BX641020] | 1.25 | 1.17E-03 |
| A_23_P110167 | Homo sapiens microsomal glutathione S-transferase 2 (MGST2), mRNA [NM_002413] | 1.25 | 4.77E-02 |
| A_23_P420942 | Homo sapiens metallothionein 1E (functional) (MT1E), mRNA [NM_175617] | 1.25 | 4.98E-02 |
| A_32_P101031 | Homo sapiens LY6/PLAUR domain containing 1 (LYPDC1), mRNA [NM_144586] | 1.25 | 3.30E-02 |
| A_23_P358597 | Homo sapiens popeye domain containing 3 (POPDC3), mRNA [NM_022361] | 1.24 | 9.81E-03 |
| A_23_P151506 | Homo sapiens pleckstrin 2 (PLEK2), mRNA [NM_016445] | 1.24 | 2.65E-03 |
| A_24_P367473 | Homo sapiens chemokine (C-C motif) receptor 3 (CCR3), transcript variant 1, mRNA [NM_001837] | 1.24 | 1.36E-02 |
| A_23_P111041 | Homo sapiens histone 1, H2bi (HIST1H2BI), mRNA [NM_003525] | 1.24 | 3.36E-03 |
| A_23_P35912 | Homo sapiens caspase 4, apoptosis-related cysteine protease (CASP4), transcript variant gamma, mRNA [NM_033306] | 1.24 | 2.11E-02 |
| A_32_P41461 | Homo sapiens WAS protein family, member 2 (WASF2), mRNA [NM_006990] | 1.24 | 3.87E-02 |
| A_32_P170368 | AI878825 au50b09.y1 Schneider fetal brain 00004 Homo sapiens cDNA clone IMAGE:2518169 5', mRNA sequence [AI878825] | 1.24 | 4.99E-02 |
| A_24_P11462 | Homo sapiens arginine decarboxylase (ADC), mRNA [NM_052998] | 1.24 | 1.65E-02 |
| A_24_P511686 | full-length cDNA clone CS0DF020YJ04 of Fetal brain of Homo sapiens (human). [CR616845] | 1.24 | 3.04E-02 |
| A_23_P54781 | Homo sapiens retinoblastoma binding protein 6, mRNA (cDNA clone IMAGE:6214974), complete cds. [BC051317] | 1.23 | 5.38E-03 |
| A_24_P318656 | Homo sapiens integrin, beta 3 (platelet glycoprotein IIIa, antigen CD61) (ITGB3), mRNA [NM_000212] | 1.23 | 1.52E-02 |
| A_23_P87150 | Homo sapiens leupaxin (LPXN), mRNA [NM_004811] | 1.23 | 8.56E-03 |
| A_23_P76529 | Homo sapiens integrin, beta 7 (ITGB7), mRNA [NM_000889] | 1.23 | 1.78E-02 |
| A_24_P810290 | Homo sapiens cDNA FLJ25802 fis, clone TST07145. [AK098668] | 1.23 | 5.75E-03 |
| A_24_P406334 | Homo sapiens six transmembrane epithelial antigen of the prostate 1 (STEAP1), mRNA [NM_012449] | 1.23 | 1.21E-02 |
| A_23_P13740 | Homo sapiens neuron navigator 3 (NAV3), mRNA [NM_014903] | 1.23 | 7.00E-03 |
| A_24_P51061 | Homo sapiens discoidin, CUB and LCCL domain containing 2 (DCBLD2), mRNA [NM_080927] | 1.23 | 4.45E-02 |
| A_32_P171793 |  | 1.23 | 2.20E-02 |
| A_24_P217834 | Homo sapiens histone 1, H3d (HIST1H3D), mRNA [NM_003530] | 1.23 | 3.04E-02 |
| A_23_P111701 | Homo sapiens guanine nucleotide binding protein (G protein), gamma 11 (GNG11), mRNA [NM_004126] | 1.22 | 2.98E-02 |
| A_24_P699896 | Homo sapiens cDNA clone IMAGE:5296862. [BC036637] | 1.22 | 2.68E-03 |
| A_23_P436281 | Homo sapiens histone 2, H4 (HIST2H4), mRNA [NM_003548] | 1.22 | 7.65E-03 |
| A_24_P11384 | Homo sapiens mitogen-inducible gene 6 (MIG-6), mRNA [NM_018948] | 1.22 | 4.95E-02 |
| A_32_P16204 | Homo sapiens hypothetical gene supported by BC013438, mRNA (cDNA clone IMAGE:3899073), partial cds. [BC013438] | 1.21 | 2.27E-03 |
| A_23_P323685 | Homo sapiens histone 1, H4h (HIST1H4H), mRNA [NM_003543] | 1.21 | 1.52E-02 |
| A_24_P115762 | Homo sapiens cathepsin C (CTSC), transcript variant 2, mRNA [NM_148170] | 1.21 | 5.79E-03 |
| A_23_P136721 | Human endogenous retrovirus H protease/integrase-derived ORF1, ORF2, and putative envelope protein mRNA, complete cds. [U88896] | 1.21 | 2.94E-02 |
| A_23_P5435 | Homo sapiens clone DNA129535 MRV222 (UNQ3066) mRNA, complete cds. [AY358993] | 1.21 | 2.70E-02 |
| A_23_P16722 | Homo sapiens dedicator of cytokinesis 10 (DOCK10), mRNA [NM_014689] | 1.21 | 1.20E-02 |
| A_24_P146138 | Homo sapiens protocadherin alpha 1 (PCDHA1), transcript variant 2, mRNA [NM_031410] | 1.21 | 4.44E-02 |
| A_23_P163251 | Homo sapiens progestin and adipoQ receptor family member V (PAQR5), mRNA [NM_017705] | 1.21 | 4.07E-05 |
| A_24_P175188 | Homo sapiens sterile alpha motif domain containing 9 (SAMD9), mRNA [NM_017654] | 1.21 | 3.24E-02 |
| A_23_P84849 | Human N-type calcium channel alpha-1 subunit mRNA, complete cds. [M94173] | 1.21 | 3.87E-02 |
| A_24_P68631 | Homo sapiens histone 2, H2ab (HIST2H2AB), mRNA [NM_175065] | 1.21 | 1.84E-02 |
| A_24_P55148 | Homo sapiens histone 1, H2bj (HIST1H2BJ), mRNA [NM_021058] | 1.21 | 1.31E-02 |
| A_24_P294233 | Homo sapiens glutaminase (GLS), mRNA [NM_014905] | 1.21 | 1.35E-02 |
| A_24_P20873 | Homo sapiens histone 1, H4i (HIST1H4I), mRNA [NM_003495] | 1.21 | 3.43E-02 |
| A_23_P120606 |  | 1.21 | 3.46E-02 |
| A_24_P127051 |  | 1.21 | 1.57E-03 |
| A_32_P37592 | Q7Z5X4 (Q7Z5X4) Intermediate filament-like protein MGC:2625, isoform 1, partial (12%) [THC2301370] | 1.21 | 2.37E-02 |
| A_24_P544661 | Homo sapiens cDNA clone IMAGE:6380649, containing frame-shift errors. [BC068044] | 1.21 | 2.07E-03 |
| A_23_P108948 | Homo sapiens dilute suppressor (DSU), mRNA [NM_018000] | 1.21 | 4.16E-02 |
| A_32_P153469 | AA298150 EST113740 Bone VII Homo sapiens cDNA 5' end, mRNA sequence [AA298150] | 1.21 | 3.78E-02 |
| A_23_P351556 | full-length cDNA clone CS0DF004YO04 of Fetal brain of Homo sapiens (human). [CR594281] | 1.20 | 4.47E-02 |
| A_23_P135568 | TNIK_HUMAN (Q9UKE5) TRAF2 and NCK interacting kinase, complete [THC2398745] | 1.20 | 8.22E-03 |
| A_23_P167997 | Homo sapiens histone 1, H2bg (HIST1H2BG), mRNA [NM_003518] | 1.20 | 1.70E-02 |
| A_23_P123022 | Homo sapiens tyrosine 3-monooxygenase/tryptophan 5-monooxygenase activation protein, gamma polypeptide (YWHAG), mRNA [NM_012479] | 1.20 | 4.10E-02 |
| A_23_P71241 | Homo sapiens Sec61 gamma subunit (SEC61G), transcript variant 1, mRNA [NM_014302] | 1.20 | 1.54E-02 |
| A_24_P156911 | Homo sapiens histone 2, H2be (HIST2H2BE), mRNA [NM_003528] | 1.20 | 1.59E-02 |
| A_24_P146211 | Homo sapiens histone 1, H2bd (HIST1H2BD), transcript variant 1, mRNA [NM_021063] | 1.20 | 2.03E-02 |
| A_24_P733083 |  | 1.20 | 3.04E-02 |
| A_24_P196117 | Homo sapiens general transcription factor IIH, polypeptide 5 (GTF2H5), mRNA [NM_207118] | 1.20 | 4.72E-02 |
| A_32_P103220 | Homo sapiens a disintegrin-like and metalloprotease (reprolysin type) with thrombospondin type 1 motif, 12 (ADAMTS12), mRNA [NM_030955] | 1.20 | 4.04E-02 |
| A_32_P231265 | AI694800 wd62c03.x1 NCI_CGAP_Lu24 Homo sapiens cDNA clone IMAGE:2336164 3', mRNA sequence [AI694800] | 1.20 | 2.65E-02 |
| A_23_P20122 | Homo sapiens zinc finger CCCH-type, antiviral 1 (ZC3HAV1), transcript variant 2, mRNA [NM_024625] | 1.20 | 3.90E-02 |
| A_23_P127394 | Homo sapiens cryptochrome 2 (photolyase-like) (CRY2), mRNA [NM_021117] | 1.20 | 1.71E-02 |
| A_23_P102117 | Homo sapiens wingless-type MMTV integration site family, member 10A (WNT10A), mRNA [NM_025216] | 1.19 | 1.63E-02 |
| A_24_P355649 | Homo sapiens Friend leukemia virus integration 1 (FLI1), mRNA [NM_002017] | 1.19 | 3.31E-02 |
| A_32_P67533 | Homo sapiens l(3)mbt-like 3 (Drosophila) (L3MBTL3), transcript variant 1, mRNA [NM_032438] | 1.19 | 4.56E-04 |
| A_24_P416346 | Homo sapiens ets variant gene 4 (E1A enhancer binding protein, E1AF) (ETV4), mRNA [NM_001986] | 1.19 | 1.54E-03 |
| A_23_P143285 | Homo sapiens cDNA FLJ20802 fis, clone ADSU01223. [AK000809] | 1.19 | 4.08E-02 |
| A_23_P154037 | Homo sapiens aldehyde oxidase 1 (AOX1), mRNA [NM_001159] | 1.19 | 7.04E-03 |
| A_32_P20535 | AL527314 Homo sapiens NEUROBLASTOMA COT 25-NORMALIZED Homo sapiens cDNA clone CS0DC021YG08 3-PRIME, mRNA sequence [AL527314] | 1.19 | 3.68E-02 |
| A_23_P256205 | Homo sapiens actin binding LIM protein family, member 3 (ABLIM3), mRNA [NM_014945] | 1.19 | 1.36E-03 |
| A_23_P42178 | Homo sapiens histone 1, H2bf (HIST1H2BF), mRNA [NM_003522] | 1.19 | 1.44E-02 |
| A_23_P153022 | Homo sapiens keratin associated protein 2-4 (KRTAP2-4), mRNA [NM_033184] | 1.19 | 4.56E-02 |
| A_32_P15874 | Q8INN3 (Q8INN3) CG31415-PA, partial (7%) [THC2383106] | 1.19 | 1.48E-03 |
| A_23_P79622 | Homo sapiens FK506 binding protein 7 (FKBP7), transcript variant 1, mRNA [NM_016105] | 1.19 | 2.60E-02 |
| A_24_P144383 |  | 1.19 | 2.97E-02 |
| A_23_P74088 | Homo sapiens matrix metalloproteinase 23B (MMP23B), mRNA [NM_006983] | 1.19 | 3.62E-02 |
| A_23_P374689 | Homo sapiens glutamate decarboxylase 1 (brain, 67kDa) (GAD1), transcript variant GAD67, mRNA [NM_000817] | 1.19 | 7.24E-03 |
| A_23_P130089 | Homo sapiens intraflagellar transport protein IFT20 (IFT20), mRNA [NM_174887] | 1.19 | 2.19E-02 |
| A_23_P1962 | Homo sapiens retinoic acid receptor responder (tazarotene induced) 3 (RARRES3), mRNA [NM_004585] | 1.19 | 2.65E-02 |
| A_23_P167983 | Homo sapiens histone 1, H2ac, mRNA (cDNA clone MGC:1730 IMAGE:2988620), complete cds. [BC017379] | 1.18 | 3.58E-02 |
| A_24_P178175 | Homo sapiens gamma-glutamyltransferase 2 (GGT2), mRNA [NM_002058] | 1.18 | 3.74E-02 |
| A_23_P350574 | Homo sapiens Fc receptor-like and mucin-like 2 (FCRLM2), mRNA [NM_152378] | 1.18 | 2.22E-02 |
| A_23_P402081 | Homo sapiens histone 1, H2bn (HIST1H2BN), mRNA [NM_003520] | 1.18 | 2.49E-02 |
| A_24_P110141 | Homo sapiens hypothetical protein DKFZp434I1020 (DKFZp434I1020), mRNA [NM_194295] | 1.18 | 2.09E-02 |
| A_24_P381455 | Homo sapiens hypothetical protein FLJ11259, mRNA (cDNA clone MGC:21716 IMAGE:4474297), complete cds. [BC018435] | 1.18 | 3.26E-03 |
| A_32_P118568 | Homo sapiens RET finger protein-like 1 antisense transcript, partial. [AJ010230] | 1.18 | 4.91E-02 |
| A_23_P360804 | Homo sapiens copine V (CPNE5), mRNA [NM_020939] | 1.18 | 7.40E-03 |
| A_24_P255663 |  | 1.18 | 3.16E-02 |
| A_24_P394510 | Homo sapiens histone 1, H2aj (HIST1H2AJ), mRNA [NM_021066] | 1.18 | 1.85E-02 |
| A_23_P2271 | Homo sapiens parathyroid hormone-like hormone (PTHLH), transcript variant 1, mRNA [NM_198965] | 1.18 | 1.45E-02 |
| A_23_P403521 | Homo sapiens chromosome 7 open reading frame 36 (C7orf36), mRNA [NM_020192] | 1.18 | 3.58E-03 |
| A_23_P53193 | Homo sapiens synaptotagmin-like 2 (SYTL2), transcript variant c, mRNA [NM_206927] | 1.18 | 1.29E-02 |
| A_23_P309991 | Homo sapiens BCL2-like 11 (apoptosis facilitator) (BCL2L11), transcript variant 2, mRNA [NM_138622] | 1.18 | 1.52E-02 |
| A_23_P344531 | Homo sapiens mRNA for KIAA1029 protein, partial cds. [AB028952] | 1.18 | 1.27E-02 |
| A_32_P69149 | Homo sapiens six transmembrane epithelial antigen of the prostate 1 (STEAP1), mRNA [NM_012449] | 1.18 | 1.45E-02 |
| A_32_P157504 | Homo sapiens cDNA FLJ37310 fis, clone BRAMY2016706. [AK094629] | 1.18 | 1.60E-02 |
| A_24_P659122 | Homo sapiens hypothetical LOC401357 (LOC401357), mRNA [NM_001013685] | 1.18 | 3.78E-02 |
| A_32_P119736 | O63611 (O63611) NADH dehydrogenase subunit 2, partial (5%) [THC2386560] | 1.18 | 3.60E-02 |
| A_24_P585004 |  | 1.18 | 1.83E-03 |
| A_23_P255076 | Homo sapiens RWD domain containing 2 (RWDD2), mRNA [NM_033411] | 1.18 | 3.52E-02 |
| A_23_P105012 | Homo sapiens HRAS-like suppressor 2 (HRASLS2), mRNA [NM_017878] | 1.17 | 1.27E-02 |
| A_32_P62211 | Homo sapiens mRNA; cDNA DKFZp686J1595 (from clone DKFZp686J1595) [BX538057] | 1.17 | 2.64E-02 |
| A_23_P502520 | Homo sapiens interleukin 4 induced 1 (IL4I1), transcript variant 2, mRNA [NM_172374] | 1.17 | 3.26E-02 |
| A_23_P330788 | Homo sapiens IQ motif and Sec7 domain 2 (IQSEC2), mRNA [NM_015075] | 1.17 | 3.41E-02 |
| A_23_P40470 | Homo sapiens H2B histone family, member S (H2BFS), mRNA [NM_017445] | 1.17 | 2.50E-02 |
| A_23_P51856 | Homo sapiens dual specificity phosphatase 10 (DUSP10), transcript variant 1, mRNA [NM_007207] | 1.17 | 1.75E-02 |
| A_32_P162250 | Homo sapiens Rho GTPase activating protein 18 (ARHGAP18), mRNA [NM_033515] | 1.17 | 2.46E-02 |
| A_23_P23346 | Homo sapiens myeloid/lymphoid or mixed-lineage leukemia (trithorax homolog, Drosophila); translocated to, 11 (MLLT11), mRNA [NM_006818] | 1.17 | 1.78E-02 |
| A_23_P333484 | Homo sapiens histone 1, H3h (HIST1H3H), mRNA [NM_003536] | 1.17 | 1.81E-02 |
| A_23_P104346 | Homo sapiens phosphatidylinositol-4-phosphate 5-kinase, type II, alpha (PIP5K2A), mRNA [NM_005028] | 1.17 | 3.67E-02 |
| A_23_P111797 | Homo sapiens mRNA; cDNA DKFZp434F142 (from clone DKFZp434F142). [AL136837] | 1.17 | 2.87E-02 |
| A_32_P319200 | Homo sapiens gamma-glutamyltransferase-like 4 (GGTL4), transcript variant 1, mRNA [NM_199127] | 1.17 | 3.91E-02 |
| A_23_P201459 | Homo sapiens interferon, alpha-inducible protein (clone IFI-6-16) (G1P3), transcript variant 3, mRNA [NM_022873] | 1.17 | 7.95E-03 |
| A_23_P406616 | Homo sapiens hypothetical protein FLJ36031 (FLJ36031), mRNA [NM_175884] | 1.17 | 2.32E-02 |
| A_24_P233078 | Homo sapiens peptide YY, 2 (seminalplasmin) (PYY2), mRNA [NM_021093] | 1.17 | 7.65E-04 |
| A_24_P661641 | Homo sapiens hypothetical gene supported by BC047417, mRNA (cDNA clone IMAGE:5288894). [BC047417] | 1.17 | 2.31E-02 |
| A_23_P117912 | full-length cDNA clone CS0DI031YH01 of Placenta Cot 25-normalized of Homo sapiens (human). [CR618466] | 1.17 | 4.16E-02 |
| A_24_P230877 | Homo sapiens, clone IMAGE:3606519, mRNA, partial cds. [BC009463] | 1.17 | 3.62E-02 |
| A_23_P500381 | Homo sapiens 5-hydroxytryptamine (serotonin) receptor 7 (adenylate cyclase-coupled) (HTR7), transcript variant d, mRNA [NM_019859] | 1.17 | 4.77E-02 |
| A_23_P500861 | Homo sapiens spectrin repeat containing, nuclear envelope 1 (SYNE1), transcript variant longest, mRNA [NM_182961] | 1.16 | 4.39E-02 |
| A_23_P424561 | Homo sapiens ras homolog gene family, member V (RHOV), mRNA [NM_133639] | 1.16 | 4.69E-03 |
| A_23_P332992 | Homo sapiens histone 3, H2bb (HIST3H2BB), mRNA [NM_175055] | 1.16 | 2.44E-02 |
| A_24_P220485 | Homo sapiens olfactomedin-like 2A (OLFML2A), mRNA [NM_182487] | 1.16 | 4.11E-02 |
| A_23_P93180 | Homo sapiens histone 1, H2bc (HIST1H2BC), mRNA [NM_003526] | 1.16 | 3.08E-02 |
| A_23_P218597 | Homo sapiens neuronal PAS domain protein 2 (NPAS2), mRNA [NM_002518] | 1.16 | 8.00E-03 |
| A_24_P479645 | Homo sapiens cDNA FLJ36321 fis, clone THYMU2005482. [AK093640] | 1.16 | 3.72E-02 |
| A_24_P57977 | Homo sapiens SNAP25-interacting protein (SNIP), mRNA [NM_025248] | 1.16 | 2.90E-02 |
| A_23_P205531 | Homo sapiens ribonuclease, RNase A family, 4 (RNASE4), transcript variant 1, mRNA [NM_194430] | 1.16 | 1.73E-02 |
| A_23_P71989 | Homo sapiens uridine phosphorylase 1 (UPP1), transcript variant 2, mRNA [NM_181597] | 1.16 | 4.25E-02 |
| A_23_P137856 | Homo sapiens mucin 1, transmembrane (MUC1), transcript variant 1, mRNA [NM_002456] | 1.16 | 1.46E-02 |
| A_23_P147918 | Homo sapiens S100 calcium binding protein A16 (S100A16), mRNA [NM_080388] | 1.16 | 3.93E-02 |
| A_32_P94685 | Homo sapiens, clone IMAGE:4819084, mRNA. [BC042589] | 1.16 | 3.05E-03 |
| A_32_P5628 |  | 1.16 | 2.48E-02 |
| A_24_P41918 | Homo sapiens, clone IMAGE:3685861, mRNA. [BC030714] | 1.16 | 3.81E-02 |
| A_24_P788772 | APE_HUMAN (P02649) Apolipoprotein E precursor (Apo-E), partial (50%) [THC2373524] | 1.16 | 3.72E-02 |
| A_23_P134347 | Homo sapiens carboxypeptidase, vitellogenic-like (CPVL), transcript variant 1, mRNA [NM_031311] | 1.16 | 1.84E-02 |
| A_23_P134935 | Homo sapiens dual specificity phosphatase 4 (DUSP4), transcript variant 1, mRNA [NM_001394] | 1.16 | 2.91E-03 |
| A_24_P178415 |  | 1.16 | 4.97E-02 |
| A_23_P29769 | Homo sapiens WW domain containing transcription regulator 1 (WWTR1), mRNA [NM_015472] | 1.16 | 4.68E-02 |
| A_24_P252739 | Homo sapiens Kruppel-like factor 6 (KLF6), transcript variant 1, mRNA [NM_001008490] | 1.16 | 2.66E-02 |
| A_23_P205074 | Homo sapiens hypothetical protein LOC283537 (LOC283537), mRNA [NM_181785] | 1.16 | 4.47E-02 |
| A_23_P58763 | Homo sapiens pelota homolog (Drosophila) (PELO), mRNA [NM_015946] | 1.16 | 1.92E-02 |
| A_23_P37914 | Homo sapiens solute carrier family 5 (sodium/glucose cotransporter), member 11 (SLC5A11), mRNA [NM_052944] | 1.16 | 1.25E-02 |
| A_23_P418934 | Homo sapiens similar to RIKEN cDNA 8030451K01 (LOC387921), transcript variant 2, mRNA [NM_001017370] | 1.16 | 3.59E-02 |
| A_23_P83007 | Homo sapiens chromosome 9 open reading frame 150 (C9orf150), mRNA [NM_203403] | 1.15 | 9.34E-03 |
| A_23_P7402 | Homo sapiens PDZ domain containing 3 (PDZK3), transcript variant 1, mRNA [NM_178140] | 1.15 | 6.81E-03 |
| A_24_P252078 | Homo sapiens cDNA clone MGC:71335 IMAGE:6088873, complete cds. [BC067086] | 1.15 | 3.41E-02 |
| A_32_P199801 | Homo sapiens solute carrier family 2 (facilitated glucose transporter), member 13 (SLC2A13), mRNA [NM_052885] | 1.15 | 1.65E-02 |
| A_23_P129334 | Homo sapiens chloride channel 7 (CLCN7), mRNA [NM_001287] | 1.15 | 7.81E-03 |
| A_23_P136573 | Homo sapiens ST3 beta-galactoside alpha-2,3-sialyltransferase 5 (ST3GAL5), mRNA [NM_003896] | 1.15 | 8.93E-03 |
| A_32_P53311 | Homo sapiens cDNA FLJ44257 fis, clone TKIDN2015263. [AK126245] | 1.15 | 2.00E-02 |
| A_24_P101282 | Homo sapiens, clone IMAGE:5019307, mRNA. [BC031342] | 1.15 | 3.64E-02 |
| A_24_P917123 | Homo sapiens myosin regulatory light chain interacting protein (MYLIP), mRNA [NM_013262] | 1.15 | 2.63E-03 |
| A_24_P818010 | Homo sapiens cDNA FLJ39761 fis, clone SPLEN1000083. [AK097080] | 1.15 | 1.86E-02 |
| A_24_P535483 | Homo sapiens hypothetical protein LOC284739 (LOC284739), mRNA [NM_207349] | 1.15 | 1.43E-02 |
| A_32_P228348 | Homo sapiens FLJ45248 protein (FLJ45248), mRNA [NM_207505] | 1.15 | 3.82E-02 |
| A_23_P105264 | Homo sapiens ets variant gene 6 (TEL oncogene) (ETV6), mRNA [NM_001987] | 1.15 | 3.79E-02 |
| A_23_P365738 | Homo sapiens activity-regulated cytoskeleton-associated protein (ARC), mRNA [NM_015193] | 1.15 | 3.49E-02 |
| A_23_P429383 | Homo sapiens homeo box D9 (HOXD9), mRNA [NM_014213] | 1.15 | 3.61E-02 |
| A_23_P155229 | Homo sapiens signal sequence receptor, gamma (translocon-associated protein gamma) (SSR3), mRNA [NM_007107] | 1.15 | 2.14E-02 |
| A_23_P1014 | Homo sapiens chromosome 1 open reading frame 97 (C1orf97), mRNA [NM_032705] | 1.15 | 2.77E-02 |
| A_24_P8721 | Homo sapiens histone 2, H2ac (HIST2H2AC), mRNA [NM_003517] | 1.15 | 3.10E-02 |
| A_32_P110390 | Homo sapiens proline-rich protein PRP2 (PRP2), mRNA [NM_173490] | 1.14 | 1.29E-02 |
| A_23_P46369 | Homo sapiens RAB13, member RAS oncogene family (RAB13), mRNA [NM_002870] | 1.14 | 2.07E-02 |
| A_23_P16609 | Homo sapiens mRNA; cDNA DKFZp761G18121 (from clone DKFZp761G18121). [AL136548] | 1.14 | 4.23E-02 |
| A_23_P214950 | Homo sapiens PERP, TP53 apoptosis effector (PERP), mRNA [NM_022121] | 1.14 | 3.28E-02 |
| A_23_P57856 | Homo sapiens B-cell CLL/lymphoma 6 (zinc finger protein 51) (BCL6), transcript variant 2, mRNA [NM_138931] | 1.14 | 9.11E-03 |
| A_23_P8013 | Homo sapiens histone 1, H2bl (HIST1H2BL), mRNA [NM_003519] | 1.14 | 2.38E-02 |
| A_23_P22765 | Homo sapiens NADH dehydrogenase (ubiquinone) 1 beta subcomplex, 11, 17.3kDa (NDUFB11), mRNA [NM_019056] | 1.14 | 3.55E-02 |
| A_23_P15394 | Homo sapiens CD68 antigen (CD68), mRNA [NM_001251] | 1.14 | 3.41E-02 |
| A_24_P101722 | PREDICTED: Homo sapiens similar to peptidyl-Pro cis trans isomerase (LOC126170), mRNA [XM_497621] | 1.14 | 4.39E-02 |
| A_23_P257423 | Homo sapiens hypothetical protein MGC19780 (MGC19780), mRNA [NM_144988] | 1.14 | 1.42E-02 |
| A_24_P124567 | Homo sapiens ORM1-like 2 (S. cerevisiae) (ORMDL2), mRNA [NM_014182] | 1.14 | 1.49E-02 |
| A_24_P390096 | Homo sapiens glioma pathogenesis-related protein (GliPR) mRNA, complete cds. [U16307] | 1.14 | 2.63E-02 |
| A_23_P77859 | Homo sapiens similar to RIKEN cDNA 2600017H02 (LOC92162), mRNA [NM_203411] | 1.14 | 2.56E-02 |
| A_32_P115606 | Homo sapiens cDNA FLJ16460 fis, clone BRCAN2018240. [AK131385] | 1.14 | 1.36E-02 |
| A_24_P942589 | Homo sapiens mRNA; cDNA DKFZp761G1111 (from clone DKFZp761G1111). [AL137342] | 1.14 | 2.98E-03 |
| A_23_P50368 | Homo sapiens osteoclast-associated receptor (OSCAR), transcript variant 1, mRNA [NM_206818] | 1.14 | 1.64E-03 |
| A_23_P316472 | Homo sapiens hypothetical protein FLJ32752 (FLJ32752), mRNA [NM_144666] | 1.14 | 2.87E-02 |
| A_24_P121642 | PREDICTED: Homo sapiens similar to C367G8.3 (novel protein similar to RPL23A (60S ribosomal protein L23A)) (LOC441743), mRNA [XM_497481] | 1.14 | 3.30E-02 |
| A_23_P250042 | Homo sapiens selenoprotein T (SELT), mRNA [NM_016275] | 1.14 | 3.19E-02 |
| A_23_P150350 | Homo sapiens chromosome 11 open reading frame 1 (C11orf1), mRNA [NM_022761] | 1.14 | 1.17E-02 |
| A_23_P134953 | Homo sapiens adipose differentiation-related protein (ADFP), mRNA [NM_001122] | 1.14 | 1.64E-02 |
| A_23_P17074 | Homo sapiens hypothetical protein MGC12981 (MGC12981), mRNA [NM_032357] | 1.14 | 3.44E-04 |
| A_23_P104073 | Homo sapiens S100 calcium binding protein A3 (S100A3), mRNA [NM_002960] | 1.14 | 2.74E-02 |
| A_32_P86705 | Homo sapiens, clone IMAGE:5267797, mRNA. [BC040577] | 1.14 | 2.25E-03 |
| A_24_P58727 |  | 1.14 | 4.21E-02 |
| A_23_P145 | Homo sapiens 3-hydroxymethyl-3-methylglutaryl-Coenzyme A lyase (hydroxymethylglutaricaciduria) (HMGCL), mRNA [NM_000191] | 1.13 | 2.70E-02 |
| A_23_P59069 | Homo sapiens histone 1, H2bo (HIST1H2BO), mRNA [NM_003527] | 1.13 | 4.03E-02 |
| A_23_P103756 | Homo sapiens oviductal glycoprotein 1, 120kDa (mucin 9, oviductin) (OVGP1), mRNA [NM_002557] | 1.13 | 4.80E-02 |
| A_24_P149314 | Homo sapiens UL16 binding protein 2 (ULBP2), mRNA [NM_025217] | 1.13 | 4.11E-02 |
| A_24_P54000 | Homo sapiens chromosome 1 open reading frame 71 (C1orf71), mRNA [NM_152609] | 1.13 | 3.41E-02 |
| A_23_P5757 | Homo sapiens CGI-121 protein (CGI-121), mRNA [NM_016058] | 1.13 | 4.61E-02 |
| A_23_P50498 | Homo sapiens ferritin, light polypeptide (FTL), mRNA [NM_000146] | 1.13 | 1.42E-02 |
| A_32_P175715 | MEG1_MOUSE (Q61845) Meiosis expressed protein 1, partial (48%) [THC2405198] | 1.13 | 4.46E-02 |
| A_23_P211047 | Homo sapiens BTB and CNC homology 1, basic leucine zipper transcription factor 1 (BACH1), transcript variant 1, mRNA [NM_206866] | 1.13 | 4.91E-02 |
| A_24_P161403 | RST9844 Athersys RAGE Library Homo sapiens cDNA, mRNA sequence [BG190769] | 1.13 | 2.93E-02 |
| A_24_P917783 | H.sapiens mRNA for an acute myeloid leukaemia protein (1793bp). [X90978] | 1.13 | 3.55E-03 |
| A_23_P49546 | Homo sapiens glutamate receptor, ionotropic, N-methyl D-aspartate 2C (GRIN2C), mRNA [NM_000835] | 1.13 | 3.39E-02 |
| A_23_P25163 | Homo sapiens mitochondrial ribosomal protein L42 (MRPL42), nuclear gene encoding mitochondrial protein, transcript variant 3, mRNA [NM_172178] | 1.13 | 2.36E-02 |
| A_23_P37778 | Homo sapiens formin homology 2 domain containing 1 (FHOD1), mRNA [NM_013241] | 1.13 | 5.98E-03 |
| A_24_P347566 | Homo sapiens talin 2 (TLN2), mRNA [NM_015059] | 1.13 | 3.01E-02 |
| A_23_P18579 | Homo sapiens pituitary tumor-transforming 2 (PTTG2), mRNA [NM_006607] | 1.13 | 7.06E-03 |
| A_23_P380181 | Homo sapiens LIM domain only 4 (LMO4), mRNA [NM_006769] | 1.13 | 1.88E-02 |
| A_23_P120809 | Homo sapiens gamma-glutamyltransferase-like 4 (GGTL4), transcript variant 2, mRNA [NM_080839] | 1.13 | 2.47E-02 |
| A_24_P341476 | AF139893 cyclophilin 18 [Oryctolagus cuniculus;], partial (84%) [THC2301753] | 1.13 | 4.71E-02 |
| A_32_P213637 | Homo sapiens cDNA FLJ35623 fis, clone SPLEN2010986. [AK092942] | 1.13 | 2.57E-02 |
| A_23_P170713 |  | 1.13 | 1.21E-02 |
| A_23_P145153 | Homo sapiens programmed cell death 2 (PDCD2), transcript variant 1, mRNA [NM_002598] | 1.13 | 4.35E-02 |
| A_23_P154986 | Homo sapiens gamma-glutamyltransferase 1 (GGT1), transcript variant 3, mRNA [NM_013430] | 1.13 | 1.00E-02 |
| A_23_P376088 | Homo sapiens Lck interacting transmembrane adaptor 1 (LIME1), mRNA [NM_017806] | 1.13 | 2.31E-02 |
| A_32_P53107 | full-length cDNA clone CS0DA002YO22 of Neuroblastoma of Homo sapiens (human). [CR609342] | 1.13 | 8.86E-03 |
| A_23_P158880 | Homo sapiens START domain containing 5 (STARD5), transcript variant 1, mRNA [NM_181900] | 1.13 | 2.37E-02 |
| A_32_P30760 | O39496 (O39496) Phosphoprotein, partial (6%) [THC2438559] | 1.12 | 4.77E-02 |
| A_23_P353085 | Homo sapiens hypothetical protein FLJ35119 (FLJ35119), mRNA [NM_175871] | 1.12 | 3.45E-02 |
| A_24_P399694 | Homo sapiens zinc finger, CCHC domain containing 3 (ZCCHC3), mRNA [NM_033089] | 1.12 | 4.41E-02 |
| A_23_P7684 | Homo sapiens cDNA FLJ16450 fis, clone BRAWH2010552. [AK131381] | 1.12 | 2.89E-02 |
| A_23_P65678 | Homo sapiens fibrillin 1 (Marfan syndrome) (FBN1), mRNA [NM_000138] | 1.12 | 1.47E-02 |
| A_23_P408108 | Homo sapiens mitochondrial transcription termination factor (MTERF), nuclear gene encoding mitochondrial protein, mRNA [NM_006980] | 1.12 | 1.02E-02 |
| A_32_P204376 | Homo sapiens OTTHUMP00000064580 (LOC441430), mRNA [NM_001012421] | 1.12 | 1.74E-02 |
| A_24_P323778 |  | 1.12 | 3.12E-02 |
| A_23_P1819 | Homo sapiens olfactory receptor, family 8, subfamily B, member 8 (OR8B8), mRNA [NM_012378] | 1.12 | 2.11E-02 |
| A_23_P138507 | Homo sapiens cell division cycle 2, G1 to S and G2 to M (CDC2), transcript variant 1, mRNA [NM_001786] | 1.12 | 6.25E-03 |
| A_23_P215484 | Homo sapiens chemokine (C-C motif) ligand 26 (CCL26), mRNA [NM_006072] | 1.12 | 3.62E-02 |
| A_32_P79966 |  | 1.12 | 3.79E-02 |
| A_24_P803885 | Homo sapiens hypothetical protein LOC149134 (LOC149134), mRNA [NM_207326] | 1.12 | 2.32E-02 |
| A_32_P33723 | Homo sapiens, clone IMAGE:5240818, mRNA. [BC028229] | 1.12 | 4.79E-02 |
| A_23_P96350 | Homo sapiens PRA1 domain family, member 2 (PRAF2), mRNA [NM_007213] | 1.12 | 4.55E-02 |
| A_23_P307346 | Homo sapiens carbonic anhydrase VB, mitochondrial (CA5B), nuclear gene encoding mitochondrial protein, mRNA [NM_007220] | 1.12 | 3.19E-03 |
| A_23_P33809 | Homo sapiens IMP3, U3 small nucleolar ribonucleoprotein, homolog (yeast) (IMP3), mRNA [NM_018285] | 1.12 | 2.74E-02 |
| A_24_P342591 | Homo sapiens arginine-glutamic acid dipeptide (RE) repeats (RERE), mRNA [NM_012102] | 1.12 | 6.50E-03 |
| A_24_P392231 | HSL31 ribosomal protein L31 [Homo sapiens;], complete [THC2360930] | 1.12 | 4.17E-02 |
| A_23_P330070 | Homo sapiens tissue factor pathway inhibitor (lipoprotein-associated coagulation inhibitor), mRNA (cDNA clone MGC:9251 IMAGE:3902987), complete cds. [BC015514] | 1.12 | 4.77E-02 |
| A_23_P27075 | Homo sapiens GABA(A) receptor-associated protein (GABARAP), mRNA [NM_007278] | 1.12 | 4.58E-02 |
| A_23_P66599 | Homo sapiens hypothetical protein MGC10540 (MGC10540), mRNA [NM_032353] | 1.12 | 2.71E-02 |
| A_23_P144369 | Homo sapiens nucleosome assembly protein 1-like 5 (NAP1L5), mRNA [NM_153757] | 1.12 | 1.81E-02 |
| A_24_P114617 | Homo sapiens chromatin modifying protein 2B (CHMP2B), mRNA [NM_014043] | 1.12 | 8.92E-03 |
| A_24_P761130 | Homo sapiens cDNA FLJ39761 fis, clone SPLEN1000083. [AK097080] | 1.12 | 1.70E-02 |
| A_23_P109677 |  | 1.12 | 4.48E-02 |
| A_32_P11230 | Homo sapiens hypothetical LOC399744 (LOC399744), mRNA [NM_001013665] | 1.12 | 1.97E-02 |
| A_24_P208595 | Homo sapiens anthrax toxin receptor 1 (ANTXR1), transcript variant 2, mRNA [NM_053034] | 1.12 | 3.05E-02 |
| A_23_P22682 | Homo sapiens armadillo repeat containing, X-linked 1 (ARMCX1), mRNA [NM_016608] | 1.12 | 6.63E-03 |
| A_23_P342348 | Homo sapiens cytochrome c oxidase subunit IV isoform 1, mRNA (cDNA clone IMAGE:5240622), complete cds. [BC047869] | 1.12 | 3.95E-02 |
| A_32_P10187 |  | 1.12 | 1.33E-02 |
| A_32_P32061 | Homo sapiens chromosome 2 open reading frame 27 (C2orf27), mRNA [NM_013310] | 1.11 | 2.78E-02 |
| A_23_P62351 | Homo sapiens armadillo repeat containing, X-linked 6 (ARMCX6), transcript variant 1, mRNA [NM_019007] | 1.11 | 1.23E-02 |
| A_23_P48717 | Homo sapiens Niemann-Pick disease, type C2 (NPC2), mRNA [NM_006432] | 1.11 | 4.74E-02 |
| A_23_P399112 | Homo sapiens myeloid-associated differentiation marker (MYADM), transcript variant 2, mRNA [NM_138373] | 1.11 | 5.74E-03 |
| A_23_P60016 | Homo sapiens pituitary tumor transforming gene protein 3 (PTTG3) mRNA, complete cds. [AF095289] | 1.11 | 2.58E-03 |
| A_23_P206284 | Homo sapiens G protein-coupled receptor 56 (GPR56), transcript variant 3, mRNA [NM_201525] | 1.11 | 2.82E-03 |
| A_23_P325119 | Homo sapiens hypothetical gene LOC128439 (LOC128439), mRNA [NM_139016] | 1.11 | 9.06E-03 |
| A_24_P213321 |  | 1.11 | 1.75E-02 |
| A_23_P85893 | Homo sapiens chromosome 1 open reading frame 85 (C1orf85), mRNA [NM_144580] | 1.11 | 2.45E-03 |
| A_23_P130865 | Homo sapiens hypothetical protein FLJ10374 (FLJ10374), mRNA [NM_018074] | 1.11 | 3.14E-02 |
| A_23_P121396 | Homo sapiens DnaJ (Hsp40) homolog, subfamily C, member 19 (DNAJC19), transcript variant 1, mRNA [NM_145261] | 1.11 | 2.91E-02 |
| A_23_P139919 | Homo sapiens carbohydrate (chondroitin 4) sulfotransferase 11 (CHST11), mRNA [NM_018413] | 1.11 | 3.36E-03 |
| A_24_P124558 | Homo sapiens homeo box C8 (HOXC8), mRNA [NM_022658] | 1.11 | 2.18E-02 |
| A_23_P205046 | Homo sapiens ankyrin repeat domain 10 (ANKRD10), mRNA [NM_017664] | 1.11 | 3.95E-02 |
| A_23_P202484 | Homo sapiens zinc finger protein 503 (ZNF503), mRNA [NM_032772] | 1.11 | 5.67E-03 |
| A_24_P339611 | Homo sapiens programmed cell death 5 (PDCD5), mRNA [NM_004708] | 1.11 | 4.04E-02 |
| A_23_P91076 | full-length cDNA clone CS0DL008YP09 of B cells (Ramos cell line) Cot 25-normalized of Homo sapiens (human). [CR621710] | 1.11 | 3.61E-02 |
| A_24_P168416 | Homo sapiens peroxiredoxin 2 (PRDX2), nuclear gene encoding mitochondrial protein, transcript variant 3, mRNA [NM_181738] | 1.11 | 4.26E-02 |
| A_32_P155035 | Homo sapiens cDNA FLJ39181 fis, clone OCBBF2004235. [AK096500] | 1.11 | 1.33E-02 |
| A_23_P211196 | Homo sapiens chromosome 21 open reading frame 67 (C21orf67), mRNA [NM_058188] | 1.11 | 2.70E-02 |
| A_23_P329286 | Homo sapiens zinc finger, HIT domain containing 2 (ZNHIT2), mRNA [NM_014205] | 1.11 | 4.81E-03 |
| A_24_P29001 | Homo sapiens LSM3 homolog, U6 small nuclear RNA associated (S. cerevisiae) (LSM3), mRNA [NM_014463] | 1.11 | 4.17E-03 |
| A_23_P17998 | Homo sapiens hairy and enhancer of split 1, (Drosophila) (HES1), mRNA [NM_005524] | 1.11 | 1.17E-02 |
| A_23_P20384 | Homo sapiens LSM1 homolog, U6 small nuclear RNA associated (S. cerevisiae) (LSM1), mRNA [NM_014462] | 1.11 | 4.68E-02 |
| A_23_P252201 | Homo sapiens ELL associated factor 2 (EAF2), mRNA [NM_018456] | 1.10 | 3.78E-02 |
| A_24_P813520 | full-length cDNA clone CS0DI005YB15 of Placenta Cot 25-normalized of Homo sapiens (human). [CR626222] | 1.10 | 7.26E-03 |
| A_24_P694738 | Homo sapiens mRNA; cDNA DKFZp686B0328 (from clone DKFZp686B0328). [BX640887] | 1.10 | 2.00E-02 |
| A_23_P202720 | Homo sapiens solute carrier family 35, member C1 (SLC35C1), mRNA [NM_018389] | 1.10 | 2.32E-02 |
| A_23_P109636 | Homo sapiens leucine-rich repeats and immunoglobulin-like domains 1 (LRIG1), mRNA [NM_015541] | 1.10 | 2.05E-02 |
| A_32_P75425 | Homo sapiens hypothetical LOC399744 (LOC399744), mRNA [NM_001013665] | 1.10 | 3.83E-02 |
| A_23_P167096 | Homo sapiens vascular endothelial growth factor C (VEGFC), mRNA [NM_005429] | 1.10 | 3.87E-02 |
| A_24_P592544 | Q5XI42 (Q5XI42) Fatty aldehyde dehydrogenase-like, partial (5%) [THC2399998] | 1.10 | 3.14E-02 |
| A_23_P26916 | Homo sapiens histone deacetylase 5 (HDAC5), transcript variant 3, mRNA [NM_001015053] | 1.10 | 1.04E-02 |
| A_23_P89589 | Homo sapiens period homolog 1 (Drosophila) (PER1), mRNA [NM_002616] | 1.10 | 2.66E-02 |
| A_23_P79122 | Homo sapiens uncharacterized hematopoietic stem/progenitor cells protein MDS032 (MDS032), mRNA [NM_018467] | 1.10 | 2.49E-02 |
| A_23_P252145 | Homo sapiens core 1 synthase, glycoprotein-N-acetylgalactosamine 3-beta-galactosyltransferase, 1 (C1GALT1), mRNA [NM_020156] | 1.10 | 4.43E-02 |
| A_23_P112512 | Homo sapiens mitochondrial carrier triple repeat 1 (MCART1), mRNA [NM_033412] | 1.10 | 4.17E-02 |
| A_24_P233663 | Homo sapiens PCTAIRE protein kinase 1 (PCTK1), transcript variant 2, mRNA [NM_033018] | 1.10 | 3.89E-02 |
| A_32_P61061 | Homo sapiens peptidylprolyl isomerase A-like (LOC388817), mRNA [NM_001008741] | 1.10 | 4.41E-02 |
| A_32_P222695 | Homo sapiens FLJ41603 protein (FLJ41603), mRNA [NM_001001669] | 1.10 | 4.36E-02 |
| A_23_P63281 | Homo sapiens hypothetical protein MGC10334 (MGC10334), mRNA [NM_001029885] | -1.10 | 2.12E-02 |
| A_23_P14157 | Homo sapiens DAZ interacting protein 1 (DZIP1), mRNA [NM_198968] | -1.10 | 2.58E-02 |
| A_23_P1676 | full-length cDNA clone CS0DK012YH13 of HeLa cells Cot 25-normalized of Homo sapiens (human). [CR593246] | -1.10 | 1.78E-02 |
| A_23_P401380 | Homo sapiens KIAA1463 protein (KIAA1463), mRNA [NM_173602] | -1.10 | 1.71E-02 |
| A_23_P2097 | Homo sapiens tripartite motif-containing 68 (TRIM68), mRNA [NM_018073] | -1.10 | 4.76E-02 |
| A_24_P56484 | Homo sapiens breast cancer metastasis-suppressor 1-like (BRMS1L), mRNA [NM_032352] | -1.10 | 2.32E-02 |
| A_32_P51119 | Homo sapiens storkhead box 1 (STOX1), mRNA [NM_152709] | -1.10 | 4.67E-02 |
| A_23_P94911 | Homo sapiens cDNA FLJ40856 fis, clone TRACH2016498, moderately similar to ZINC FINGER PROTEIN 184. [AK098175] | -1.10 | 4.96E-02 |
| A_23_P115375 | Homo sapiens histone H3/o (H3/o), mRNA [NM_001005464] | -1.10 | 2.03E-03 |
| A_23_P94063 | Homo sapiens truncated zinc finger protein 36 mRNA, complete cds. [AY260738] | -1.10 | 4.43E-02 |
| A_23_P396626 | Homo sapiens AP1 gamma subunit binding protein 1 (AP1GBP1), transcript variant 1, mRNA [NM_007247] | -1.10 | 1.00E-02 |
| A_23_P136817 | Homo sapiens kinetochore associated 1 (KNTC1), mRNA [NM_014708] | -1.10 | 3.26E-02 |
| A_23_P253375 | Homo sapiens cut-like 1, CCAAT displacement protein (Drosophila) (CUTL1), transcript variant 2, mRNA [NM_001913] | -1.10 | 4.12E-02 |
| A_23_P250404 | Homo sapiens RAD50 homolog (S. cerevisiae) (RAD50), transcript variant 1, mRNA [NM_005732] | -1.10 | 4.21E-02 |
| A_32_P99690 | UI-E-CQ1-afy-b-13-0-UI.r1 UI-E-CQ1 Homo sapiens cDNA clone UI-E-CQ1-afy-b-13-0-UI 5', mRNA sequence [BM709498] | -1.10 | 4.32E-02 |
| A_23_P35684 | Homo sapiens inositol polyphosphate-5-phosphatase F (INPP5F), transcript variant 1, mRNA [NM_014937] | -1.10 | 6.71E-04 |
| A_23_P118185 | Homo sapiens peroxisomal lon protease (LONP), mRNA [NM_031490] | -1.10 | 4.47E-02 |
| A_23_P166609 | Homo sapiens DEAH (Asp-Glu-Ala-His) box polypeptide 30 (DHX30), transcript variant 2, mRNA [NM_014966] | -1.10 | 2.54E-02 |
| A_23_P170337 | Homo sapiens aldehyde dehydrogenase 4 family, member A1 (ALDH4A1), nuclear gene encoding mitochondrial protein, transcript variant P5CDhL, mRNA [NM_003748] | -1.10 | 1.43E-02 |
| A_24_P396105 | Homo sapiens inositol hexaphosphate kinase 1 (IHPK1), transcript variant 1, mRNA [NM_153273] | -1.10 | 7.97E-03 |
| A_23_P163047 | Homo sapiens chromosome 14 open reading frame 150 (C14orf150), transcript variant 1, mRNA [NM_001008726] | -1.10 | 1.87E-02 |
| A_23_P169112 | Homo sapiens cleavage and polyadenylation specific factor 1, 160kDa (CPSF1), mRNA [NM_013291] | -1.10 | 1.09E-02 |
| A_23_P211797 | Homo sapiens optic atrophy 1 (autosomal dominant) (OPA1), nuclear gene encoding mitochondrial protein, transcript variant 8, mRNA [NM_130837] | -1.10 | 2.90E-02 |
| A_24_P157165 | Homo sapiens mitogen-activated protein kinase kinase kinase kinase 4 (MAP4K4), transcript variant 2, mRNA [NM_145686] | -1.10 | 4.60E-02 |
| A_23_P164237 | Homo sapiens chromosome 17 open reading frame 40 (C17orf40), mRNA [NM_018428] | -1.10 | 6.21E-04 |
| A_23_P216068 | Homo sapiens ATPase family, AAA domain containing 2 (ATAD2), mRNA [NM_014109] | -1.10 | 3.13E-02 |
| A_23_P128650 | Homo sapiens solute carrier family 25 (mitochondrial carrier; ornithine transporter) member 15 (SLC25A15), nuclear gene encoding mitochondrial protein, mRNA [NM_014252] | -1.10 | 9.12E-03 |
| A_23_P5742 | Homo sapiens hypothetical protein FLJ13646 (FLJ13646), mRNA [NM_024584] | -1.11 | 1.52E-02 |
| A_24_P289845 | full-length cDNA clone CS0DD008YI13 of Neuroblastoma Cot 50-normalized of Homo sapiens (human). [CR625571] | -1.11 | 2.95E-02 |
| A_23_P9086 | Homo sapiens zinc finger, DHHC-type containing 2 (ZDHHC2), mRNA [NM_016353] | -1.11 | 4.27E-03 |
| A_24_P411749 | Homo sapiens G protein-coupled receptor 126 (GPR126), mRNA [NM_198569] | -1.11 | 2.44E-02 |
| A_23_P306500 | Homo sapiens v-Ki-ras2 Kirsten rat sarcoma viral oncogene homolog (KRAS), transcript variant a, mRNA [NM_033360] | -1.11 | 3.94E-02 |
| A_23_P79587 | Homo sapiens alkaline phosphatase, placental (Regan isozyme) (ALPP), mRNA [NM_001632] | -1.11 | 3.27E-02 |
| A_23_P155301 | Homo sapiens NIMA (never in mitosis gene a)- related kinase 11 (NEK11), transcript variant 2, mRNA [NM_145910] | -1.11 | 7.45E-03 |
| A_23_P374389 | Homo sapiens PWWP domain containing 2 (PWWP2), mRNA [NM_138499] | -1.11 | 4.11E-02 |
| A_23_P400235 | Homo sapiens methylmalonyl Coenzyme A mutase (MUT), nuclear gene encoding mitochondrial protein, mRNA [NM_000255] | -1.11 | 3.42E-02 |
| A_23_P75038 | Homo sapiens DNA cross-link repair 1A (PSO2 homolog, S. cerevisiae) (DCLRE1A), mRNA [NM_014881] | -1.11 | 8.35E-03 |
| A_23_P364478 | Homo sapiens KIAA0157 (KIAA0157), mRNA [NM_032182] | -1.11 | 1.40E-02 |
| A_23_P26905 | Homo sapiens polymerase (DNA directed), gamma 2, accessory subunit (POLG2), mRNA [NM_007215] | -1.11 | 3.71E-02 |
| A_23_P203344 | Homo sapiens zinc finger protein 91 homolog (mouse) (ZFP91), transcript variant 1, mRNA [NM_053023] | -1.11 | 6.59E-03 |
| A_24_P112750 | Homo sapiens transcription factor CP2 (TFCP2), mRNA [NM_005653] | -1.11 | 2.97E-02 |
| A_23_P38860 | Q96GV2 (Q96GV2) XTP7, complete [THC2262919] | -1.11 | 1.05E-02 |
| A_23_P37347 | Homo sapiens SKI interacting protein (SKIIP), mRNA [NM_012245] | -1.11 | 2.50E-02 |
| A_24_P830667 | Homo sapiens ribosomal protein L21 (RPL21), mRNA [NM_000982] | -1.11 | 2.11E-02 |
| A_23_P34325 | Homo sapiens low density lipoprotein receptor-related protein 8, apolipoprotein e receptor (LRP8), transcript variant 2, mRNA [NM_033300] | -1.11 | 3.64E-02 |
| A_23_P501770 | Homo sapiens three prime repair exonuclease 1 (TREX1), transcript variant 5, mRNA [NM_032166] | -1.11 | 2.16E-02 |
| A_23_P145053 | Homo sapiens tubulin, epsilon 1 (TUBE1), mRNA [NM_016262] | -1.11 | 7.58E-03 |
| A_23_P143748 | Homo sapiens KIAA0153 protein (KIAA0153), mRNA [NM_015140] | -1.11 | 4.99E-02 |
| A_23_P86632 | Homo sapiens DNA cross-link repair 1C (PSO2 homolog, S. cerevisiae) (DCLRE1C), mRNA [NM_022487] | -1.11 | 1.95E-02 |
| A_23_P319423 | Homo sapiens potassium channel, subfamily K, member 5 (KCNK5), mRNA [NM_003740] | -1.11 | 1.28E-02 |
| A_23_P251196 |  | -1.11 | 1.92E-02 |
| A_24_P305623 | Homo sapiens transmembrane protein 50B (TMEM50B), mRNA [NM_006134] | -1.11 | 2.23E-02 |
| A_24_P291598 | Homo sapiens ubiquitin specific protease 4 (proto-oncogene) (USP4), transcript variant 1, mRNA [NM_003363] | -1.11 | 4.01E-02 |
| A_23_P99405 | Homo sapiens zinc finger protein 198 (ZNF198), mRNA [NM_003453] | -1.11 | 1.90E-02 |
| A_24_P11965 | Homo sapiens Mof4 family associated protein 1 (MRFAP1), mRNA [NM_033296] | -1.11 | 2.93E-02 |
| A_24_P335358 | Homo sapiens pseudouridylate synthase 1 (PUS1), transcript variant 1, mRNA [NM_025215] | -1.11 | 1.95E-03 |
| A_32_P1445 | Homo sapiens protein tyrosine phosphatase, non-receptor type 2 (PTPN2), transcript variant 3, mRNA [NM_080423] | -1.11 | 3.57E-02 |
| A_23_P138137 | Homo sapiens OMA1 homolog, zinc metallopeptidase (S. cerevisiae) (OMA1), mRNA [NM_145243] | -1.11 | 2.20E-02 |
| A_24_P37519 | Homo sapiens leucine zipper transcription factor-like 1 (LZTFL1), mRNA [NM_020347] | -1.11 | 1.23E-03 |
| A_23_P258972 | Homo sapiens golgi autoantigen, golgin subfamily a, 1 (GOLGA1), mRNA [NM_002077] | -1.11 | 3.01E-02 |
| A_24_P913339 | Homo sapiens chromosome 2 open reading frame 18, mRNA (cDNA clone IMAGE:3860139), complete cds. [BC016389] | -1.11 | 8.19E-03 |
| A_24_P570583 | Homo sapiens zinc finger protein 542 (ZNF542), mRNA [NM_194319] | -1.11 | 2.03E-02 |
| A_23_P502158 | Homo sapiens a disintegrin and metalloproteinase domain 11 (ADAM11), transcript variant 1, mRNA [NM_002390] | -1.11 | 4.85E-02 |
| A_23_P258251 | Homo sapiens cytosolic ovarian carcinoma antigen 1 (COVA1), transcript variant 2, mRNA [NM_182314] | -1.11 | 6.98E-03 |
| A_24_P693986 | Homo sapiens hypothetical LOC388610 (LOC388610), mRNA [NM_001013642] | -1.11 | 1.80E-02 |
| A_23_P379327 | Homo sapiens mRNA for KIAA1164 protein, partial cds. [AB032990] | -1.11 | 4.04E-02 |
| A_23_P109436 | Homo sapiens adenosine A2a receptor (ADORA2A), mRNA [NM_000675] | -1.11 | 2.04E-04 |
| A_23_P59787 | Homo sapiens LUC7-like 2 (S. cerevisiae) (LUC7L2), mRNA [NM_016019] | -1.11 | 3.32E-02 |
| A_23_P71591 | Homo sapiens nucleolar protein 8 (NOL8), mRNA [NM_017948] | -1.11 | 3.33E-03 |
| A_23_P151471 | Homo sapiens cullin 4A (CUL4A), transcript variant 1, mRNA [NM_001008895] | -1.11 | 1.35E-02 |
| A_23_P323751 | Homo sapiens chromosome 20 open reading frame 129 (C20orf129), mRNA [NM_030919] | -1.11 | 1.75E-02 |
| A_23_P169934 | Homo sapiens hypothetical protein FLJ39378 (FLJ39378), mRNA [NM_178314] | -1.11 | 1.33E-02 |
| A_23_P118327 | Homo sapiens THUMP domain containing 1 (THUMPD1), mRNA [NM_017736] | -1.11 | 1.81E-02 |
| A_24_P156113 | Homo sapiens EH-domain containing 2 (EHD2), mRNA [NM_014601] | -1.11 | 3.39E-02 |
| A_23_P211207 | Homo sapiens adenosine deaminase, RNA-specific, B1 (RED1 homolog rat) (ADARB1), transcript variant DRABA2b, mRNA [NM_015833] | -1.11 | 4.43E-02 |
| A_24_P912856 |  | -1.11 | 6.49E-03 |
| A_23_P257057 | Homo sapiens mesenchymal stem cell protein DSCD75 (LOC51337), mRNA [NM_016647] | -1.11 | 4.31E-03 |
| A_23_P94795 | Homo sapiens TEA domain family member 4 (TEAD4), transcript variant 1, mRNA [NM_003213] | -1.11 | 2.97E-02 |
| A_24_P58597 |  | -1.11 | 1.22E-02 |
| A_23_P156310 | Homo sapiens S-phase kinase-associated protein 2 (p45) (SKP2), transcript variant 2, mRNA [NM_032637] | -1.11 | 3.53E-03 |
| A_23_P75453 | Homo sapiens multiple endocrine neoplasia I (MEN1), transcript variant e1E, mRNA [NM_130803] | -1.11 | 3.73E-02 |
| A_24_P47988 | Homo sapiens elongation factor RNA polymerase II-like 3 (ELL3), mRNA [NM_025165] | -1.11 | 3.78E-02 |
| A_24_P304760 | M2C1_HUMAN (Q9NTJ4) Alpha-mannosidase 2C1 (Alpha-D-mannoside mannohydrolase) (Mannosidase alpha class 2C member 1) (Alpha mannosidase 6A8B), partial (7%) [THC2372800] | -1.11 | 1.15E-02 |
| A_23_P120316 | Homo sapiens methylenetetrahydrofolate dehydrogenase (NADP+ dependent) 2, methenyltetrahydrofolate cyclohydrolase (MTHFD2), nuclear gene encoding mitochondrial protein, mRNA [NM_006636] | -1.11 | 3.12E-02 |
| A_24_P220058 | Homo sapiens microtubule-associated protein, RP/EB family, member 1 (MAPRE1), mRNA [NM_012325] | -1.11 | 3.56E-02 |
| A_23_P37785 | Homo sapiens potassium channel tetramerisation domain containing 19, mRNA (cDNA clone IMAGE:5268205). [BC070103] | -1.11 | 4.29E-02 |
| A_23_P310911 | Homo sapiens bleomycin hydrolase (BLMH), mRNA [NM_000386] | -1.11 | 1.77E-02 |
| A_23_P54230 | Homo sapiens nuclear protein UKp68 (FLJ11806), transcript variant 2, mRNA [NM_207660] | -1.12 | 1.30E-02 |
| A_23_P30275 | Homo sapiens hypothetical protein MGC3265 (MGC3265), mRNA [NM_024028] | -1.12 | 4.57E-02 |
| A_23_P353056 | Homo sapiens transmembrane protein 24 (TMEM24), mRNA [NM_014807] | -1.12 | 4.01E-02 |
| A_23_P21785 | Homo sapiens NOL1/NOP2/Sun domain family, member 3 (NSUN3), mRNA [NM_022072] | -1.12 | 4.62E-02 |
| A_23_P323743 | Homo sapiens chromosome 15 open reading frame 20 (C15orf20), mRNA [NM_025049] | -1.12 | 2.43E-02 |
| A_24_P84970 | PREDICTED: Homo sapiens similar to Keratin, type I cytoskeletal 18 (Cytokeratin 18) (K18) (CK 18) (LOC391819), mRNA [XM_498013] | -1.12 | 4.20E-03 |
| A_23_P46748 | Homo sapiens conserved helix-loop-helix ubiquitous kinase (CHUK), mRNA [NM_001278] | -1.12 | 4.39E-03 |
| A_23_P129629 | Homo sapiens metallothionein 3 (growth inhibitory factor (neurotrophic)) (MT3), mRNA [NM_005954] | -1.12 | 4.60E-02 |
| A_23_P155857 | Homo sapiens nudix (nucleoside diphosphate linked moiety X)-type motif 6 (NUDT6), transcript variant 2, mRNA [NM_198041] | -1.12 | 4.24E-02 |
| A_32_P459533 | Homo sapiens FCH domain only 1 (FCHO1), mRNA [NM_015122] | -1.12 | 1.93E-02 |
| A_23_P42997 | Homo sapiens cleavage and polyadenylation specific factor 4, 30kDa (CPSF4), mRNA [NM_006693] | -1.12 | 4.61E-02 |
| A_23_P80902 | Homo sapiens kinesin family member 15 (KIF15), mRNA [NM_020242] | -1.12 | 1.40E-02 |
| A_23_P104282 | Homo sapiens chromosome 10 open reading frame 6 (C10orf6), mRNA [NM_018121] | -1.12 | 2.79E-02 |
| A_24_P167614 | Homo sapiens DEAD/H (Asp-Glu-Ala-Asp/His) box polypeptide 26 (DDX26), mRNA [NM_012141] | -1.12 | 1.00E-02 |
| A_24_P409881 | PREDICTED: Homo sapiens similar to hypothetical protein (LOC338756), mRNA [XM_291989] | -1.12 | 2.87E-02 |
| A_23_P5550 | Homo sapiens pumilio homolog 2 (Drosophila) (PUM2), mRNA [NM_015317] | -1.12 | 1.62E-02 |
| A_23_P67583 | Homo sapiens BPY2 interacting protein 1 (BPY2IP1), mRNA [NM_018174] | -1.12 | 4.01E-03 |
| A_24_P388536 | Homo sapiens hypothetical protein PRO2730 (PRO2730), mRNA [NM_025222] | -1.12 | 4.75E-02 |
| A_23_P386 | Homo sapiens Rho guanine nucleotide exchange factor (GEF) 10-like (ARHGEF10L), transcript variant 1, mRNA [NM_018125] | -1.12 | 3.87E-02 |
| A_23_P66355 | Homo sapiens integrin, beta 4 (ITGB4), transcript variant 1, mRNA [NM_000213] | -1.12 | 4.41E-03 |
| A_24_P248606 | Homo sapiens acyl-CoA synthetase long-chain family member 3 (ACSL3), transcript variant 1, mRNA [NM_004457] | -1.12 | 2.67E-02 |
| A_23_P162807 | Homo sapiens mitochondrial ribosomal protein S31 (MRPS31), nuclear gene encoding mitochondrial protein, mRNA [NM_005830] | -1.12 | 2.67E-02 |
| A_23_P66158 | full-length cDNA clone CS0DI060YI16 of Placenta Cot 25-normalized of Homo sapiens (human). [CR625565] | -1.12 | 3.12E-02 |
| A_24_P379104 | Homo sapiens pim-2 oncogene (PIM2), mRNA [NM_006875] | -1.12 | 3.31E-02 |
| A_23_P345820 | Homo sapiens WD repeat and FYVE domain containing 3 (WDFY3), transcript variant 1, mRNA [NM_014991] | -1.12 | 1.63E-02 |
| A_23_P414884 | Homo sapiens corticotropin releasing hormone receptor 1 (CRHR1), mRNA [NM_004382] | -1.12 | 9.83E-03 |
| A_24_P170874 | Homo sapiens cDNA clone IMAGE:2960340. [BC013295] | -1.12 | 1.30E-03 |
| A_23_P122624 | Homo sapiens chromosome 6 open reading frame 93 (C6orf93), mRNA [NM_032860] | -1.12 | 1.14E-02 |
| A_23_P325075 | Homo sapiens RNA guanylyltransferase and 5'-phosphatase (RNGTT), mRNA [NM_003800] | -1.12 | 2.32E-02 |
| A_23_P395493 | Homo sapiens cofilin pseudogene 1, mRNA (cDNA clone IMAGE:5168640). [BC031631] | -1.12 | 4.58E-02 |
| A_23_P207967 | Homo sapiens KIAA0427 (KIAA0427), mRNA [NM_014772] | -1.12 | 1.83E-02 |
| A_24_P38081 | Homo sapiens FK506 binding protein 5 (FKBP5), mRNA [NM_004117] | -1.12 | 8.06E-03 |
| A_32_P226700 | Q6STG2 (Q6STG2) DNA polymerase-transactivated protein 3, partial (13%) [THC2438975] | -1.12 | 1.08E-02 |
| A_23_P107513 | Homo sapiens chromosome 18 open reading frame 9 (C18orf9), mRNA [NM_024899] | -1.12 | 1.55E-02 |
| A_23_P99320 | Homo sapiens keratin 18 (KRT18), transcript variant 1, mRNA [NM_000224] | -1.12 | 3.35E-02 |
| A_23_P345707 | Homo sapiens leucine-rich repeat kinase 1 (MGC45866), mRNA [NM_152259] | -1.12 | 8.74E-03 |
| A_23_P70384 | Homo sapiens ring finger protein 8 (RNF8), transcript variant 1, mRNA [NM_003958] | -1.12 | 2.80E-02 |
| A_23_P169358 | Homo sapiens syntaxin 17 (STX17), mRNA [NM_017919] | -1.12 | 1.23E-02 |
| A_24_P205268 | Homo sapiens KIAA0323 (KIAA0323), mRNA [NM_015299] | -1.12 | 2.53E-02 |
| A_23_P148121 | Homo sapiens mRNA; cDNA DKFZp762C186 (from clone DKFZp762C186). [AL834433] | -1.12 | 3.42E-02 |
| A_23_P36860 | Homo sapiens La ribonucleoprotein domain family, member 4 (LARP4), transcript variant 2, mRNA [NM_199188] | -1.12 | 3.53E-02 |
| A_23_P217968 | Homo sapiens suppressor of variegation 4-20 homolog 1 (Drosophila) (SUV420H1), transcript variant 2, mRNA [NM_016028] | -1.12 | 3.06E-02 |
| A_23_P54605 | Homo sapiens ribosomal L1 domain containing 1 (RSL1D1), mRNA [NM_015659] | -1.12 | 8.68E-03 |
| A_23_P327907 | Homo sapiens chromosome 8 open reading frame 37 (C8orf37), mRNA [NM_177965] | -1.12 | 4.29E-02 |
| A_23_P82738 | Homo sapiens RAD54 homolog B (S. cerevisiae) (RAD54B), transcript variant 1, mRNA [NM_012415] | -1.12 | 4.15E-02 |
| A_23_P31550 | Homo sapiens cDNA FLJ11871 fis, clone HEMBA1007052. [AK021933] | -1.12 | 1.56E-02 |
| A_23_P92602 | Homo sapiens cDNA FLJ14297 fis, clone PLACE1008941. [AK024359] | -1.12 | 4.33E-02 |
| A_24_P337104 | Homo sapiens oxytocin receptor (OXTR), mRNA [NM_000916] | -1.12 | 2.27E-02 |
| A_23_P152919 | Homo sapiens nucleoporin 88kDa (NUP88), mRNA [NM_002532] | -1.12 | 2.35E-02 |
| A_23_P4425 | Homo sapiens flightless I homolog (Drosophila) (FLII), mRNA [NM_002018] | -1.12 | 1.62E-02 |
| A_24_P305541 | Homo sapiens tribbles homolog 3 (Drosophila) (TRIB3), mRNA [NM_021158] | -1.12 | 4.51E-02 |
| A_24_P344307 | Homo sapiens proteasome (prosome, macropain) activator subunit 3 (PA28 gamma; Ki) (PSME3), transcript variant 2, mRNA [NM_176863] | -1.12 | 9.27E-03 |
| A_23_P102832 | Homo sapiens centrosomal protein 2 (CEP2), mRNA [NM_007186] | -1.12 | 5.00E-02 |
| A_32_P74477 |  | -1.13 | 2.66E-02 |
| A_23_P366468 |  | -1.13 | 1.11E-02 |
| A_24_P202567 | Homo sapiens inositol 1,4,5-trisphosphate 3-kinase C (ITPKC), mRNA [NM_025194] | -1.13 | 1.09E-02 |
| A_24_P237766 | Homo sapiens SEC14-like 1 (S. cerevisiae) (SEC14L1), mRNA [NM_003003] | -1.13 | 3.79E-02 |
| A_32_P24965 | Homo sapiens zinc finger, FYVE domain containing 26 (ZFYVE26), mRNA [NM_015346] | -1.13 | 2.47E-02 |
| A_23_P408768 | Homo sapiens DOT1-like, histone H3 methyltransferase (S. cerevisiae) (DOT1L), mRNA [NM_032482] | -1.13 | 3.13E-02 |
| A_24_P381945 | Homo sapiens heme oxygenase (decycling) 2 (HMOX2), mRNA [NM_002134] | -1.13 | 3.16E-02 |
| A_24_P229658 | PREDICTED: Homo sapiens similar to hypothetical protein (LOC391804), mRNA [XM_498008] | -1.13 | 3.14E-02 |
| A_24_P98613 | Homo sapiens tetraspanin 14 (TSPAN14), mRNA [NM_030927] | -1.13 | 2.30E-02 |
| A_24_P50543 | Homo sapiens, clone IMAGE:5277162, mRNA. [BC031266] | -1.13 | 3.12E-02 |
| A_32_P68408 | Homo sapiens, clone IMAGE:5166482, mRNA, partial cds. [BC028192] | -1.13 | 3.92E-02 |
| A_24_P115007 | Homo sapiens aldehyde dehydrogenase 5 family, member A1 (succinate-semialdehyde dehydrogenase) (ALDH5A1), nuclear gene encoding mitochondrial protein, transcript variant 1, mRNA [NM_170740] | -1.13 | 4.03E-02 |
| A_23_P157600 | Homo sapiens DDHD domain containing 2 (DDHD2), mRNA [NM_015214] | -1.13 | 1.92E-02 |
| A_23_P253524 | Homo sapiens centromere protein E, 312kDa (CENPE), mRNA [NM_001813] | -1.13 | 9.71E-03 |
| A_23_P38115 | Homo sapiens hypothetical protein FLJ20291 (FLJ20291), mRNA [NM_017748] | -1.13 | 2.47E-02 |
| A_23_P161091 | Homo sapiens zinc finger, MYM domain containing 1 (ZMYM1), mRNA [NM_024772] | -1.13 | 2.87E-02 |
| A_24_P278460 | Homo sapiens male sterility domain containing 2 (MLSTD2), mRNA [NM_032228] | -1.13 | 1.89E-03 |
| A_24_P7157 | Homo sapiens family with sequence similarity 80, member B (FAM80B), mRNA [NM_020734] | -1.13 | 8.10E-03 |
| A_23_P431981 | Homo sapiens high-mobility group protein 2-like 1 (HMG2L1), transcript variant 1, mRNA [NM_005487] | -1.13 | 9.46E-03 |
| A_24_P413470 | Homo sapiens tumor protein p73 (TP73), mRNA [NM_005427] | -1.13 | 3.44E-02 |
| A_23_P386942 | Homo sapiens DIRAS family, GTP-binding RAS-like 1 (DIRAS1), mRNA [NM_145173] | -1.13 | 1.65E-02 |
| A_23_P380815 | Homo sapiens KIAA1279 (KIAA1279), mRNA [NM_015634] | -1.13 | 2.76E-02 |
| A_23_P17444 | BG680979 602628792F1 NCI_CGAP_Skn4 Homo sapiens cDNA clone IMAGE:4753583 5', mRNA sequence [BG680979] | -1.13 | 2.72E-02 |
| A_32_P217510 | Homo sapiens WD repeat domain 75 (WDR75), mRNA [NM_032168] | -1.13 | 3.68E-02 |
| A_23_P502078 | Homo sapiens mitogen-activated protein kinase 8 interacting protein 2 (MAPK8IP2), transcript variant 1, mRNA [NM_012324] | -1.13 | 4.79E-02 |
| A_24_P264644 | PREDICTED: Homo sapiens similar to Keratin, type I cytoskeletal 18 (Cytokeratin 18) (K18) (CK 18) (LOC345430), mRNA [XM_498024] | -1.13 | 4.88E-02 |
| A_24_P195400 |  | -1.13 | 2.99E-02 |
| A_24_P472455 | Homo sapiens mRNA; cDNA DKFZp564M0264 (from clone DKFZp564M0264). [AL117621] | -1.13 | 4.14E-02 |
| A_23_P398073 | Homo sapiens protein phosphatase 1B (formerly 2C), magnesium-dependent, beta isoform (PPM1B), transcript variant 2, mRNA [NM_177968] | -1.13 | 2.05E-02 |
| A_23_P334630 | Homo sapiens jerky homolog (mouse) (JRK), mRNA [NM_003724] | -1.13 | 6.33E-03 |
| A_24_P942002 | Homo sapiens centaurin, beta 2 (CENTB2), mRNA [NM_012287] | -1.13 | 3.72E-02 |
| A_24_P225468 | Homo sapiens acidic (leucine-rich) nuclear phosphoprotein 32 family, member E (ANP32E), mRNA [NM_030920] | -1.13 | 1.88E-02 |
| A_23_P104555 | Homo sapiens ankyrin repeat domain 2 (stretch responsive muscle) (ANKRD2), mRNA [NM_020349] | -1.13 | 2.59E-02 |
| A_23_P153676 | Homo sapiens transducin-like enhancer of split 2 (E(sp1) homolog, Drosophila) (TLE2), mRNA [NM_003260] | -1.13 | 6.77E-03 |
| A_23_P368205 | Homo sapiens phosphatidylinositol-4-phosphate 5-kinase, type I, alpha (PIP5K1A), mRNA [NM_003557] | -1.13 | 2.70E-02 |
| A_24_P415260 | Homo sapiens cDNA FLJ36123 fis, clone TESTI2022874, weakly similar to ZINC FINGER PROTEIN 135. [AK093442] | -1.13 | 3.92E-02 |
| A_24_P281443 |  | -1.13 | 6.33E-03 |
| A_24_P377328 | Homo sapiens step II splicing factor SLU7 (SLU7), mRNA [NM_006425] | -1.13 | 4.79E-02 |
| A_24_P35478 | Homo sapiens par-3 partitioning defective 3 homolog (C. elegans) (PARD3), mRNA [NM_019619] | -1.13 | 4.86E-02 |
| A_24_P933418 | Homo sapiens cDNA FLJ30301 fis, clone BRACE2003217. [AK054863] | -1.13 | 7.69E-03 |
| A_23_P358470 | Homo sapiens hypothetical protein FLJ33167 (FLJ33167), mRNA [NM_152683] | -1.13 | 1.34E-02 |
| A_23_P319583 | Homo sapiens regulating synaptic membrane exocytosis 3 (RIMS3), mRNA [NM_014747] | -1.13 | 4.02E-02 |
| A_23_P117734 | Homo sapiens hypothetical protein FLJ33008 (FLJ33008), mRNA [NM_152449] | -1.13 | 3.26E-02 |
| A_24_P204358 | Homo sapiens pyrroline-5-carboxylate reductase 1 (PYCR1), transcript variant 2, mRNA [NM_153824] | -1.13 | 4.42E-02 |
| A_23_P101796 | Homo sapiens synapse defective 1, Rho GTPase, homolog 1 (C. elegans) (SYDE1), mRNA [NM_033025] | -1.13 | 2.55E-02 |
| A_23_P34176 | Homo sapiens KIAA1280 protein (KIAA1280), mRNA [NM_015691] | -1.13 | 1.79E-02 |
| A_23_P154500 | Homo sapiens DNA (cytosine-5-)-methyltransferase 3 alpha (DNMT3A), transcript variant 1, mRNA [NM_175629] | -1.13 | 2.97E-02 |
| A_24_P210577 | Homo sapiens modulator of estrogen induced transcription (FLJ13213), transcript variant 1, mRNA [NM_024755] | -1.13 | 2.17E-02 |
| A_24_P922877 | Homo sapiens kinesin light chain mRNA, complete cds. [L04733] | -1.13 | 1.10E-02 |
| A_24_P218979 | Homo sapiens cell division cycle associated 3 (CDCA3), mRNA [NM_031299] | -1.13 | 3.53E-02 |
| A_23_P138465 | Homo sapiens nucleolar and coiled-body phosphoprotein 1 (NOLC1), mRNA [NM_004741] | -1.13 | 3.73E-02 |
| A_23_P102925 | Homo sapiens PWP2 periodic tryptophan protein homolog (yeast) (PWP2H), mRNA [NM_005049] | -1.13 | 3.22E-02 |
| A_24_P114339 | full-length cDNA clone CS0DF020YB09 of Fetal brain of Homo sapiens (human). [CR604908] | -1.14 | 2.49E-02 |
| A_23_P358957 | Homo sapiens calcium/calmodulin-dependent protein kinase kinase 2, beta (CAMKK2), transcript variant 1, mRNA [NM_006549] | -1.14 | 2.73E-02 |
| A_32_P319880 | Homo sapiens KIAA1530 protein (KIAA1530), mRNA [NM_020894] | -1.14 | 3.33E-02 |
| A_23_P54720 | Homo sapiens hypothetical protein LOC201725 (LOC201725), mRNA [NM_001008393] | -1.14 | 2.63E-02 |
| A_24_P242609 | Homo sapiens kelch-like 12 (Drosophila) (KLHL12), mRNA [NM_021633] | -1.14 | 3.95E-02 |
| A_23_P9768 | Homo sapiens LYST-interacting protein LIP8 (LIP8), mRNA [NM_053051] | -1.14 | 3.94E-03 |
| A_32_P231391 | Homo sapiens lactate dehydrogenase A (LDHA), mRNA [NM_005566] | -1.14 | 3.27E-02 |
| A_24_P645914 | Homo sapiens cDNA: FLJ22256 fis, clone HRC02860. [AK025909] | -1.14 | 3.06E-02 |
| A_24_P220921 | Homo sapiens calmodulin binding transcription activator 1 (CAMTA1), mRNA [NM_015215] | -1.14 | 3.15E-02 |
| A_24_P538708 | Homo sapiens cDNA FLJ42269 fis, clone TKIDN2015285. [AK124263] | -1.14 | 4.41E-02 |
| A_23_P501961 | Homo sapiens l(3)mbt-like (Drosophila) (L3MBTL), transcript variant II, mRNA [NM_032107] | -1.14 | 4.28E-02 |
| A_32_P106944 | Homo sapiens zinc finger protein 429 (ZNF429), mRNA [NM_001001415] | -1.14 | 2.16E-02 |
| A_23_P163458 | Homo sapiens EH-domain containing 4 (EHD4), mRNA [NM_139265] | -1.14 | 2.48E-02 |
| A_23_P424269 | Homo sapiens chromosome 9 open reading frame 102 (C9orf102), mRNA [NM_020207] | -1.14 | 3.88E-02 |
| A_23_P9426 | Homo sapiens golgi autoantigen, golgin subfamily a, 2 (GOLGA2), mRNA [NM_004486] | -1.14 | 4.65E-02 |
| A_23_P51051 | Homo sapiens zinc finger protein 142 (clone pHZ-49) (ZNF142), mRNA [NM_005081] | -1.14 | 2.63E-02 |
| A_24_P118271 |  | -1.14 | 1.85E-02 |
| A_24_P412238 | Homo sapiens MUS81 endonuclease homolog (yeast) (MUS81), mRNA [NM_025128] | -1.14 | 1.49E-02 |
| A_23_P86731 | Homo sapiens zinc finger protein 239 (ZNF239), mRNA [NM_005674] | -1.14 | 2.83E-02 |
| A_23_P130182 | Homo sapiens aurora kinase B (AURKB), mRNA [NM_004217] | -1.14 | 3.90E-04 |
| A_24_P942328 | Homo sapiens dihydrofolate reductase (DHFR), mRNA [NM_000791] | -1.14 | 3.07E-02 |
| A_23_P396194 | Homo sapiens ring finger and WD repeat domain 2 (RFWD2), transcript variant 1, mRNA [NM_022457] | -1.14 | 4.42E-02 |
| A_23_P80342 | Homo sapiens mitogen-activated protein kinase kinase kinase 7 interacting protein 1 (MAP3K7IP1), transcript variant alpha, mRNA [NM_006116] | -1.14 | 2.77E-02 |
| A_23_P17204 | Homo sapiens anaphase promoting complex subunit 1 (ANAPC1), mRNA [NM_022662] | -1.14 | 1.06E-03 |
| A_24_P310630 | Homo sapiens UPF3 regulator of nonsense transcripts homolog B (yeast) (UPF3B), transcript variant 1, mRNA [NM_080632] | -1.14 | 1.13E-02 |
| A_24_P195164 |  | -1.14 | 7.13E-03 |
| A_23_P78372 | Homo sapiens THO complex 1 (THOC1), mRNA [NM_005131] | -1.14 | 3.17E-02 |
| A_23_P170399 | Homo sapiens FLJ12716 protein (FLJ12716), transcript variant 1, mRNA [NM_021942] | -1.14 | 5.69E-03 |
| A_24_P338757 | Homo sapiens chromosome 13 open reading frame 22 (C13orf22), mRNA [NM_005800] | -1.14 | 4.72E-02 |
| A_23_P154070 | Homo sapiens tubulin, alpha 1 (testis specific) (TUBA1), mRNA [NM_006000] | -1.14 | 3.22E-02 |
| A_23_P127522 | Homo sapiens hydrolethalus syndrome 1 (HYLS1), mRNA [NM_145014] | -1.14 | 3.20E-02 |
| A_24_P101114 | Homo sapiens CCR4-NOT transcription complex, subunit 1 (CNOT1), transcript variant 2, mRNA [NM_206999] | -1.14 | 1.80E-02 |
| A_23_P89509 | Homo sapiens sperm associated antigen 5 (SPAG5), mRNA [NM_006461] | -1.14 | 3.29E-02 |
| A_23_P210074 | Homo sapiens zinc finger protein 514 (ZNF514), mRNA [NM_032788] | -1.14 | 2.96E-04 |
| A_23_P74115 | Homo sapiens RAD54-like (S. cerevisiae) (RAD54L), mRNA [NM_003579] | -1.14 | 5.65E-03 |
| A_24_P288890 | Homo sapiens hypothetical protein LOC144347 (LOC144347), mRNA [NM_181709] | -1.14 | 3.50E-02 |
| A_23_P80062 | Homo sapiens TAF4 RNA polymerase II, TATA box binding protein (TBP)-associated factor, 135kDa (TAF4), mRNA [NM_003185] | -1.14 | 4.32E-02 |
| A_24_P6135 | Homo sapiens l(3)mbt-like 2 (Drosophila) (L3MBTL2), transcript variant 2, mRNA [NM_001003689] | -1.14 | 3.52E-02 |
| A_24_P234415 | Homo sapiens SH3 and cysteine rich domain (STAC), mRNA [NM_003149] | -1.14 | 3.82E-02 |
| A_23_P35114 | Homo sapiens CK2 interacting protein 1; HQ0024c protein (CKIP-1), mRNA [NM_016274] | -1.14 | 4.34E-02 |
| A_24_P272313 | Homo sapiens similar to 2010300C02Rik protein (MGC42367), mRNA [NM_207362] | -1.15 | 2.93E-02 |
| A_24_P696507 | Homo sapiens cDNA FLJ35491 fis, clone SMINT2008625, moderately similar to GLYCINE CLEAVAGE SYSTEM H PROTEIN PRECURSOR. [AK092810] | -1.15 | 1.59E-02 |
| A_32_P178966 |  | -1.15 | 4.34E-02 |
| A_23_P43764 | Homo sapiens mitofusin 1 (MFN1), nuclear gene encoding mitochondrial protein, transcript variant 1, mRNA [NM_033540] | -1.15 | 3.72E-02 |
| A_24_P111912 | Homo sapiens hypothetical protein DKFZp564D172 (DKFZP564D172), mRNA [NM_032042] | -1.15 | 2.86E-02 |
| A_23_P73457 | Homo sapiens RUN and FYVE domain containing 1 (RUFY1), mRNA [NM_025158] | -1.15 | 1.65E-02 |
| A_23_P2831 | Homo sapiens endothelin receptor type B (EDNRB), transcript variant 2, mRNA [NM_003991] | -1.15 | 2.99E-02 |
| A_23_P65230 | Homo sapiens hypothetical protein FLJ14624 (FLJ14624), mRNA [NM_032813] | -1.15 | 2.92E-03 |
| A_23_P140562 | Homo sapiens proto-oncogene 8 (HCC-8), mRNA [NM_022905] | -1.15 | 2.50E-02 |
| A_32_P163469 | Homo sapiens mRNA; cDNA DKFZp686K2237 (from clone DKFZp686K2237). [AL833530] | -1.15 | 3.87E-02 |
| A_24_P270376 | Homo sapiens nuclear fragile X mental retardation protein interacting protein 1 (NUFIP1), mRNA [NM_012345] | -1.15 | 3.06E-02 |
| A_23_P90211 | Homo sapiens interferon regulatory factor 2 binding protein 1 (IRF2BP1), mRNA [NM_015649] | -1.15 | 3.14E-02 |
| A_23_P92213 | Homo sapiens abhydrolase domain containing 10 (ABHD10), mRNA [NM_018394] | -1.15 | 3.99E-02 |
| A_24_P25872 | Homo sapiens DEP domain containing 1 (DEPDC1), mRNA [NM_017779] | -1.15 | 6.30E-03 |
| A_23_P32165 | Homo sapiens LIM homeobox 2 (LHX2), mRNA [NM_004789] | -1.15 | 1.87E-02 |
| A_23_P108376 | Homo sapiens thyroid adenoma associated (THADA), transcript variant 1, mRNA [NM_022065] | -1.15 | 4.94E-02 |
| A_24_P222835 | Homo sapiens S100P binding protein Riken (S100PBPR), transcript variant 2, mRNA [NM_001017406] | -1.15 | 2.52E-02 |
| A_23_P9513 | Homo sapiens metastasis associated 1 (MTA1), mRNA [NM_004689] | -1.15 | 4.80E-02 |
| A_23_P411922 | Homo sapiens Huntingtin interacting protein C (HYPC), mRNA [NM_012272] | -1.15 | 4.77E-02 |
| A_23_P398515 | Homo sapiens protein kinase, membrane associated tyrosine/threonine 1 (PKMYT1), transcript variant 1, mRNA [NM_004203] | -1.15 | 1.24E-02 |
| A_24_P24645 | PREDICTED: Homo sapiens similar to Keratin, type I cytoskeletal 18 (Cytokeratin 18) (K18) (CK 18) (LOC132391), mRNA [XM_497978] | -1.15 | 1.47E-02 |
| A_24_P548297 | Homo sapiens full length insert cDNA clone ZB55F04. [AF086154] | -1.15 | 4.83E-02 |
| A_23_P70249 | Homo sapiens cell division cycle 25C (CDC25C), transcript variant 1, mRNA [NM_001790] | -1.15 | 1.33E-02 |
| A_24_P187448 |  | -1.15 | 4.57E-02 |
| A_23_P128060 | Homo sapiens zinc finger protein 26 (KOX 20) (ZNF26), mRNA [NM_019591] | -1.15 | 2.30E-02 |
| A_24_P221724 |  | -1.15 | 4.77E-03 |
| A_24_P226949 | Homo sapiens family with sequence similarity 29, member A (FAM29A), mRNA [NM_017645] | -1.15 | 3.19E-02 |
| A_24_P117672 | Homo sapiens serine arginine-rich pre-mRNA splicing factor SR-A1 (SR-A1), mRNA [NM_021228] | -1.15 | 2.90E-02 |
| A_23_P35219 | Homo sapiens NIMA (never in mitosis gene a)-related kinase 2 (NEK2), mRNA [NM_002497] | -1.15 | 3.49E-02 |
| A_23_P21706 | Homo sapiens CTP synthase (CTPS), mRNA [NM_001905] | -1.15 | 3.59E-02 |
| A_23_P161628 | Homo sapiens MUS81 endonuclease homolog (yeast) (MUS81), mRNA [NM_025128] | -1.15 | 4.75E-02 |
| A_23_P39336 | Homo sapiens FK506 binding protein 8, 38kDa (FKBP8), mRNA [NM_012181] | -1.15 | 4.44E-03 |
| A_23_P90612 | Homo sapiens MCM6 minichromosome maintenance deficient 6 (MIS5 homolog, S. pombe) (S. cerevisiae) (MCM6), mRNA [NM_005915] | -1.15 | 1.75E-02 |
| A_23_P404211 | Homo sapiens mRNA for KIAA1596 protein, partial cds. [AB046816] | -1.15 | 1.97E-02 |
| A_24_P6850 |  | -1.15 | 2.32E-02 |
| A_23_P24244 | Homo sapiens cDNA FLJ20360 fis, clone HEP16677. [AK000367] | -1.16 | 1.11E-02 |
| A_24_P942945 | Homo sapiens G protein-coupled receptor 126 (GPR126), mRNA [NM_198569] | -1.16 | 6.41E-03 |
| A_24_P16230 |  | -1.16 | 2.25E-02 |
| A_23_P146526 | Homo sapiens SMC5 structural maintenance of chromosomes 5-like 1 (yeast) (SMC5L1), mRNA [NM_015110] | -1.16 | 2.81E-03 |
| A_24_P306704 |  | -1.16 | 7.56E-04 |
| A_23_P17593 | Homo sapiens cadherin 4, type 1, R-cadherin (retinal) (CDH4), mRNA [NM_001794] | -1.16 | 8.54E-03 |
| A_23_P388812 | Homo sapiens hypothetical protein FLJ40629 (FLJ40629), mRNA [NM_152515] | -1.16 | 3.43E-02 |
| A_23_P259797 | Homo sapiens hypothetical protein LOC197322 (LOC197322), mRNA [NM_174917] | -1.16 | 3.92E-02 |
| A_23_P212844 | Homo sapiens transforming, acidic coiled-coil containing protein 3 (TACC3), mRNA [NM_006342] | -1.16 | 4.49E-02 |
| A_24_P203630 | Homo sapiens protein immuno-reactive with anti-PTH polyclonal antibodies (LOC400986), mRNA [NM_001010914] | -1.16 | 3.43E-02 |
| A_23_P96325 | Homo sapiens FLJ20105 protein (FLJ20105), transcript variant 2, mRNA [NM_001009954] | -1.16 | 1.04E-02 |
| A_23_P43071 | Homo sapiens MTERF domain containing 1 (MTERFD1), mRNA [NM_015942] | -1.16 | 4.66E-02 |
| A_24_P796274 |  | -1.16 | 2.69E-02 |
| A_23_P112798 | Homo sapiens cysteine-rich protein 2 (CRIP2), mRNA [NM_001312] | -1.16 | 3.47E-02 |
| A_23_P109122 | Homo sapiens mRNA for KIAA1442 protein, partial cds. [AB037863] | -1.16 | 2.64E-02 |
| A_23_P115313 | Homo sapiens torsin family 3, member A (TOR3A), mRNA [NM_022371] | -1.16 | 4.65E-02 |
| A_32_P24382 | Homo sapiens keratin associated protein 2-4, mRNA (cDNA clone MGC:74790 IMAGE:3907481), complete cds. [BC063625] | -1.16 | 7.93E-03 |
| A_24_P323664 | PREDICTED: Homo sapiens similar to Transcription factor BTF3 homolog 3 (LOC132556), mRNA [XM_067904] | -1.16 | 1.95E-02 |
| A_24_P396327 | Homo sapiens chromosome 1 open reading frame 171 (C1orf171), mRNA [NM_138467] | -1.16 | 1.56E-02 |
| A_23_P150919 | Homo sapiens cDNA FLJ14466 fis, clone MAMMA1000416. [AK027372] | -1.16 | 2.79E-02 |
| A_24_P15803 |  | -1.16 | 4.18E-02 |
| A_24_P926125 | Homo sapiens similar to protein phosphatase 2A 48 kDa regulatory subunit isoform 1; serine/threonine protein phosphatase 2A, 48kDa regulatory subunit; PP2A, subunit B, PR48 isoform; PP2A B subunit PR48; NY-REN-8 antigen, mRNA (cDNA clone... | -1.16 | 3.72E-02 |
| A_23_P502915 | Homo sapiens WD repeat domain 1 (WDR1), transcript variant 1, mRNA [NM_017491] | -1.16 | 1.30E-02 |
| A_23_P101246 | Homo sapiens, clone IMAGE:4401841, mRNA. [BC016993] | -1.16 | 4.19E-02 |
| A_23_P124417 | Homo sapiens BUB1 budding uninhibited by benzimidazoles 1 homolog (yeast) (BUB1), mRNA [NM_004336] | -1.16 | 1.03E-02 |
| A_23_P163858 | Homo sapiens zinc and ring finger 1 (ZNRF1), mRNA [NM_032268] | -1.16 | 9.29E-03 |
| A_32_P27917 | Homo sapiens kinesin family member 26A, mRNA (cDNA clone MGC:14884 IMAGE:3502885), complete cds. [BC009415] | -1.16 | 4.74E-02 |
| A_23_P162466 | Homo sapiens plakophilin 2 (PKP2), transcript variant 2b, mRNA [NM_004572] | -1.16 | 4.70E-02 |
| A_23_P67399 | Homo sapiens striatin, calmodulin binding protein 4 (STRN4), mRNA [NM_013403] | -1.16 | 6.58E-03 |
| A_24_P153003 |  | -1.16 | 2.04E-02 |
| A_23_P30495 | Homo sapiens 3-hydroxy-3-methylglutaryl-Coenzyme A reductase (HMGCR), mRNA [NM_000859] | -1.16 | 5.18E-03 |
| A_23_P371129 | Homo sapiens BTB (POZ) domain containing 12 (BTBD12), mRNA [NM_032444] | -1.16 | 1.56E-02 |
| A_24_P181944 | Homo sapiens PHD finger protein 20 (PHF20), mRNA [NM_016436] | -1.16 | 2.91E-02 |
| A_24_P93798 | Homo sapiens RIO kinase 1 (yeast) (RIOK1), transcript variant 1, mRNA [NM_031480] | -1.16 | 2.32E-02 |
| A_23_P67127 | Homo sapiens hypothetical protein FLJ90805 (FLJ90805), mRNA [NM_173633] | -1.16 | 3.52E-02 |
| A_24_P161809 |  | -1.16 | 1.42E-02 |
| A_23_P61268 | Homo sapiens brain protein 16 (LOC51236), mRNA [NM_016458] | -1.16 | 3.10E-02 |
| A_24_P186746 |  | -1.16 | 1.85E-02 |
| A_24_P256063 |  | -1.17 | 6.61E-03 |
| A_23_P134454 | Homo sapiens caveolin 1, caveolae protein, 22kDa (CAV1), mRNA [NM_001753] | -1.17 | 3.78E-02 |
| A_23_P385861 | Homo sapiens cell division cycle associated 2 (CDCA2), mRNA [NM_152562] | -1.17 | 4.61E-02 |
| A_23_P128641 | Homo sapiens chromosome 13 open reading frame 22 (C13orf22), mRNA [NM_005800] | -1.17 | 1.22E-02 |
| A_24_P929974 |  | -1.17 | 3.50E-02 |
| A_23_P52219 | Homo sapiens SPFH domain family, member 1 (SPFH1), mRNA [NM_006459] | -1.17 | 2.85E-02 |
| A_24_P392842 |  | -1.17 | 2.51E-02 |
| A_23_P122775 | Homo sapiens reticulon 4 interacting protein 1 (RTN4IP1), nuclear gene encoding mitochondrial protein, mRNA [NM_032730] | -1.17 | 2.89E-02 |
| A_23_P428326 | Homo sapiens cDNA FLJ20748 fis, clone HEP05772. [AK000755] | -1.17 | 4.24E-02 |
| A_24_P331704 | Homo sapiens hypothetical protein LOC144501 (LOC144501), mRNA [NM_182507] | -1.17 | 3.58E-02 |
| A_23_P55682 | Homo sapiens zinc finger protein 447 (ZNF447), mRNA [NM_023926] | -1.17 | 3.17E-02 |
| A_23_P79794 | Homo sapiens TGFB-induced factor 2 (TALE family homeobox) (TGIF2), mRNA [NM_021809] | -1.17 | 2.76E-02 |
| A_24_P161733 |  | -1.17 | 4.21E-02 |
| A_32_P6274 |  | -1.17 | 8.40E-03 |
| A_24_P350136 |  | -1.17 | 2.91E-02 |
| A_23_P90333 | PREDICTED: Homo sapiens zinc finger protein 404 (ZNF404), mRNA [XM_292765] | -1.17 | 1.08E-02 |
| A_23_P63402 | Homo sapiens G-protein signalling modulator 2 (AGS3-like, C. elegans) (GPSM2), mRNA [NM_013296] | -1.17 | 1.87E-02 |
| A_23_P98015 | Homo sapiens cutC copper transporter homolog (E.coli) (CUTC), mRNA [NM_015960] | -1.17 | 1.56E-02 |
| A_23_P416468 | Homo sapiens DNA helicase homolog (PIF1) mRNA, partial cds. [AF108138] | -1.17 | 3.01E-02 |
| A_24_P89887 | Homo sapiens chromosome 9 open reading frame 3 (C9orf3), mRNA [NM_032823] | -1.17 | 1.83E-02 |
| A_23_P82000 | Homo sapiens TEA domain family member 3 (TEAD3), mRNA [NM_003214] | -1.17 | 3.56E-02 |
| A_24_P69691 | Homo sapiens zinc finger protein 25 (KOX 19) (ZNF25), mRNA [NM_145011] | -1.17 | 3.46E-02 |
| A_32_P125820 |  | -1.17 | 2.01E-02 |
| A_24_P551028 | Homo sapiens hypothetical protein LOC339745 (LOC339745), mRNA [NM_001001664] | -1.17 | 1.55E-02 |
| A_24_P12626 | Homo sapiens caveolin 1, caveolae protein, 22kDa (CAV1), mRNA [NM_001753] | -1.17 | 4.94E-02 |
| A_24_P375360 |  | -1.18 | 1.13E-02 |
| A_23_P88194 | Homo sapiens survival of motor neuron protein interacting protein 1 (SIP1), transcript variant alpha, mRNA [NM_003616] | -1.18 | 1.55E-03 |
| A_24_P418687 |  | -1.18 | 2.70E-02 |
| A_23_P259090 | Homo sapiens nudix (nucleoside diphosphate linked moiety X)-type motif 12 (NUDT12), mRNA [NM_031438] | -1.18 | 2.45E-02 |
| A_32_P109522 | Homo sapiens chromosome 6 open reading frame 113 (C6orf113), mRNA [NM_145062] | -1.18 | 3.90E-02 |
| A_23_P390384 | Homo sapiens, clone IMAGE:5259432, mRNA. [BC037316] | -1.18 | 4.48E-02 |
| A_24_P584463 |  | -1.18 | 1.02E-02 |
| A_23_P104705 | Homo sapiens solute carrier family 29 (nucleoside transporters), member 2 (SLC29A2), mRNA [NM_001532] | -1.18 | 2.77E-02 |
| A_23_P250564 | Homo sapiens protein kinase C, epsilon (PRKCE), mRNA [NM_005400] | -1.18 | 3.73E-02 |
| A_24_P222997 | Homo sapiens zinc finger, RAN-binding domain containing 3 (ZRANB3), mRNA [NM_032143] | -1.18 | 2.35E-02 |
| A_23_P373708 | Homo sapiens hypothetical protein FLJ40504 (FLJ40504), mRNA [NM_173624] | -1.18 | 3.17E-02 |
| A_24_P876772 | Homo sapiens cDNA clone MGC:40288 IMAGE:5169056, complete cds. [BC032332] | -1.18 | 1.79E-02 |
| A_23_P217120 | Homo sapiens euchromatic histone-lysine N-methyltransferase 1 (EHMT1), mRNA [NM_024757] | -1.18 | 5.80E-03 |
| A_24_P317835 | Homo sapiens inositol polyphosphate-5-phosphatase, 72 kDa (INPP5E), mRNA [NM_019892] | -1.18 | 4.37E-02 |
| A_23_P58321 | Homo sapiens cyclin A2 (CCNA2), mRNA [NM_001237] | -1.18 | 6.84E-04 |
| A_24_P792988 |  | -1.18 | 3.94E-03 |
| A_24_P493116 |  | -1.18 | 4.86E-02 |
| A_24_P477102 | PREDICTED: Homo sapiens similar to FLJ10101 protein (LOC284269), mRNA [XM_209097] | -1.18 | 1.47E-02 |
| A_24_P145316 | Homo sapiens dystrobrevin binding protein 1 (DTNBP1), transcript variant 2, mRNA [NM_183040] | -1.18 | 4.61E-02 |
| A_23_P98884 | Homo sapiens ring finger protein 41 (RNF41), transcript variant 2, mRNA [NM_194358] | -1.18 | 3.34E-02 |
| A_24_P103886 | Homo sapiens isopentenyl-diphosphate delta isomerase 1 (IDI1), mRNA [NM_004508] | -1.18 | 2.37E-02 |
| A_32_P733356 | Homo sapiens cDNA: FLJ22714 fis, clone HSI13646. [AK026367] | -1.18 | 2.68E-02 |
| A_24_P401601 |  | -1.18 | 1.78E-02 |
| A_24_P264549 |  | -1.18 | 5.96E-03 |
| A_24_P179467 | Homo sapiens solute carrier family 1 (high affinity aspartate/glutamate transporter), member 6 (SLC1A6), mRNA [NM_005071] | -1.18 | 1.95E-02 |
| A_24_P176493 | Homo sapiens ATM/ATR-Substrate Chk2-Interacting Zn2+-finger protein (ASCIZ), mRNA [NM_015251] | -1.18 | 4.50E-02 |
| A_32_P28685 | Homo sapiens small nuclear ribonucleoprotein polypeptide A' (SNRPA1), mRNA [NM_003090] | -1.18 | 6.50E-04 |
| A_23_P20683 | Homo sapiens KIAA0020 (KIAA0020), mRNA [NM_014878] | -1.18 | 4.77E-02 |
| A_24_P754086 | Homo sapiens nucleolin (NCL), mRNA [NM_005381] | -1.18 | 2.11E-02 |
| A_23_P317800 | Homo sapiens anaphase promoting complex subunit 4 (ANAPC4), mRNA [NM_013367] | -1.18 | 2.84E-02 |
| A_23_P35617 | Homo sapiens phospholipase C, epsilon 1 (PLCE1), mRNA [NM_016341] | -1.19 | 9.38E-04 |
| A_24_P83678 | Homo sapiens chromosome 6 open reading frame 167 (C6orf167), mRNA [NM_198468] | -1.19 | 3.35E-02 |
| A_24_P281374 | PREDICTED: Homo sapiens similar to Keratin, type I cytoskeletal 18 (Cytokeratin 18) (K18) (CK 18) (LOC442406), mRNA [XM_498307] | -1.19 | 5.29E-03 |
| A_32_P718498 | Homo sapiens myeloid/lymphoid or mixed-lineage leukemia (trithorax homolog, Drosophila); translocated to, 6 (MLLT6), mRNA [NM_005937] | -1.19 | 4.33E-02 |
| A_32_P89709 | Homo sapiens tropomyosin 1 (alpha) (TPM1), transcript variant 3, mRNA [NM_001018004] | -1.19 | 4.70E-02 |
| A_24_P478726 | Homo sapiens similar to RIKEN cDNA 2210021J22, mRNA (cDNA clone MGC:87534 IMAGE:30338205), complete cds. [BC067871] | -1.19 | 3.36E-03 |
| A_23_P415443 | Homo sapiens barren homolog (Drosophila) (BRRN1), mRNA [NM_015341] | -1.19 | 4.96E-02 |
| A_23_P214156 | Homo sapiens SUMO1/sentrin specific protease 6 (SENP6), mRNA [NM_015571] | -1.19 | 2.00E-02 |
| A_32_P97169 | Homo sapiens mRNA; cDNA DKFZp686H20120 (from clone DKFZp686H20120). [BX640888] | -1.19 | 2.25E-02 |
| A_24_P419132 | Homo sapiens FSH primary response (LRPR1 homolog, rat) 1 (FSHPRH1), mRNA [NM_006733] | -1.19 | 1.51E-02 |
| A_24_P109661 |  | -1.19 | 4.38E-02 |
| A_23_P77993 | Homo sapiens complement component 1, q subcomponent-like 1 (C1QL1), mRNA [NM_006688] | -1.19 | 4.04E-02 |
| A_24_P34505 | Homo sapiens hypothetical LOC79954 (FLJ14075), mRNA [NM_024894] | -1.19 | 2.49E-03 |
| A_24_P42136 | Homo sapiens keratin 18 (KRT18), transcript variant 1, mRNA [NM_000224] | -1.19 | 3.33E-02 |
| A_23_P413803 | Homo sapiens hypothetical protein FLJ35779 (FLJ35779), mRNA [NM_152408] | -1.19 | 1.20E-02 |
| A_24_P99090 | Homo sapiens cytoskeleton associated protein 2 (CKAP2), mRNA [NM_018204] | -1.19 | 4.90E-02 |
| A_23_P211212 | Homo sapiens collagen, type XVIII, alpha 1 (COL18A1), transcript variant 1, mRNA [NM_030582] | -1.19 | 9.64E-03 |
| A_23_P163148 | Homo sapiens chromosome 14 open reading frame 133 (C14orf133), mRNA [NM_022067] | -1.19 | 5.86E-04 |
| A_23_P41327 | Homo sapiens hypothetical protein FLJ20425 (LYAR), mRNA [NM_017816] | -1.19 | 4.07E-02 |
| A_32_P62769 | Homo sapiens cDNA FLJ34465 fis, clone HLUNG2003061. [AK091784] | -1.19 | 2.60E-02 |
| A_23_P14072 | Homo sapiens keratin 8 (KRT8), mRNA [NM_002273] | -1.19 | 4.92E-03 |
| A_24_P92744 |  | -1.19 | 4.90E-02 |
| A_24_P247454 |  | -1.19 | 4.13E-02 |
| A_23_P356484 | Homo sapiens ribosomal protein S10 (RPS10), mRNA [NM_001014] | -1.19 | 3.42E-02 |
| A_23_P210581 | Homo sapiens potassium voltage-gated channel, subfamily G, member 1 (KCNG1), transcript variant 1, mRNA [NM_002237] | -1.20 | 3.13E-02 |
| A_23_P104651 | Homo sapiens cell division cycle associated 5 (CDCA5), mRNA [NM_080668] | -1.20 | 1.93E-02 |
| A_32_P96036 | Q69Z36 (Q69Z36) MKIAA2009 protein (Fragment), partial (8%) [THC2430293] | -1.20 | 2.66E-02 |
| A_23_P40059 | Homo sapiens PMS1 postmeiotic segregation increased 1 (S. cerevisiae) (PMS1), mRNA [NM_000534] | -1.20 | 8.85E-04 |
| A_23_P77321 | Homo sapiens KIAA0252 (KIAA0252), mRNA [NM_015138] | -1.20 | 3.38E-02 |
| A_23_P351232 | Homo sapiens hypothetical protein MGC33584 (MGC33584), mRNA [NM_173680] | -1.20 | 4.53E-02 |
| A_23_P211659 | Homo sapiens ceramide kinase (CERK), transcript variant 1, mRNA [NM_022766] | -1.20 | 1.66E-02 |
| A_23_P384056 | Homo sapiens coiled-coil domain containing 14 (CCDC14), mRNA [NM_022757] | -1.20 | 2.35E-03 |
| A_23_P367676 | Homo sapiens SIN3 homolog A, transcription regulator (yeast) (SIN3A), mRNA [NM_015477] | -1.20 | 3.58E-02 |
| A_24_P227585 | Homo sapiens KIAA1704 (KIAA1704), mRNA [NM_018559] | -1.20 | 4.14E-02 |
| A_23_P396981 | Homo sapiens hypothetical protein LOC285331 (LOC285331), mRNA [NM_001012506] | -1.20 | 2.14E-02 |
| A_24_P471242 |  | -1.20 | 8.92E-03 |
| A_23_P2537 | Homo sapiens methylmalonic aciduria (cobalamin deficiency) cblB type (MMAB), mRNA [NM_052845] | -1.20 | 1.23E-02 |
| A_24_P75879 | Homo sapiens cDNA clone IMAGE:30334866. [BC092503] | -1.20 | 3.05E-02 |
| A_23_P23894 | Homo sapiens receptor interacting protein kinase 5 (RIPK5), transcript variant 1, mRNA [NM_015375] | -1.20 | 3.17E-02 |
| A_23_P12733 | Homo sapiens H2A histone family, member Y2 (H2AFY2), mRNA [NM_018649] | -1.20 | 1.99E-02 |
| A_24_P337657 | Homo sapiens serum response factor (c-fos serum response element-binding transcription factor) (SRF), mRNA [NM_003131] | -1.20 | 7.45E-03 |
| A_32_P19966 | Homo sapiens cDNA FLJ45029 fis, clone BRAWH3018326. [AK126976] | -1.20 | 2.75E-02 |
| A_24_P194954 |  | -1.20 | 1.66E-02 |
| A_24_P161827 |  | -1.20 | 1.86E-02 |
| A_23_P250735 | Homo sapiens chromobox homolog 7 (CBX7), mRNA [NM_175709] | -1.20 | 2.91E-02 |
| A_24_P366656 | Homo sapiens SH3 domain protein D19 (SH3D19), mRNA [NM_001009555] | -1.21 | 3.70E-02 |
| A_24_P857404 | Homo sapiens cDNA FLJ43493 fis, clone OCBBF3009279. [AK125482] | -1.21 | 1.99E-02 |
| A_32_P396186 | Homo sapiens cDNA FLJ10046 fis, clone HEMBA1001133. [AK000908] | -1.21 | 4.48E-02 |
| A_24_P652700 | Homo sapiens mRNA; cDNA DKFZp686C15165 (from clone DKFZp686C15165). [BX648822] | -1.21 | 1.35E-02 |
| A_24_P225970 | Homo sapiens shugoshin-like 1 (S. pombe) (SGOL1), transcript variant A1, mRNA [NM_001012409] | -1.21 | 2.84E-02 |
| A_23_P259586 | Homo sapiens TTK protein kinase (TTK), mRNA [NM_003318] | -1.21 | 4.92E-02 |
| A_23_P42575 | Homo sapiens caldesmon 1 (CALD1), transcript variant 1, mRNA [NM_033138] | -1.21 | 1.72E-03 |
| A_32_P109296 | Homo sapiens leucine-rich repeat kinase 1 (MGC45866), mRNA [NM_152259] | -1.21 | 3.26E-02 |
| A_32_P183218 | Homo sapiens cDNA FLJ33970 fis, clone DFNES2001564. [AK091289] | -1.21 | 1.98E-02 |
| A_24_P137545 | Homo sapiens BH3-only member B protein (BOMB), mRNA [NM_024949] | -1.21 | 3.07E-03 |
| A_23_P48835 | Homo sapiens kinesin family member 23 (KIF23), transcript variant 1, mRNA [NM_138555] | -1.21 | 1.95E-02 |
| A_24_P350060 | PREDICTED: Homo sapiens similar to Keratin, type I cytoskeletal 18 (Cytokeratin 18) (K18) (CK 18) (LOC391819), mRNA [XM_498013] | -1.21 | 8.86E-03 |
| A_23_P111995 | Homo sapiens lysyl oxidase-like 2 (LOXL2), mRNA [NM_002318] | -1.21 | 4.62E-02 |
| A_24_P53985 | Homo sapiens zinc finger, MYM domain containing 1 (ZMYM1), mRNA [NM_024772] | -1.21 | 4.25E-02 |
| A_32_P51518 | Homo sapiens cDNA FLJ40901 fis, clone UTERU2003704. [AK098220] | -1.21 | 2.79E-02 |
| A_23_P27315 | Homo sapiens elastin microfibril interfacer 2 (EMILIN2), mRNA [NM_032048] | -1.21 | 4.11E-02 |
| A_24_P8088 | Homo sapiens RIO kinase 1 (yeast) (RIOK1), transcript variant 2, mRNA [NM_153005] | -1.22 | 1.77E-02 |
| A_24_P837234 | PREDICTED: Homo sapiens similar to ribosomal protein S2 (LOC442426), mRNA [XM_498332] | -1.22 | 3.92E-02 |
| A_23_P60002 | Homo sapiens KIAA0103 (KIAA0103), mRNA [NM_014673] | -1.22 | 1.66E-03 |
| A_23_P99172 | Homo sapiens hypothetical protein MGC13183 (MGC13183), mRNA [NM_032358] | -1.22 | 2.49E-02 |
| A_23_P155711 | Homo sapiens nei endonuclease VIII-like 3 (E. coli) (NEIL3), mRNA [NM_018248] | -1.22 | 2.01E-02 |
| A_24_P248863 | Homo sapiens DHHC domain-containing zinc finger protein mRNA, complete cds. [AY629351] | -1.22 | 3.72E-02 |
| A_24_P66522 | Homo sapiens 5-azacytidine induced 1 (AZI1), transcript variant 1, mRNA [NM_014984] | -1.22 | 1.92E-02 |
| A_23_P60753 |  | -1.22 | 4.38E-02 |
| A_23_P112241 | Homo sapiens DnaJ (Hsp40) homolog, subfamily B, member 5 (DNAJB5), mRNA [NM_012266] | -1.22 | 4.11E-02 |
| A_23_P205228 | Homo sapiens ATPase, Cu++ transporting, beta polypeptide (Wilson disease) (ATP7B), transcript variant 1, mRNA [NM_000053] | -1.22 | 1.98E-02 |
| A_24_P27412 | Homo sapiens RNA, U transporter 1 (RNUT1), mRNA [NM_005701] | -1.22 | 1.09E-02 |
| A_23_P160460 | Homo sapiens UDP-N-acteylglucosamine pyrophosphorylase 1 (UAP1), mRNA [NM_003115] | -1.22 | 4.78E-02 |
| A_24_P383660 |  | -1.22 | 2.68E-02 |
| A_23_P94546 | Homo sapiens G kinase anchoring protein 1 (GKAP1), mRNA [NM_025211] | -1.22 | 8.36E-03 |
| A_23_P140450 | Homo sapiens solute carrier family 27 (fatty acid transporter), member 2 (SLC27A2), mRNA [NM_003645] | -1.22 | 1.14E-02 |
| A_23_P333420 | Homo sapiens Ran GTPase activating protein 1 (RANGAP1), mRNA [NM_002883] | -1.22 | 4.33E-02 |
| A_23_P74349 | Homo sapiens cell division cycle associated 1 (CDCA1), transcript variant 1, mRNA [NM_145697] | -1.22 | 6.50E-03 |
| A_23_P334635 | Homo sapiens jerky homolog (mouse) (JRK), mRNA [NM_003724] | -1.23 | 1.98E-02 |
| A_32_P128661 | Homo sapiens cDNA clone IMAGE:4500064, partial cds. [BC023274] | -1.23 | 4.41E-02 |
| A_24_P381604 | Homo sapiens integral membrane protein 2B (ITM2B), mRNA [NM_021999] | -1.23 | 4.67E-02 |
| A_23_P375 | Homo sapiens cell division cycle associated 8 (CDCA8), mRNA [NM_018101] | -1.23 | 1.18E-02 |
| A_23_P308731 | Homo sapiens rhomboid, veinlet-like 4 (Drosophila) (RHBDL4), mRNA [NM_138328] | -1.23 | 3.41E-02 |
| A_32_P123966 | Homo sapiens KIAA1005 protein (KIAA1005), mRNA [NM_015272] | -1.23 | 3.33E-03 |
| A_23_P212383 | Homo sapiens SAC1 suppressor of actin mutations 1-like (yeast) (SACM1L), mRNA [NM_014016] | -1.23 | 2.72E-02 |
| A_23_P204751 | Homo sapiens amiloride-sensitive cation channel 2, neuronal (ACCN2), transcript variant 1, mRNA [NM_020039] | -1.23 | 1.21E-03 |
| A_32_P155091 | Homo sapiens ataxin 2-like (ATXN2L), transcript variant B, mRNA [NM_145714] | -1.23 | 3.69E-02 |
| A_23_P165927 | Homo sapiens stathmin-like 3 (STMN3), mRNA [NM_015894] | -1.23 | 2.95E-02 |
| A_32_P19887 | Homo sapiens methyltransferase 5 domain containing 1 (METT5D1), mRNA [NM_152636] | -1.23 | 2.17E-02 |
| A_23_P140705 | Homo sapiens chromosome 15 open reading frame 23, mRNA (cDNA clone IMAGE:3952251), partial cds. [BC004543] | -1.24 | 1.33E-02 |
| A_24_P332595 |  | -1.24 | 1.44E-02 |
| A_24_P409420 |  | -1.24 | 1.30E-02 |
| A_24_P238257 | Homo sapiens cDNA FLJ35848 fis, clone TESTI2006894. [AK093167] | -1.24 | 2.12E-02 |
| A_23_P215070 | Homo sapiens testis specific, 14 (TSGA14), mRNA [NM_018718] | -1.24 | 2.70E-02 |
| A_23_P99604 | Homo sapiens KIAA1333 (KIAA1333), mRNA [NM_017769] | -1.24 | 2.67E-02 |
| A_24_P304439 | Homo sapiens serine dehydratase (SDS), mRNA [NM_006843] | -1.24 | 3.73E-02 |
| A_23_P136805 | Homo sapiens Rho GTPase activating protein 11A (ARHGAP11A), mRNA [NM_014783] | -1.24 | 2.77E-02 |
| A_23_P55256 | Homo sapiens zinc finger protein 652 (ZNF652), mRNA [NM_014897] | -1.24 | 2.53E-02 |
| A_23_P53856 | Homo sapiens phosphonoformate immuno-associated protein 5 (PFAAP5), mRNA [NM_014887] | -1.25 | 3.70E-02 |
| A_24_P349151 | Homo sapiens spindle assembly abnormal protein 6 (SAS-6), mRNA [NM_194292] | -1.25 | 4.52E-02 |
| A_23_P334218 | Homo sapiens WD repeat domain 67 (WDR67), mRNA [NM_145647] | -1.25 | 1.58E-03 |
| A_23_P50108 | Homo sapiens kinetochore associated 2 (KNTC2), mRNA [NM_006101] | -1.25 | 1.42E-02 |
| A_23_P52362 | Homo sapiens solute carrier family 18 (vesicular acetylcholine), member 3 (SLC18A3), mRNA [NM_003055] | -1.25 | 1.19E-02 |
| A_23_P344000 | Homo sapiens beta1,4-N-acetylgalactosaminyltransferases IV (Beta4GalNAc-T4), mRNA [NM_178537] | -1.25 | 3.29E-02 |
| A_24_P41979 |  | -1.25 | 2.07E-02 |
| A_23_P97853 | Homo sapiens chromosome 10 open reading frame 57 (C10orf57), mRNA [NM_025125] | -1.25 | 1.87E-02 |
| A_32_P199252 | Homo sapiens heat shock 90kDa protein 1, alpha (HSPCA), transcript variant 2, mRNA [NM_005348] | -1.26 | 1.86E-02 |
| A_23_P129466 | Homo sapiens activating transcription factor 7 interacting protein 2 (ATF7IP2), mRNA [NM_024997] | -1.26 | 1.05E-03 |
| A_23_P32707 | Homo sapiens extra spindle poles like 1 (S. cerevisiae) (ESPL1), mRNA [NM_012291] | -1.26 | 1.61E-03 |
| A_24_P255954 |  | -1.26 | 1.92E-02 |
| A_24_P189112 | Homo sapiens chromosome 6 open reading frame 182 (C6orf182), mRNA [NM_173830] | -1.26 | 5.16E-03 |
| A_23_P381577 | Homo sapiens zinc finger protein 25 (KOX 19) (ZNF25), mRNA [NM_145011] | -1.26 | 3.32E-02 |
| A_24_P322635 | Homo sapiens engulfment and cell motility 2 (ced-12 homolog, C. elegans) (ELMO2), transcript variant 3, mRNA [NM_182764] | -1.27 | 2.28E-02 |
| A_23_P116682 | Homo sapiens SWI/SNF related, matrix associated, actin dependent regulator of chromatin, subfamily c, member 2 (SMARCC2), transcript variant 2, mRNA [NM_139067] | -1.27 | 3.17E-02 |
| A_32_P151800 | Homo sapiens family with sequence similarity 72, member A (FAM72A), mRNA [NM_207418] | -1.28 | 6.44E-03 |
| A_24_P169843 |  | -1.28 | 1.41E-03 |
| A_23_P160518 | Homo sapiens tripartite motif-containing 45 (TRIM45), mRNA [NM_025188] | -1.29 | 1.53E-02 |
| A_23_P114903 | Homo sapiens heat shock 70kDa protein 6 (HSP70B') (HSPA6), mRNA [NM_002155] | -1.29 | 2.78E-02 |
| A_24_P783679 |  | -1.29 | 4.06E-02 |
| A_24_P359856 | Homo sapiens histone deacetylase 4 (HDAC4), mRNA [NM_006037] | -1.29 | 2.49E-03 |
| A_23_P59358 | Homo sapiens chromosome 6 open reading frame 182 (C6orf182), mRNA [NM_173830] | -1.30 | 8.07E-03 |
| A_24_P31627 | Homo sapiens potassium voltage-gated channel, Shab-related subfamily, member 1 (KCNB1), mRNA [NM_004975] | -1.30 | 4.13E-02 |
| A_24_P686014 |  | -1.30 | 2.10E-02 |
| A_24_P104980 | Homo sapiens germline mRNA for immunoglobulin lambda-2 chain constant region, Daudi cell line. [AJ319669] | -1.31 | 3.27E-02 |
| A_32_P218707 | PREDICTED: Homo sapiens similar to CDNA sequence BC012256 (LOC400969), mRNA [XM_379108] | -1.31 | 3.07E-02 |
| A_23_P411335 | Homo sapiens shugoshin-like 2 (S. pombe) (SGOL2), mRNA [NM_152524] | -1.31 | 2.84E-03 |
| A_23_P100141 | Homo sapiens chromosome 16 open reading frame 28 (C16orf28), mRNA [NM_023076] | -1.32 | 2.59E-03 |
| A_23_P150935 | Homo sapiens trophinin associated protein (tastin) (TROAP), mRNA [NM_005480] | -1.32 | 1.14E-02 |
| A_24_P63522 | Homo sapiens 3-hydroxy-3-methylglutaryl-Coenzyme A synthase 1 (soluble) (HMGCS1), mRNA [NM_002130] | -1.32 | 2.66E-02 |
| A_23_P252664 | Homo sapiens fucosyltransferase 6 (alpha (1,3) fucosyltransferase) (FUT6), mRNA [NM_000150] | -1.32 | 3.99E-02 |
| A_24_P230466 |  | -1.32 | 7.85E-03 |
| A_23_P83328 | Homo sapiens endoglin (Osler-Rendu-Weber syndrome 1) (ENG), mRNA [NM_000118] | -1.33 | 4.57E-02 |
| A_24_P80204 | Homo sapiens BENE protein (BENE), mRNA [NM_005434] | -1.33 | 3.36E-02 |
| A_23_P435183 | Homo sapiens LRR FLI-I interacting protein 1 (LRRFIP1) mRNA, partial cds. [AF115510] | -1.33 | 2.96E-02 |
| A_23_P6561 | Homo sapiens hypothetical protein FLJ10213 (FLJ10213), mRNA [NM_018029] | -1.33 | 1.54E-02 |
| A_24_P250666 | Homo sapiens surfactant, pulmonary-associated protein C (SFTPC), mRNA [NM_003018] | -1.34 | 3.97E-02 |
| A_32_P215143 | Q6PIE2 (Q6PIE2) MGC9515 protein, partial (6%) [THC2306274] | -1.34 | 3.48E-02 |
| A_23_P216517 | Homo sapiens chromosome 9 open reading frame 100 (C9orf100), mRNA [NM_032818] | -1.35 | 2.14E-03 |
| A_32_P134580 | Homo sapiens high-mobility group box 1 (HMGB1), mRNA [NM_002128] | -1.35 | 3.47E-03 |
| A_23_P38876 | Homo sapiens lipase, hormone-sensitive (LIPE), mRNA [NM_005357] | -1.35 | 2.67E-02 |
| A_24_P675947 | PREDICTED: Homo sapiens similar to ribosomal protein S3a; 40S ribosomal protein S3a; v-fos transformation effector protein 1 (LOC391706), mRNA [XM_497979] | -1.35 | 1.30E-02 |
| A_23_P68807 | Homo sapiens, clone IMAGE:4994346, mRNA. [BC021857] | -1.35 | 1.47E-02 |
| A_24_P649735 | Q6PJX0 (Q6PJX0) MADP-1 protein (Fragment), partial (64%) [THC2281176] | -1.36 | 4.15E-02 |
| A_23_P110851 | Homo sapiens telomerase reverse transcriptase (TERT), transcript variant 1, mRNA [NM_003219] | -1.36 | 1.54E-02 |
| A_23_P53530 | Homo sapiens MTERF domain containing 3 (MTERFD3), mRNA [NM_025198] | -1.37 | 3.53E-03 |
| A_23_P95213 | Homo sapiens surfactant, pulmonary-associated protein C, mRNA (cDNA clone MGC:14509 IMAGE:4043169), complete cds. [BC005913] | -1.37 | 2.55E-02 |
| A_23_P360605 | Homo sapiens KIAA0802, mRNA (cDNA clone MGC:39663 IMAGE:5268201), complete cds. [BC040542] | -1.37 | 1.01E-02 |
| A_24_P286114 | Homo sapiens solute carrier family 1 (glial high affinity glutamate transporter), member 3 (SLC1A3), mRNA [NM_004172] | -1.39 | 6.14E-04 |
| A_23_P88848 | Homo sapiens dihydrouridine synthase 2-like (SMM1, S. cerevisiae) (DUS2L), mRNA [NM_017803] | -1.42 | 1.30E-02 |
| A_23_P21143 | Homo sapiens mRNA; cDNA DKFZp686B0790 (from clone DKFZp686B0790); complete cds. [BX538238] | -1.46 | 8.32E-03 |
| A_32_P189034 | BC000698 keratin 18 [Homo sapiens;], partial (19%) [THC2310027] | -1.49 | 8.34E-03 |
| A_23_P206901 | Homo sapiens nudE nuclear distribution gene E homolog 1 (A. nidulans) (NDE1), mRNA [NM_017668] | -1.50 | 2.98E-02 |
| A_24_P873659 | Homo sapiens clone alpha1 mRNA sequence. [AF001540] | -1.70 | 2.51E-02 |
