# Supplementary material for: Functional genomics of human bronchial epithelial cells directly interacting with conidia of Aspergillus fumigatus
Source: BMC Genomics. 2010 Jun 4;11:358. doi: 10.1186/1471-2164-11-358 (PMC2897809; doi:10.1186/1471-2164-11-358)
Supplement: Additional file 2 — Supplementary Table S2: Gene Set Enrichment Analysis (GSEA). 177 gene sets were identified as sharing up-regulated genes with our data and 54 gene sets were identified as sharing down-regulated genes (NOM p-value ≤ 0.05 and FDR q-value ≤ 0.25). [file 1471-2164-11-358-S2.DOC]

**Supplementary Table 2: Gene Set Enrichment Analysis (GSEA).** 177 gene sets were identified as sharing up-regulated genes with our data and 54 gene sets were identified as sharing down-regulated genes (NOM p-value ≤ 0.05 and FDR q-value ≤ 0.25).

| **Gene Set (Up-Regulated)** | **Size** | **ES** | **NES** | **NOM p-val** | **FDR q-val** | **FWER p-val** | **Rank at Max** | **Leading Edge** |
| --- | --- | --- | --- | --- | --- | --- | --- | --- |
| CARIES_PULP_UP | 195 | 0.542 | 2.375 | 0 | 0.001 | 0.001 | 3690 | tags=52%, list=21%, signal=66% |
| CARIES_PULP_HIGH_UP | 87 | 0.56 | 2.208 | 0 | 0.003 | 0.006 | 3878 | tags=57%, list=22%, signal=73% |
| TAKEDA_NUP8_HOXA9_3D_UP | 177 | 0.493 | 2.137 | 0 | 0.005 | 0.016 | 2571 | tags=36%, list=15%, signal=41% |
| ERYTHPATHWAY | 15 | 0.8 | 2.126 | 0 | 0.005 | 0.019 | 1517 | tags=67%, list=9%, signal=73% |
| LINDSTEDT_DEND_8H_VS_48H_UP | 61 | 0.562 | 2.071 | 0 | 0.01 | 0.046 | 3773 | tags=56%, list=22%, signal=71% |
| CMV_HCMV_TIMECOURSE_12HRS_UP | 25 | 0.65 | 2.051 | 0 | 0.012 | 0.07 | 3281 | tags=60%, list=19%, signal=74% |
| TSA_CD4_DN | 17 | 0.719 | 2 | 0 | 0.023 | 0.145 | 2186 | tags=53%, list=12%, signal=60% |
| TAKEDA_NUP8_HOXA9_10D_UP | 156 | 0.459 | 1.985 | 0 | 0.026 | 0.183 | 2005 | tags=27%, list=11%, signal=30% |
| SANA_TNFA_ENDOTHELIAL_UP | 77 | 0.508 | 1.94 | 0 | 0.032 | 0.306 | 3315 | tags=40%, list=19%, signal=49% |
| CROONQUIST_IL6_RAS_UP | 23 | 0.645 | 1.941 | 0 | 0.035 | 0.304 | 4406 | tags=70%, list=25%, signal=93% |
| UVB_NHEK1_UP | 162 | 0.459 | 1.958 | 0 | 0.036 | 0.256 | 3509 | tags=35%, list=20%, signal=43% |
| IRITANI_ADPROX_DN | 56 | 0.534 | 1.945 | 0.002 | 0.037 | 0.294 | 3314 | tags=48%, list=19%, signal=59% |
| IGF_VS_PDGF_DN | 41 | 0.565 | 1.922 | 0 | 0.041 | 0.402 | 1366 | tags=27%, list=8%, signal=29% |
| UVB_NHEK1_C2 | 22 | 0.656 | 1.917 | 0 | 0.041 | 0.428 | 3509 | tags=59%, list=20%, signal=74% |
| DAC_PANC50_UP | 40 | 0.575 | 1.912 | 0.002 | 0.041 | 0.455 | 2324 | tags=35%, list=13%, signal=40% |
| HINATA_NFKB_UP | 105 | 0.466 | 1.882 | 0 | 0.048 | 0.597 | 3024 | tags=35%, list=17%, signal=42% |
| DAC_BLADDER_UP | 26 | 0.613 | 1.891 | 0.002 | 0.049 | 0.564 | 1147 | tags=35%, list=7%, signal=37% |
| HBX_HCC_UP | 16 | 0.678 | 1.894 | 0.002 | 0.049 | 0.545 | 1769 | tags=44%, list=10%, signal=49% |
| TAKEDA_NUP8_HOXA9_16D_UP | 143 | 0.442 | 1.867 | 0 | 0.051 | 0.668 | 2770 | tags=31%, list=16%, signal=37% |
| HSA04950_MATURITY_ONSET_DIABETES_OF_THE_YOUNG | 20 | 0.633 | 1.883 | 0 | 0.051 | 0.596 | 3814 | tags=60%, list=22%, signal=77% |
| TAKEDA_NUP8_HOXA9_8D_UP | 135 | 0.445 | 1.868 | 0 | 0.052 | 0.665 | 2448 | tags=31%, list=14%, signal=36% |
| STEMPATHWAY | 15 | 0.691 | 1.869 | 0 | 0.054 | 0.661 | 966 | tags=53%, list=6%, signal=56% |
| BRG1_SW13_UP | 42 | 0.548 | 1.854 | 0 | 0.056 | 0.724 | 4893 | tags=55%, list=28%, signal=76% |
| HSA04740_OLFACTORY_TRANSDUCTION | 30 | 0.572 | 1.83 | 0.002 | 0.07 | 0.823 | 973 | tags=23%, list=6%, signal=25% |
| GALINDO_ACT_UP | 76 | 0.478 | 1.833 | 0 | 0.071 | 0.813 | 3521 | tags=33%, list=20%, signal=41% |
| TAKEDA_NUP8_HOXA9_6H_UP | 76 | 0.48 | 1.82 | 0 | 0.076 | 0.867 | 2732 | tags=36%, list=16%, signal=42% |
| VERHAAK_AML_NPM1_MUT_VS_WT_UP | 158 | 0.429 | 1.808 | 0 | 0.078 | 0.899 | 3328 | tags=36%, list=19%, signal=44% |
| HSA04640_HEMATOPOIETIC_CELL_LINEAGE | 83 | 0.465 | 1.813 | 0 | 0.079 | 0.883 | 2277 | tags=37%, list=13%, signal=43% |
| TAKEDA_NUP8_HOXA9_10D_DN | 139 | 0.43 | 1.801 | 0 | 0.08 | 0.922 | 1938 | tags=30%, list=11%, signal=34% |
| RUTELLA_HEPATGFSNDCS_UP | 142 | 0.432 | 1.802 | 0 | 0.081 | 0.92 | 3495 | tags=36%, list=20%, signal=45% |
| YANG_OSTECLASTS_SIG | 39 | 0.526 | 1.808 | 0 | 0.081 | 0.898 | 1529 | tags=38%, list=9%, signal=42% |
| AGEING_KIDNEY_UP | 362 | 0.381 | 1.795 | 0 | 0.082 | 0.932 | 3045 | tags=29%, list=17%, signal=34% |
| DER_IFNA_UP | 62 | 0.481 | 1.79 | 0 | 0.082 | 0.945 | 3449 | tags=35%, list=20%, signal=44% |
| ADIPOGENESIS_HMSC_CLASS1_UP | 16 | 0.649 | 1.792 | 0.004 | 0.082 | 0.937 | 3297 | tags=50%, list=19%, signal=62% |
| AGEING_KIDNEY_SPECIFIC_UP | 165 | 0.407 | 1.764 | 0 | 0.085 | 0.975 | 3039 | tags=32%, list=17%, signal=38% |
| IL1_CORNEA_UP | 61 | 0.49 | 1.785 | 0 | 0.085 | 0.955 | 3226 | tags=38%, list=18%, signal=46% |
| ICHIBA_GVHD | 230 | 0.395 | 1.782 | 0 | 0.086 | 0.959 | 3253 | tags=30%, list=19%, signal=37% |
| IMMUNE_RESPONSE | 211 | 0.396 | 1.765 | 0 | 0.087 | 0.972 | 2854 | tags=31%, list=16%, signal=37% |
| CMV_8HRS_UP | 29 | 0.567 | 1.766 | 0.002 | 0.088 | 0.972 | 860 | tags=21%, list=5%, signal=22% |
| DEFENSE_RESPONSE | 242 | 0.385 | 1.751 | 0 | 0.09 | 0.982 | 2489 | tags=28%, list=14%, signal=32% |
| HSA04060_CYTOKINE_CYTOKINE_RECEPTOR_INTERACTION | 235 | 0.398 | 1.769 | 0 | 0.09 | 0.97 | 2260 | tags=29%, list=13%, signal=32% |
| IRITANI_ADPROX_VASC | 146 | 0.418 | 1.766 | 0 | 0.09 | 0.972 | 3449 | tags=36%, list=20%, signal=45% |
| NAKAJIMA_MCS_UP | 86 | 0.445 | 1.753 | 0 | 0.09 | 0.98 | 2263 | tags=34%, list=13%, signal=39% |
| ADIP_DIFF_CLUSTER2 | 39 | 0.53 | 1.755 | 0.004 | 0.09 | 0.979 | 2052 | tags=26%, list=12%, signal=29% |
| VIRAL_GENOME_REPLICATION | 19 | 0.614 | 1.774 | 0.008 | 0.09 | 0.967 | 3694 | tags=53%, list=21%, signal=67% |
| HALMOS_CEBP_UP | 47 | 0.505 | 1.756 | 0.002 | 0.091 | 0.979 | 2742 | tags=34%, list=16%, signal=40% |
| JNK_UP | 28 | 0.557 | 1.771 | 0.007 | 0.091 | 0.968 | 810 | tags=21%, list=5%, signal=22% |
| IFN_ALPHA_UP | 38 | 0.524 | 1.746 | 0.004 | 0.092 | 0.986 | 3449 | tags=39%, list=20%, signal=49% |
| SCHURINGA_STAT5A_DN | 17 | 0.632 | 1.746 | 0.01 | 0.094 | 0.986 | 2365 | tags=47%, list=14%, signal=54% |
| NF90_UP | 23 | 0.589 | 1.739 | 0.002 | 0.096 | 0.99 | 1147 | tags=30%, list=7%, signal=33% |
| IFNA_HCMV_6HRS_UP | 48 | 0.498 | 1.737 | 0.002 | 0.097 | 0.991 | 3217 | tags=33%, list=18%, signal=41% |
| YAO_P4_KO_VS_WT_UP | 66 | 0.462 | 1.727 | 0 | 0.101 | 0.993 | 1669 | tags=23%, list=10%, signal=25% |
| VIRAL_INFECTIOUS_CYCLE | 28 | 0.552 | 1.731 | 0.004 | 0.101 | 0.993 | 3194 | tags=43%, list=18%, signal=52% |
| CHEMOKINE_RECEPTOR_BINDING | 42 | 0.501 | 1.728 | 0.002 | 0.102 | 0.993 | 1600 | tags=31%, list=9%, signal=34% |
| BOQUEST_CD31PLUS_VS_CD31MINUS_DN | 245 | 0.379 | 1.722 | 0 | 0.105 | 0.995 | 2400 | tags=27%, list=14%, signal=30% |
| INFLAMMATORY_RESPONSE | 120 | 0.418 | 1.712 | 0.002 | 0.11 | 1 | 2489 | tags=33%, list=14%, signal=38% |
| CMV_HCMV_TIMECOURSE_20HRS_DN | 37 | 0.507 | 1.713 | 0.005 | 0.11 | 1 | 2260 | tags=35%, list=13%, signal=40% |
| CELLULAR_DEFENSE_RESPONSE | 50 | 0.483 | 1.715 | 0.002 | 0.112 | 1 | 2140 | tags=36%, list=12%, signal=41% |
| ADIPOGENESIS_HMSC_CLASS8_DN | 30 | 0.533 | 1.713 | 0.009 | 0.112 | 1 | 1830 | tags=30%, list=10%, signal=33% |
| WIELAND_HEPATITIS_B_INDUCED | 91 | 0.431 | 1.705 | 0 | 0.113 | 1 | 3349 | tags=36%, list=19%, signal=45% |
| TARTE_MATURE_PC | 351 | 0.359 | 1.697 | 0 | 0.119 | 1 | 3066 | tags=28%, list=18%, signal=33% |
| SANA_IFNG_ENDOTHELIAL_UP | 73 | 0.442 | 1.697 | 0.004 | 0.12 | 1 | 3253 | tags=37%, list=19%, signal=45% |
| ERM_KO_SERTOLI_DN | 16 | 0.619 | 1.693 | 0.013 | 0.12 | 1 | 1352 | tags=38%, list=8%, signal=41% |
| GLUTATHIONE_TRANSFERASE_ACTIVITY | 15 | 0.626 | 1.688 | 0.009 | 0.121 | 1 | 3087 | tags=47%, list=18%, signal=57% |
| CROONQUIST_RAS_STROMA_UP | 24 | 0.562 | 1.693 | 0.012 | 0.121 | 1 | 1346 | tags=29%, list=8%, signal=32% |
| STAEGE_EFTS_UP | 26 | 0.554 | 1.69 | 0.013 | 0.121 | 1 | 3101 | tags=50%, list=18%, signal=61% |
| METALLOENDOPEPTIDASE_ACTIVITY | 26 | 0.54 | 1.683 | 0.015 | 0.122 | 1 | 1814 | tags=31%, list=10%, signal=34% |
| OXIDOREDUCTASE_ACTIVITY__ACTING_ON_CH_OH_GROUP_OF_DONORS | 54 | 0.468 | 1.683 | 0.002 | 0.124 | 1 | 3739 | tags=41%, list=21%, signal=52% |
| KNUDSEN_PMNS_UP | 73 | 0.438 | 1.683 | 0.002 | 0.125 | 1 | 2852 | tags=30%, list=16%, signal=36% |
| ZUCCHI_EPITHELIAL_DN | 45 | 0.487 | 1.676 | 0.003 | 0.127 | 1 | 1148 | tags=22%, list=7%, signal=24% |
| VIRAL_REPRODUCTION | 36 | 0.506 | 1.675 | 0.005 | 0.127 | 1 | 3194 | tags=36%, list=18%, signal=44% |
| HSA00361_GAMMA_HEXACHLOROCYCLOHEXANE_DEGRADATION | 22 | 0.567 | 1.677 | 0.004 | 0.128 | 1 | 1128 | tags=27%, list=6%, signal=29% |
| TSA_PANC50_UP | 38 | 0.499 | 1.672 | 0.005 | 0.128 | 1 | 2424 | tags=37%, list=14%, signal=43% |
| VIRAL_REPRODUCTIVE_PROCESS | 32 | 0.514 | 1.665 | 0.011 | 0.131 | 1 | 3194 | tags=38%, list=18%, signal=46% |
| GERY_CEBP_TARGETS | 106 | 0.407 | 1.666 | 0.002 | 0.132 | 1 | 2686 | tags=31%, list=15%, signal=37% |
| ET743_HELA_UP | 53 | 0.464 | 1.667 | 0.009 | 0.132 | 1 | 4173 | tags=40%, list=24%, signal=52% |
| AGED_MOUSE_MUSCLE_UP | 29 | 0.523 | 1.662 | 0.016 | 0.132 | 1 | 3471 | tags=48%, list=20%, signal=60% |
| ELECTRON_TRANSPORT_CHAIN | 98 | 0.412 | 1.641 | 0.007 | 0.134 | 1 | 5876 | tags=62%, list=34%, signal=93% |
| HSA04620_TOLL_LIKE_RECEPTOR_SIGNALING_PATHWAY | 92 | 0.414 | 1.639 | 0.002 | 0.135 | 1 | 1985 | tags=23%, list=11%, signal=26% |
| TAKEDA_NUP8_HOXA9_8D_DN | 187 | 0.373 | 1.636 | 0 | 0.136 | 1 | 3165 | tags=33%, list=18%, signal=40% |
| AUXILIARY_TRANSPORT_PROTEIN_ACTIVITY | 24 | 0.544 | 1.641 | 0.024 | 0.136 | 1 | 1300 | tags=25%, list=7%, signal=27% |
| INTERMEDIATE_FILAMENT | 19 | 0.574 | 1.635 | 0.027 | 0.136 | 1 | 2032 | tags=37%, list=12%, signal=42% |
| BAF57_BT549_UP | 214 | 0.371 | 1.646 | 0 | 0.137 | 1 | 3225 | tags=30%, list=18%, signal=36% |
| LEE_CIP_DN | 63 | 0.433 | 1.633 | 0.005 | 0.137 | 1 | 2038 | tags=29%, list=12%, signal=32% |
| TAVOR_CEBP_UP | 48 | 0.469 | 1.641 | 0.006 | 0.137 | 1 | 3280 | tags=40%, list=19%, signal=49% |
| LAL_KO_6MO_UP | 62 | 0.443 | 1.648 | 0.007 | 0.137 | 1 | 1831 | tags=29%, list=10%, signal=32% |
| G_PROTEIN_COUPLED_RECEPTOR_BINDING | 51 | 0.465 | 1.637 | 0.011 | 0.137 | 1 | 1784 | tags=29%, list=10%, signal=33% |
| INTERMEDIATE_FILAMENT_CYTOSKELETON | 19 | 0.574 | 1.646 | 0.011 | 0.137 | 1 | 2032 | tags=37%, list=12%, signal=42% |
| PASSERINI_INFLAMMATION | 24 | 0.542 | 1.643 | 0.012 | 0.137 | 1 | 2852 | tags=46%, list=16%, signal=55% |
| LVAD_HEARTFAILURE_DN | 36 | 0.491 | 1.631 | 0.013 | 0.137 | 1 | 3107 | tags=50%, list=18%, signal=61% |
| CMV-UV_HCMV_6HRS_UP | 114 | 0.393 | 1.644 | 0 | 0.138 | 1 | 2393 | tags=23%, list=14%, signal=26% |
| SHEPARD_NEG_REG_OF_CELL_PROLIFERATION | 110 | 0.403 | 1.649 | 0 | 0.138 | 1 | 3319 | tags=32%, list=19%, signal=39% |
| CALCIUM_INDEPENDENT_CELL_CELL_ADHESION | 19 | 0.565 | 1.656 | 0.009 | 0.138 | 1 | 1323 | tags=26%, list=8%, signal=28% |
| WANG_HOXA9_VS_MEIS1_DN | 21 | 0.565 | 1.652 | 0.017 | 0.138 | 1 | 3439 | tags=62%, list=20%, signal=77% |
| LAL_KO_3MO_UP | 47 | 0.475 | 1.652 | 0.009 | 0.139 | 1 | 1529 | tags=30%, list=9%, signal=33% |
| IFN_BETA_GLIOMA_DN | 43 | 0.48 | 1.649 | 0.015 | 0.139 | 1 | 3243 | tags=33%, list=19%, signal=40% |
| LEE_TCELLS7_UP | 15 | 0.631 | 1.654 | 0.016 | 0.139 | 1 | 3608 | tags=67%, list=21%, signal=84% |
| IFNA_UV-CMV_COMMON_HCMV_6HRS_UP | 26 | 0.52 | 1.629 | 0.025 | 0.139 | 1 | 4241 | tags=50%, list=24%, signal=66% |
| CHIARETTI_T_ALL_DIFF | 248 | 0.355 | 1.623 | 0 | 0.143 | 1 | 3500 | tags=33%, list=20%, signal=40% |
| RESPONSE_TO_WOUNDING | 177 | 0.368 | 1.624 | 0 | 0.143 | 1 | 2489 | tags=28%, list=14%, signal=32% |
| BENNETT_SLE_UP | 26 | 0.531 | 1.62 | 0.018 | 0.144 | 1 | 2428 | tags=35%, list=14%, signal=40% |
| TSADAC_PANC50_UP | 40 | 0.475 | 1.616 | 0.007 | 0.145 | 1 | 2909 | tags=35%, list=17%, signal=42% |
| OXIDOREDUCTASE_ACTIVITY_GO_0016616 | 50 | 0.458 | 1.62 | 0.009 | 0.145 | 1 | 3739 | tags=40%, list=21%, signal=51% |
| CHEMOKINE_ACTIVITY | 41 | 0.479 | 1.616 | 0.015 | 0.146 | 1 | 1600 | tags=29%, list=9%, signal=32% |
| HOGERKORP_ANTI_CD44_UP | 24 | 0.526 | 1.616 | 0.006 | 0.147 | 1 | 2398 | tags=29%, list=14%, signal=34% |
| MULTI_ORGANISM_PROCESS | 134 | 0.387 | 1.61 | 0.002 | 0.151 | 1 | 2143 | tags=25%, list=12%, signal=28% |
| FLECHNER_KIDNEY_TRANSPLANT_REJECTION_UP | 76 | 0.424 | 1.609 | 0.002 | 0.151 | 1 | 2365 | tags=26%, list=14%, signal=30% |
| ADIP_VS_PREADIP_DN | 38 | 0.47 | 1.609 | 0.015 | 0.151 | 1 | 2517 | tags=26%, list=14%, signal=31% |
| DAC_IFN_BLADDER_UP | 17 | 0.578 | 1.603 | 0.021 | 0.156 | 1 | 3011 | tags=47%, list=17%, signal=57% |
| FEMALE_GAMETE_GENERATION | 16 | 0.596 | 1.602 | 0.028 | 0.156 | 1 | 2000 | tags=31%, list=11%, signal=35% |
| ET743_SARCOMA_72HRS_UP | 58 | 0.442 | 1.597 | 0.008 | 0.16 | 1 | 3403 | tags=33%, list=19%, signal=41% |
| CMV_UV-CMV_COMMON_HCMV_6HRS_UP | 19 | 0.557 | 1.598 | 0.014 | 0.16 | 1 | 4276 | tags=53%, list=24%, signal=70% |
| APOPTOSIS_KEGG | 48 | 0.457 | 1.595 | 0.009 | 0.161 | 1 | 1637 | tags=19%, list=9%, signal=21% |
| IFN_GAMMA_UP | 37 | 0.472 | 1.594 | 0.011 | 0.161 | 1 | 3449 | tags=32%, list=20%, signal=40% |
| ECTODERM_DEVELOPMENT | 72 | 0.415 | 1.587 | 0.007 | 0.162 | 1 | 3237 | tags=29%, list=18%, signal=36% |
| KERATINOCYTE_DIFFERENTIATION | 15 | 0.59 | 1.591 | 0.029 | 0.162 | 1 | 5552 | tags=67%, list=32%, signal=98% |
| CELL_CELL_SIGNALING | 380 | 0.332 | 1.59 | 0 | 0.163 | 1 | 3338 | tags=33%, list=19%, signal=39% |
| KANG_TERT_UP | 79 | 0.409 | 1.588 | 0.004 | 0.163 | 1 | 3015 | tags=30%, list=17%, signal=37% |
| RESPONSE_TO_VIRUS | 41 | 0.458 | 1.592 | 0.013 | 0.163 | 1 | 4206 | tags=51%, list=24%, signal=67% |
| TRANSFERASE_ACTIVITY__TRANSFERRING_ALKYL_OR_ARYL__OTHER_THAN_METHYL__GROUPS | 26 | 0.515 | 1.589 | 0.022 | 0.163 | 1 | 3252 | tags=31%, list=19%, signal=38% |
| SHIPP_DLBCL_CURED_UP | 29 | 0.493 | 1.579 | 0.016 | 0.171 | 1 | 3608 | tags=45%, list=21%, signal=56% |
| RADAEVA_IFNA_UP | 48 | 0.443 | 1.58 | 0.017 | 0.171 | 1 | 2428 | tags=27%, list=14%, signal=31% |
| REPRODUCTION | 226 | 0.346 | 1.568 | 0 | 0.178 | 1 | 3984 | tags=37%, list=23%, signal=47% |
| JECHLINGER_EMT_UP | 57 | 0.429 | 1.568 | 0.008 | 0.179 | 1 | 2453 | tags=32%, list=14%, signal=37% |
| HSA00190_OXIDATIVE_PHOSPHORYLATION | 107 | 0.384 | 1.57 | 0.012 | 0.179 | 1 | 3148 | tags=34%, list=18%, signal=41% |
| CROONQUIST_IL6_STROMA_UP | 37 | 0.468 | 1.566 | 0.021 | 0.179 | 1 | 2561 | tags=32%, list=15%, signal=38% |
| INFLAMPATHWAY | 27 | 0.507 | 1.573 | 0.022 | 0.179 | 1 | 2633 | tags=44%, list=15%, signal=52% |
| POSITIVE_REGULATION_OF_CELL_DIFFERENTIATION | 21 | 0.535 | 1.571 | 0.039 | 0.179 | 1 | 4146 | tags=52%, list=24%, signal=69% |
| EXTRACELLULAR_MATRIX_PART | 52 | 0.438 | 1.568 | 0.012 | 0.18 | 1 | 1010 | tags=19%, list=6%, signal=20% |
| BLEO_HUMAN_LYMPH_HIGH_24HRS_UP | 92 | 0.394 | 1.563 | 0.003 | 0.182 | 1 | 3139 | tags=33%, list=18%, signal=40% |
| MATRIX_METALLOPROTEINASES | 29 | 0.495 | 1.56 | 0.021 | 0.186 | 1 | 2436 | tags=41%, list=14%, signal=48% |
| PROLIFERATION_GENES | 368 | 0.332 | 1.556 | 0 | 0.189 | 1 | 3241 | tags=28%, list=19%, signal=33% |
| LEE_E2F1_UP | 62 | 0.426 | 1.557 | 0.014 | 0.189 | 1 | 2957 | tags=31%, list=17%, signal=37% |
| HBX_NL_DN | 16 | 0.564 | 1.552 | 0.036 | 0.193 | 1 | 2488 | tags=44%, list=14%, signal=51% |
| TIGHT_JUNCTION | 26 | 0.504 | 1.545 | 0.032 | 0.203 | 1 | 3675 | tags=35%, list=21%, signal=44% |
| VOLTAGE_GATED_CALCIUM_CHANNEL_COMPLEX | 15 | 0.575 | 1.543 | 0.039 | 0.203 | 1 | 3095 | tags=53%, list=18%, signal=65% |
| CELL_SURFACE_RECEPTOR_LINKED_SIGNAL_TRANSDUCTION | 134 | 0.365 | 1.541 | 0.003 | 0.204 | 1 | 2852 | tags=28%, list=16%, signal=33% |
| LYMPHOCYTE_DIFFERENTIATION | 25 | 0.514 | 1.544 | 0.026 | 0.204 | 1 | 2617 | tags=40%, list=15%, signal=47% |
| DAC_PANC_UP | 351 | 0.327 | 1.536 | 0 | 0.205 | 1 | 2550 | tags=25%, list=15%, signal=28% |
| EPIDERMIS_DEVELOPMENT | 64 | 0.415 | 1.538 | 0.011 | 0.205 | 1 | 3237 | tags=31%, list=18%, signal=38% |
| HDACI_COLON_BUT24HRS_UP | 59 | 0.418 | 1.537 | 0.014 | 0.205 | 1 | 2692 | tags=31%, list=15%, signal=36% |
| TOB1PATHWAY | 18 | 0.55 | 1.541 | 0.046 | 0.205 | 1 | 2110 | tags=39%, list=12%, signal=44% |
| MAMMARY_DEV_UP | 53 | 0.434 | 1.538 | 0.022 | 0.206 | 1 | 3688 | tags=38%, list=21%, signal=48% |
| REPRODUCTIVE_PROCESS | 138 | 0.361 | 1.529 | 0 | 0.211 | 1 | 3890 | tags=38%, list=22%, signal=49% |
| DORSEY_DOXYCYCLINE_UP | 30 | 0.491 | 1.53 | 0.03 | 0.211 | 1 | 2343 | tags=33%, list=13%, signal=38% |
| BASSO_GERMINAL_CENTER_CD40_DN | 61 | 0.411 | 1.53 | 0.011 | 0.212 | 1 | 2423 | tags=33%, list=14%, signal=38% |
| ZHAN_MMPC_SIMAL | 45 | 0.431 | 1.527 | 0.02 | 0.212 | 1 | 4799 | tags=51%, list=27%, signal=70% |
| LIPID_RAFT | 25 | 0.493 | 1.527 | 0.036 | 0.212 | 1 | 3333 | tags=44%, list=19%, signal=54% |
| CYTOKINEPATHWAY | 20 | 0.529 | 1.526 | 0.044 | 0.212 | 1 | 756 | tags=30%, list=4%, signal=31% |
| HSA04340_HEDGEHOG_SIGNALING_PATHWAY | 54 | 0.423 | 1.531 | 0.01 | 0.213 | 1 | 3149 | tags=26%, list=18%, signal=32% |
| LU_IL4BCELL | 66 | 0.41 | 1.524 | 0.022 | 0.213 | 1 | 3602 | tags=38%, list=21%, signal=48% |
| KANG_TERT_DN | 92 | 0.377 | 1.521 | 0.002 | 0.214 | 1 | 3412 | tags=35%, list=19%, signal=43% |
| ROS_MOUSE_AORTA_DN | 73 | 0.4 | 1.522 | 0.016 | 0.215 | 1 | 2453 | tags=25%, list=14%, signal=29% |
| BIOGENIC_AMINE_SYNTHESIS | 16 | 0.556 | 1.521 | 0.04 | 0.215 | 1 | 3294 | tags=44%, list=19%, signal=54% |
| RESPONSE_TO_OTHER_ORGANISM | 70 | 0.398 | 1.521 | 0.022 | 0.216 | 1 | 4293 | tags=41%, list=25%, signal=55% |
| CHIARETTI_T_ALL | 226 | 0.335 | 1.512 | 0.003 | 0.225 | 1 | 3500 | tags=32%, list=20%, signal=39% |
| ION_HOMEOSTASIS | 116 | 0.362 | 1.51 | 0.009 | 0.225 | 1 | 2418 | tags=28%, list=14%, signal=33% |
| CHANNEL_REGULATOR_ACTIVITY | 22 | 0.52 | 1.513 | 0.034 | 0.225 | 1 | 1300 | tags=23%, list=7%, signal=25% |
| EICOSANOID_SYNTHESIS | 18 | 0.554 | 1.51 | 0.045 | 0.226 | 1 | 1632 | tags=39%, list=9%, signal=43% |
| EXTRACELLULAR_REGION | 413 | 0.313 | 1.507 | 0 | 0.228 | 1 | 2596 | tags=26%, list=15%, signal=29% |
| ET743_SARCOMA_6HRS_UP | 28 | 0.476 | 1.498 | 0.048 | 0.228 | 1 | 1533 | tags=21%, list=9%, signal=23% |
| HOUSTIS_ROS | 31 | 0.472 | 1.506 | 0.05 | 0.228 | 1 | 3521 | tags=35%, list=20%, signal=44% |
| IMMUNE_SYSTEM_PROCESS | 300 | 0.324 | 1.5 | 0 | 0.229 | 1 | 2854 | tags=26%, list=16%, signal=31% |
| UV-CMV_UNIQUE_HCMV_6HRS_UP | 96 | 0.378 | 1.507 | 0.011 | 0.229 | 1 | 2393 | tags=22%, list=14%, signal=25% |
| HPV31_UP | 52 | 0.418 | 1.498 | 0.026 | 0.229 | 1 | 701 | tags=12%, list=4%, signal=12% |
| LEUKOCYTE_DIFFERENTIATION | 36 | 0.454 | 1.505 | 0.041 | 0.229 | 1 | 2617 | tags=36%, list=15%, signal=42% |
| FERRARI_4HPR_UP | 22 | 0.505 | 1.499 | 0.031 | 0.23 | 1 | 4400 | tags=55%, list=25%, signal=73% |
| RAS_ONCOGENIC_SIGNATURE | 235 | 0.331 | 1.5 | 0.002 | 0.233 | 1 | 2424 | tags=22%, list=14%, signal=25% |
| DER_IFNG_UP | 59 | 0.402 | 1.489 | 0.03 | 0.236 | 1 | 3449 | tags=31%, list=20%, signal=38% |
| BRENTANI_IMMUNE_FUNCTION | 50 | 0.422 | 1.489 | 0.031 | 0.236 | 1 | 2936 | tags=32%, list=17%, signal=38% |
| LEE_TCELLS6_UP | 22 | 0.506 | 1.488 | 0.044 | 0.236 | 1 | 1967 | tags=27%, list=11%, signal=31% |
| ROSS_MLL_FUSION | 70 | 0.392 | 1.49 | 0.027 | 0.237 | 1 | 4640 | tags=37%, list=26%, signal=50% |
| RECEPTOR_COMPLEX | 51 | 0.423 | 1.49 | 0.03 | 0.238 | 1 | 2534 | tags=31%, list=14%, signal=37% |
| TAKEDA_NUP8_HOXA9_16D_DN | 194 | 0.338 | 1.49 | 0 | 0.239 | 1 | 2853 | tags=27%, list=16%, signal=32% |
| IL6_FIBRO_UP | 43 | 0.432 | 1.491 | 0.043 | 0.239 | 1 | 3081 | tags=37%, list=18%, signal=45% |
| EGF_HDMEC_UP | 41 | 0.44 | 1.485 | 0.031 | 0.24 | 1 | 4691 | tags=49%, list=27%, signal=66% |
| IMMUNE_EFFECTOR_PROCESS | 32 | 0.462 | 1.484 | 0.034 | 0.241 | 1 | 3918 | tags=47%, list=22%, signal=60% |

| **Gene Set (Down-Regulated)** | **Size** | **ES** | **NES** | **NOM p-val** | **FDR q-val** | **FWER p-val** | **Rank at Max** | **Leading Edge** |
| --- | --- | --- | --- | --- | --- | --- | --- | --- |
| ZHAN_MM_CD138_PR_VS_REST | 36 | -0.643 | -2.253 | 0 | 0.002 | 0.002 | 4544 | tags=69%, list=26%, signal=94% |
| SERUM_FIBROBLAST_CELLCYCLE | 115 | -0.502 | -2.182 | 0 | 0.003 | 0.007 | 4541 | tags=55%, list=26%, signal=73% |
| CELL_CYCLE_PROCESS | 162 | -0.472 | -2.164 | 0 | 0.003 | 0.01 | 4726 | tags=48%, list=27%, signal=65% |
| M_PHASE_OF_MITOTIC_CELL_CYCLE | 68 | -0.521 | -2.068 | 0 | 0.014 | 0.065 | 2441 | tags=37%, list=14%, signal=43% |
| MITOSIS | 65 | -0.524 | -2.071 | 0 | 0.016 | 0.063 | 2441 | tags=37%, list=14%, signal=43% |
| CROONQUIST_IL6_STARVE_UP | 32 | -0.59 | -1.988 | 0.002 | 0.027 | 0.199 | 4085 | tags=66%, list=23%, signal=85% |
| M_PHASE | 95 | -0.482 | -2.007 | 0 | 0.028 | 0.156 | 2441 | tags=36%, list=14%, signal=41% |
| CELL_CYCLE_PHASE | 142 | -0.454 | -1.997 | 0 | 0.028 | 0.181 | 4726 | tags=48%, list=27%, signal=65% |
| P21_P53_ANY_DN | 42 | -0.553 | -1.961 | 0 | 0.032 | 0.281 | 5100 | tags=67%, list=29%, signal=94% |
| MITOTIC_CELL_CYCLE | 125 | -0.447 | -1.966 | 0 | 0.033 | 0.264 | 4726 | tags=45%, list=27%, signal=61% |
| LEE_TCELLS3_UP | 90 | -0.47 | -1.949 | 0 | 0.035 | 0.322 | 4631 | tags=54%, list=26%, signal=74% |
| IDX_TSA_UP_CLUSTER3 | 82 | -0.473 | -1.943 | 0 | 0.036 | 0.35 | 6237 | tags=63%, list=36%, signal=98% |
| BRENTANI_REPAIR | 37 | -0.549 | -1.896 | 0 | 0.056 | 0.514 | 5418 | tags=57%, list=31%, signal=82% |
| MICROTUBULE_CYTOSKELETON | 125 | -0.429 | -1.864 | 0 | 0.078 | 0.669 | 3598 | tags=38%, list=21%, signal=47% |
| CELL_CYCLE_CHECKPOINT_GO_0000075 | 40 | -0.536 | -1.853 | 0 | 0.082 | 0.709 | 5211 | tags=68%, list=30%, signal=96% |
| GOLDRATH_CELLCYCLE | 26 | -0.572 | -1.845 | 0.002 | 0.083 | 0.731 | 4085 | tags=50%, list=23%, signal=65% |
| MICROTUBULE_ORGANIZING_CENTER | 57 | -0.477 | -1.834 | 0.004 | 0.084 | 0.772 | 4311 | tags=49%, list=25%, signal=65% |
| DNA_REPAIR | 107 | -0.424 | -1.827 | 0 | 0.087 | 0.799 | 6101 | tags=54%, list=35%, signal=83% |
| CHOLESTEROL_BIOSYNTHESIS | 15 | -0.683 | -1.834 | 0.006 | 0.089 | 0.772 | 5131 | tags=80%, list=29%, signal=113% |
| DOX_RESIST_GASTRIC_UP | 39 | -0.513 | -1.773 | 0.006 | 0.124 | 0.946 | 4996 | tags=56%, list=29%, signal=79% |
| CROONQUIST_IL6_RAS_DN | 22 | -0.571 | -1.785 | 0.015 | 0.126 | 0.925 | 4491 | tags=64%, list=26%, signal=85% |
| SPINDLE | 35 | -0.528 | -1.775 | 0 | 0.127 | 0.943 | 3598 | tags=51%, list=21%, signal=65% |
| CHROMOSOME__PERICENTRIC_REGION | 26 | -0.547 | -1.776 | 0.002 | 0.131 | 0.942 | 4850 | tags=54%, list=28%, signal=74% |
| RNA_HELICASE_ACTIVITY | 21 | -0.591 | -1.76 | 0.002 | 0.132 | 0.959 | 5199 | tags=71%, list=30%, signal=101% |
| PROTEIN_DNA_COMPLEX_ASSEMBLY | 35 | -0.502 | -1.746 | 0.002 | 0.134 | 0.969 | 4207 | tags=46%, list=24%, signal=60% |
| HELICASE_ACTIVITY | 42 | -0.488 | -1.761 | 0 | 0.137 | 0.959 | 5719 | tags=60%, list=33%, signal=88% |
| P21_P53_MIDDLE_DN | 21 | -0.586 | -1.747 | 0.007 | 0.138 | 0.969 | 5100 | tags=67%, list=29%, signal=94% |
| IDX_TSA_DN_CLUSTER6 | 22 | -0.567 | -1.75 | 0.006 | 0.14 | 0.967 | 4211 | tags=50%, list=24%, signal=66% |
| DNA_HELICASE_ACTIVITY | 18 | -0.599 | -1.736 | 0.008 | 0.143 | 0.977 | 5719 | tags=67%, list=33%, signal=99% |
| REGULATION_OF_SMALL_GTPASE_MEDIATED_SIGNAL_TRANSDUCTION | 22 | -0.555 | -1.713 | 0.002 | 0.162 | 0.996 | 2407 | tags=27%, list=14%, signal=32% |
| REGULATION_OF_MITOSIS | 35 | -0.51 | -1.715 | 0.004 | 0.164 | 0.995 | 5004 | tags=54%, list=29%, signal=76% |
| NUCLEOTIDE_METABOLISM | 16 | -0.609 | -1.717 | 0.006 | 0.167 | 0.993 | 3352 | tags=56%, list=19%, signal=69% |
| CELL_CYCLE_GO_0007049 | 274 | -0.345 | -1.705 | 0 | 0.168 | 0.997 | 5004 | tags=42%, list=29%, signal=58% |
| CENTROSOME | 49 | -0.465 | -1.686 | 0.002 | 0.185 | 0.999 | 4311 | tags=49%, list=25%, signal=65% |
| CHROMOSOME_SEGREGATION | 22 | -0.556 | -1.687 | 0.002 | 0.188 | 0.999 | 4850 | tags=55%, list=28%, signal=75% |
| VERNELL_PRB_CLSTR1 | 58 | -0.44 | -1.688 | 0.002 | 0.192 | 0.999 | 6063 | tags=57%, list=35%, signal=87% |
| MITOTIC_CELL_CYCLE_CHECKPOINT | 18 | -0.575 | -1.678 | 0.014 | 0.193 | 0.999 | 2441 | tags=44%, list=14%, signal=52% |
| HOFFMANN_BIVSBII_BI_TABLE2 | 181 | -0.368 | -1.663 | 0 | 0.215 | 1 | 3804 | tags=36%, list=22%, signal=45% |
| SPINDLE_POLE | 17 | -0.579 | -1.659 | 0.009 | 0.217 | 1 | 2711 | tags=53%, list=15%, signal=63% |
| INTERPHASE | 57 | -0.443 | -1.653 | 0.007 | 0.224 | 1 | 4726 | tags=44%, list=27%, signal=60% |
| INTERPHASE_OF_MITOTIC_CELL_CYCLE | 54 | -0.435 | -1.638 | 0.002 | 0.231 | 1 | 4726 | tags=43%, list=27%, signal=58% |
| HDACI_COLON_CLUSTER10 | 38 | -0.476 | -1.64 | 0.011 | 0.232 | 1 | 5325 | tags=47%, list=30%, signal=68% |
| KINETOCHORE | 21 | -0.549 | -1.63 | 0.015 | 0.236 | 1 | 2496 | tags=33%, list=14%, signal=39% |
| YU_CMYC_UP | 29 | -0.492 | -1.641 | 0.015 | 0.236 | 1 | 5093 | tags=52%, list=29%, signal=73% |
| SIG_PIP3_SIGNALING_IN_CARDIAC_MYOCTES | 66 | -0.412 | -1.63 | 0.005 | 0.241 | 1 | 5234 | tags=45%, list=30%, signal=65% |
| DAMAGED_DNA_BINDING | 17 | -0.562 | -1.642 | 0.022 | 0.241 | 1 | 5211 | tags=65%, list=30%, signal=92% |
| MANALO_HYPOXIA_DN | 82 | -0.391 | -1.618 | 0.007 | 0.242 | 1 | 5486 | tags=46%, list=31%, signal=67% |
| MICROTUBULE_BINDING | 27 | -0.497 | -1.611 | 0.015 | 0.242 | 1 | 3247 | tags=41%, list=19%, signal=50% |
| DNA_REPLICATION_REACTOME | 46 | -0.443 | -1.613 | 0.011 | 0.243 | 1 | 6652 | tags=59%, list=38%, signal=94% |
| CPR_LOW_LIVER_UP | 16 | -0.576 | -1.614 | 0.018 | 0.244 | 1 | 4873 | tags=56%, list=28%, signal=78% |
| CELL_CYCLE_KEGG | 87 | -0.395 | -1.618 | 0.002 | 0.246 | 1 | 5302 | tags=52%, list=30%, signal=74% |
| HSA00562_INOSITOL_PHOSPHATE_METABOLISM | 42 | -0.447 | -1.606 | 0.014 | 0.247 | 1 | 4954 | tags=57%, list=28%, signal=79% |
| MOTOR_ACTIVITY | 25 | -0.512 | -1.604 | 0.025 | 0.247 | 1 | 2994 | tags=52%, list=17%, signal=63% |
| CELL_CYCLE | 78 | -0.396 | -1.619 | 0 | 0.248 | 1 | 5302 | tags=49%, list=30%, signal=70% |
